# The Core Components of Organelle Biogenesis and Membrane Transport in the Hydrogenosomes of *Trichomonas vaginalis*

**DOI:** 10.1371/journal.pone.0024428

**Published:** 2011-09-15

**Authors:** Petr Rada, Pavel Doležal, Petr L. Jedelský, Dejan Bursac, Andrew J. Perry, Miroslava Šedinová, Kateřina Smíšková, Marian Novotný, Neritza Campo Beltrán, Ivan Hrdý, Trevor Lithgow, Jan Tachezy

**Affiliations:** 1 Department of Parasitology, Charles University in Prague, Faculty of Science, Prague, Czech Republic; 2 Laboratory of Mass Spectrometry, Charles University in Prague, Faculty of Science, Prague, Czech Republic; 3 Department of Biochemistry & Molecular Biology, Monash University, Melbourne, Australia; Newcastle University, United Kingdom

## Abstract

*Trichomonas vaginalis* is a parasitic protist of the Excavata group. It contains an anaerobic form of mitochondria called hydrogenosomes, which produce hydrogen and ATP; the majority of mitochondrial pathways and the organellar genome were lost during the mitochondrion-to-hydrogenosome transition. Consequently, all hydrogenosomal proteins are encoded in the nucleus and imported into the organelles. However, little is known about the membrane machineries required for biogenesis of the organelle and metabolite exchange. Using a combination of mass spectrometry, immunofluorescence microscopy, *in vitro* import assays and reverse genetics, we characterized the membrane proteins of the hydrogenosome. We identified components of the outer membrane (TOM) and inner membrane (TIM) protein translocases include multiple paralogs of the core Tom40-type porins and Tim17/22/23 channel proteins, respectively, and uniquely modified small Tim chaperones. The inner membrane proteins TvTim17/22/23-1 and Pam18 were shown to possess conserved information for targeting to mitochondrial inner membranes, but too divergent in sequence to support the growth of yeast strains lacking Tim17, Tim22, Tim23 or Pam18. Full complementation was seen only when the J-domain of hydrogenosomal Pam18 was fused with N-terminal region and transmembrane segment of the yeast homolog. Candidates for metabolite exchange across the outer membrane were identified including multiple isoforms of the β-barrel proteins, Hmp35 and Hmp36; inner membrane MCF-type metabolite carriers were limited to five homologs of the ATP/ADP carrier, Hmp31. Lastly, hydrogenosomes possess a pathway for the assembly of C-tail-anchored proteins into their outer membrane with several new tail-anchored proteins being identified. These results show that hydrogenosomes and mitochondria share common core membrane components required for protein import and metabolite exchange; however, they also reveal remarkable differences that reflect the functional adaptation of hydrogenosomes to anaerobic conditions and the peculiar evolutionary history of the Excavata group.

## Introduction

Hydrogenosomes are highly divergent forms of mitochondria adapted for ATP synthesis under anaerobic conditions with the concomitant production of molecular hydrogen [1]. These organelles are present in pathogenic and free-living unicellular eukaryotes that inhabit oxygen-poor environments [2]. In the course of the mitochondria-to-hydrogenosome transition, aspects of typical mitochondrial energy metabolism were lost, including the classic pyruvate dehydrogenase complex, the citric acid cycle and the elaborate membrane-associated respiratory chain. Given the absence of genes encoding the membrane subunits of respiratory complexes, which are invariably coded by the mitochondrial genome (e.g., cytochrome oxidase subunit Cox1 and cytochrome b), perhaps this is the reason that hydrogenosomal genomes were relinquished [3], [4]. To synthesize ATP, hydrogenosomes have gained specific pathways that metabolize pyruvate or malate to acetate and CO_2_ and hydrogen in a process accompanied by substrate-level phosphorylation [1].

One of the major mitochondrial functions is to supply other cellular compartments with metabolic energy. The evolution of ADP/ATP carriers (AACs) provided a means to mediate the export of ATP across the mitochondrial inner membrane in exchange for ADP. The function of AACs is coupled with a specific family of porins called voltage-dependent anion channels (VDACs) that passively allow a nucleotide flux across the outer membrane [5]. In addition to AACs, the mitochondrial inner membrane possesses up to 55 distinct carriers that belong to a large mitochondrial carrier protein family (MCF) [6]–[11]. These carriers facilitate the exchange of a wide variety of metabolites to connect cytosolic and mitochondrial metabolism [12], [13]. MCFs and VDACs are nuclearly encoded proteins that are synthesized in the cytosol and targeted to a translocase in the outer mitochondrial membrane (TOM) complex. The TOM complex is the main gate for the entry of mitochondrial proteins into the intermembrane space, where they are further sorted according to their final destination. The porin precursors that are targeted to the outer membrane are assembled by sorting and assembly machinery (SAM complex). The AACs and other MCFs are assembled by a protein translocase in the inner mitochondrial membrane (TIM) complex. In many eukaryotes, there are two distinct TIM complexes [14], [15] that are built from distinct members of the Tim17/Tim22/Tim23 family of proteins. In this case, the MCFs are assembled by the TIM22 complex [16]–[19], whereas proteins transferred into the matrix are assembled by the TIM23 complex in a process catalyzed by the presequence translocase-associated motor (PAM) complex.

Our knowledge about the proteins in hydrogenosomal membranes that facilitate protein transport and the exchange of metabolites is in its infancy. The most-studied hydrogenosomes are those in the human pathogen, *Trichomonas vaginalis*, for which the complete genome sequence is available [20]. However, only two hydrogenosomal membrane proteins, i.e., Hmp31 and Hmp35, have been described in this organism thus far. Hmp31 is a MCF member and serves as an AAC carrier localized in the inner hydrogenosomal membrane [21]–[24]. The cysteine-rich Hmp35 protein is predicted to form pores but has no known homologs; its precise function is unknown. Despite the paucity of knowledge on membrane proteins, a number of proteins have been localized in the matrix of hydrogenosomes. The targeting of matrix proteins is dependent on N-terminal cleavable presequences [25], [26] or internal targeting signals [27]. The presequences are removed in the hydrogenosomal matrix by a dimeric hydrogenosomal processing peptidase that shares a common origin with the mitochondrial processing peptidase, MPP [26].

To gain insight into the processes mediating the exchange of metabolites and the protein import machinery in *T. vaginalis* hydrogenosomes, we established a proteomics survey of the organelle. We sought to determine how many outer membrane porins and inner membrane MCF-like carriers are present in the hydrogenosomes, what the spectrum of other hydrogenosomal multitopic and monotopic membrane proteins is, and whether any components of the protein import machinery have been overlooked by previous bioinformatic-only searches [14], [20]. The proteomic approach, together with bioinformatics, biochemical assays and fluorescence microscopy, allowed the identification and validation of an unusually large number of β-barrel proteins, including several paralogs of Tom40, Sam50 and Hmp35, whereas the spectrum of inner membrane carriers was apparently limited to the AAC types of MCF. Two selected components of the inner membrane translocase, Tim17-22-23A and Pam18, were identified, and their efficient assembly into yeast mitochondrial membranes suggests the conservation of membrane targeting signals for these inner membrane proteins. However, the extreme divergence of four hydrogenosomal Tim17-22-23 family proteins obscures the determination of whether distinct TIM23 and TIM22 complexes are both present in the hydrogenosomes. Lastly, we identified two small Tim chaperones with previously unseen modifications that adapt them to function in the unique anaerobic conditions of the hydrogenosomes in *T. vaginalis*.

## Results

### Identification of hydrogenosomal membrane proteins of diverse topologies

**Figure 1:**
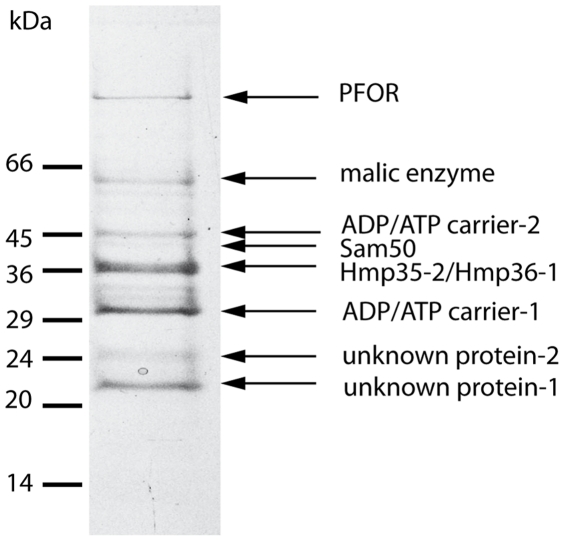
Proteins extracted by Triton-X114 from hydrogenosomal membranes. The most abundant proteins observed by electrophoresis after Triton X-114 extraction included seven integral membrane proteins (ADP/ATP carrier-1, ADP/ATP carrier-2, Hmp35-2, Hmp36-1, Sam50, unknown protein-1 TVAG_455090, and unknown protein-2 TVAG_440200), an integral monotopic proteins (pyruvate:ferredoxin oxidoreductase, PFOR), and malic enzyme, which is the dominant hydrophobic protein in hydrogenosomes.

Hydrogenosomes were purified by differential and Percoll-gradient centrifugation from a lysate of *T. vaginalis*, and membrane proteins were extracted from the purified hydrogenosomes using Triton X-114. The extracted proteins were separated by 1D SDS-PAGE (Fig. 1), the gel lane was cut into 47 slices, and each slice was submitted to nanoLC MS/MS analysis (Table S1). The sequences of the identified proteins were analyzed by a range of bioinformatic tools designed for the detection of conserved domains using multiple sequence alignments and hidden Markov models, structure predictions, and predictions of subcellular localization (Tables 1A,1B and S2). We identified 68 putative membrane proteins; we annotated 17 of these as components of protein import machinery, including key components of the outer membrane TOM and inner membrane TIM complexes. In addition, 11 polytopic transmembrane proteins and 44 integral monotopic proteins were identified, as judged by transmembrane prediction algorithms (see the Materials and Methods). The most abundant proteins observed by electrophoresis after Triton X-114 extraction included eight integral membrane proteins: ADP/ATP carrier-1, ADP/ATP carrier-2, Hmp35-2, Hmp36-1, Sam50, hypothetical proteins TVAG_455090 and TVAG_440200, and pyruvate:ferredoxin oxidoreductase, in addition to the most abundant soluble protein in the hydrogenosome, i.e., malic enzyme (Fig. 1).

**Table 1:** Putative membrane proteins identified in *T. vaginalis* hydrogenosomes.

Identification		Structure		Cell localization			Signal
Accession number	Name	TMHMM	MEMSAT3	TargetP	PsortII	Exp. Local.	
		TM No.	TM No.		mit%		
**Protein import machinery**							
TVAG_399510	Tom40-1	0	0	O	17.4%	NV	β
TVAG_332970	Tom40-2	0	0	O	*		β
TVAG_450220	Tom40-3	0	0	O	17.4%	H	β
TVAG_123100	Tom40-4	0	0	O	4.3%	NV	β
TVAG_341190	Tom40-5	0	0	O	8.7%	H	β
TVAG_195900	Tom40-6	0	0	O	4.3%	H	β
TVAG_178100	Sam50	0	0	O	17.4%	H	β
TVAG_287510	small Tim9-10A	0	0	O	8.7%	H	
TVAG_026080	small Tim9-10B	0	0	O	4.3%	H	
TVAG_198350	Tim17/22/23A	0	0	O	8.7%	H	
TVAG_061900	Tim17/22/23B	2	0	O	17.4%	H	
TVAG_370860	Tim17/22/23C	0	0	O	26.1%	H	
TVAG_379950	Tim17/22/23D	3	0	O	8.7%		
TVAG_447580	Tim17-like	0	0	M	4.3%		
TVAG_008790	Tim44	0	0	M	17.4%	H	
TVAG_470110	Pam16	0	0	O	17.4%	H	
TVAG_436580	Pam18	0	1	O	13.0%	H	Δ
**beta-barrel proteins**							
TVAG_146920	Porin-1	0	0	O	4.3%		β
TVAG_340380	Porin-2	0	0	O	21.7%		β
TVAG_590550	Hmp-35-1	0	0	O	8.7%	H[^50^]	β
TVAG_104250	Hmp-35-2	0	0	O	8.7%	H	β
TVAG_031860	Hmp-36-1	0	0	O	4.3%		
TVAG_216170	Hmp-36-2	0	0	O	8.7%		
**Integral polytopic proteins**							
TVAG_237680	ADP/ATP carrier-1, Hmp-31	0	5	O	26.1%	H[^21^]	
TVAG_051820	ADP/ATP carrier-2	0	6	O	34.8%	H	
TVAG_164560	ADP/ATP carrier-3	0	4	O	8.7%		
TVAG_196220	ADP/ATP carrier-4	0	5	O	13.0%		
TVAG_262210	ADP/ATP carrier-5	0	5	O	13.0%		
TVAG_039960	Unknown	6	6	S	11.1%	H	
TVAG_455090	Unknown	2	1	O	17.4%	H	
TVAG_489980	Unknown	6	3	O	4.3%		
TVAG_127990	Unknown	2	2	O	21.7%		
TVAG_440200	Unknown	3	2	O	4.3%		
TVAG_136450	Unknown	0	2	O	8.7%		
TVAG_192370	Unknown	2	1	O	11.1%	H	

**Figure 2:**
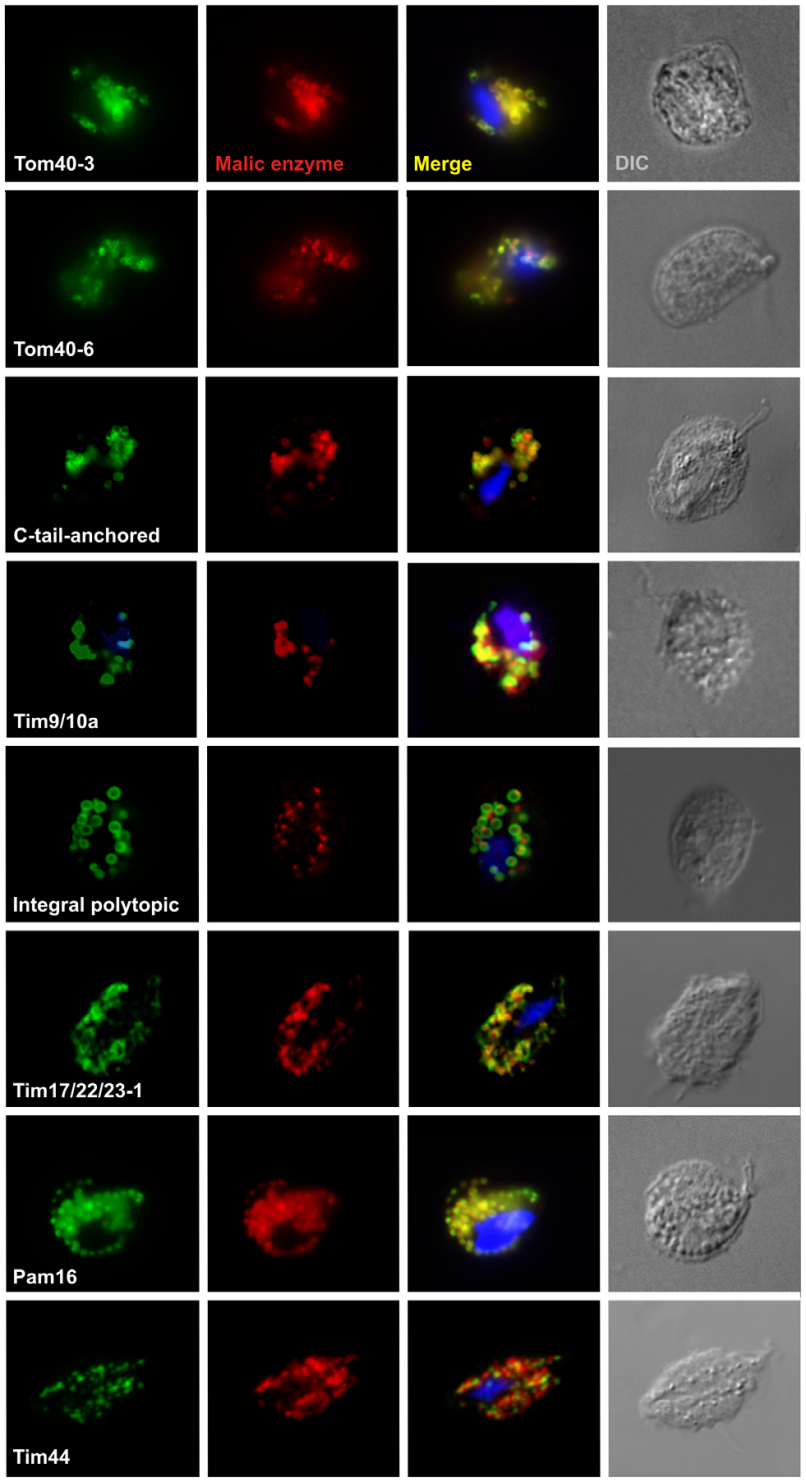
Immunofluorescent microscopy of *T. vaginalis* strains expressing selected membrane proteins. Hemagglutinin-tagged proteins were visualized using an anti-hemagglutinin mouse monoclonal antibody (in green). The matrix protein, malic enzyme, was visualized using a rabbit polyclonal anti-malic antibody (in red). The nucleus was stained with DAPI (blue). DIC, differential interference contrast. Tom40-3 (TVAG_450220), Tom40-6 (TVAG_195900), C-tail-anchored (TVAG_277930), Tim9/10a (TVAG_287510), integral polytopic (TVAG_455090), Tim17/22/23-1 (TVAG_198350), Pam16 (TVAG_470110), Tim44 (TVAG_008790).

To validate the proteomic and sequence analysis, we chose 26 of these putative hydrogenosomal membrane proteins and tested their localization using the expression of epitope-tagged constructs in *T. vaginalis* (Fig. 2 and Table 1,2). Immunofluorescent microscopy revealed the colocalization of tagged proteins and the hydrogenosomal marker protein, malic enzyme. The membrane proteins were often observed at the peripheral rings surrounding the hydrogenosomal matrix labeled by the anti-malic enzyme antibody.

**Table 2:** Putative membrane proteins identified in *T. vaginalis* hydrogenosomes (continued).

Identification		Structure		Cell localization			Signal
Accession number	Name	TMHMM	MEMSAT3	TargetP	PsortII	Exp. Local.	
		TM No.	TM No.		mit%		
**Integral monotopic C-tail-anchored proteins**							
TVAG_090120	C-tail-1	1	1	O	13.0%		Ct
TVAG_190830	C-tail-2	1	0	O	13.0%		Ct
TVAG_458060	C-tail-3	1	1	O	4.3%		Ct
TVAG_272350	C-tail-4	1	0	O	13.0%	H	Ct
TVAG_240680	C-tail-5	1	1	O	13.0%		Ct
TVAG_137270	C-tail-6	1	1	O	8.7%		Ct
TVAG_277930	C-tail-7	1	1	O	13.0%	H	Ct
TVAG_283120	C-tail-8	1	1	O	13.0%	H	Ct
TVAG_174010	C-tail-9	1	1	O	13.0%		Ct
TVAG_369980	C-tail-10	1	1	O	4.3%	H	Ct
TVAG_393390	C-tail-11	1	1	O	*		Ct
TVAG_211970	C-tail-12	1	0	O	13.0%		Ct
**Integral monotopic single spanning proteins**							
TVAG_032990	Unknown	0	1	O	21.7%		
TVAG_080160	Unknown	0	1	O	13.0%		
TVAG_094480	Unknown	1	1	O	17.4%		
TVAG_152710	Unknown	0	1	O	13.0%		
TVAG_178320	Unknown	0	1	O	8.7%		
TVAG_182990	Unknown	0	1	O	21.7%		
TVAG_210010	Unknown	0	1	O	*		
TVAG_218130	Unknown	1	1	S	*		
TVAG_225560	Unknown	1	0	O	21.7%		
TVAG_251750	Unknown	1	1	O	4.3%		
TVAG_252220	Unknown	0	1	O	*		
TVAG_295140	Unknown	0	1	O	*		
TVAG_331680	Unknown	1	1	S	11.1%		
TVAG_333160	Unknown	1	1	O	22.2%		
TVAG_337270	Unknown	0	1	O	26.1%		
TVAG_341690	Unknown	0	1	O	*		
TVAG_370950	Unknown	0	1	M	21.7%		
TVAG_403380	Unknown	0	1	O	33.3%		
TVAG_413430	Unknown	0	1	O	13.0%		
TVAG_425430	Unknown	0	1	O	8.7%		
TVAG_423530	Unknown	1	2	O	17.4%	H	

Proteins were manually annotated based on searches in TrichDB, Uniprot, and PFAM A+B (Table S2). Protein structure was predicted using TMHMM and MEMSAT3; subcellular location was predicted using TargetP and PsortII. TM No., number of predicted transmembrane α-helixes. M, predicted location in mitochondria. S - predicted proteins of secretory pathway; O, predicted location in other compartments; Mit%, probability percentage of mitochondrial location; *, mitochondrial location was not predicted; Exp. Local., experimental location; H, localization of HA-tagged proteins was confirmed in *T. vaginalis* hydrogenosomes by immunofluorescence microscopy; NV, transformed *T. vaginalis* strain was not viable. Signal: Δ indicates N-terminal targeting sequence identified by Hunter; β indicates presence of beta signal of beta-barrel proteins for insertion into the outer membrane of mitochondria; Ct, C-tail anchor detected [Fig. S2].

The proteomic survey identified the products of 63 genes that were either previously annotated as encoding ‘hydrogenosomal proteins’ with known matrix localization or novel hydrogenosomal matrix proteins, such as alanine aminotransferase, phosphofructokinase, hybrid cluster proteins, and Ind-1 (Tables S3A-C), and the protein products of 45 genes that were previously annotated as encoding ‘hypothetical proteins’ with unclear localization (Tables S4C and S4D). The hydrogenosomal preparation also contained 52 proteins annotated as being found in other cellular locations; some of these identified proteins may represent contamination by other membranes (e.g., the ABC transporter, MFS transporter and vacuolar proton ATPase) or cytoplasmic adherence on the hydrogenosomes (e.g., cytosolic HSP70 and cytoskeletal proteins), whereas many were simply inferred to be located elsewhere based on minimal sequence similarity to proteins from other eukaryotes (Tables S3A–C and S4A–D).

### Polytopic proteins of the mitochondrial carrier family

**Figure 3:**
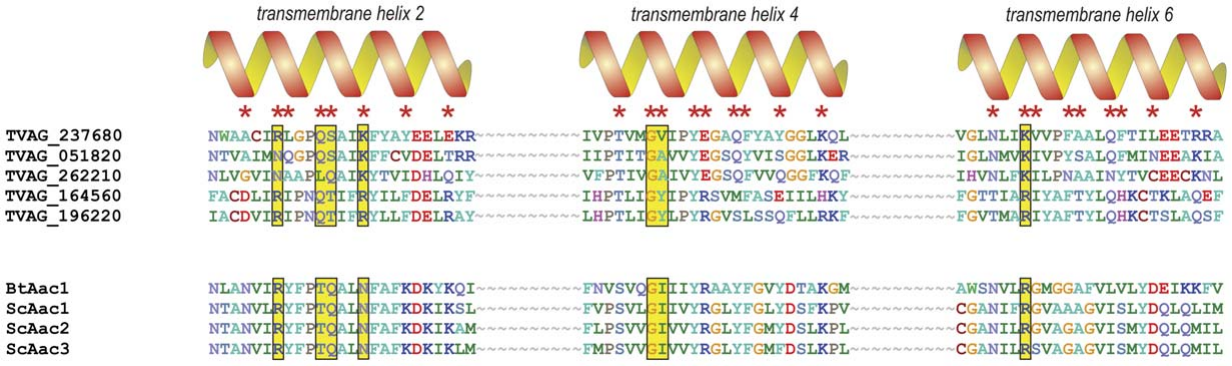
The amino acid sequences of six putative hydrogenosomal carrier proteins were aligned with the sequences of 33 mitochondrial carriers from *Saccharomyces cerevisiae* and that of the AAC1 carrier from *Bos taurus* (Pebay-Peyroula et al., 2003), according to the method of Kunji and Robinson (2006). The substrate specificity of the carrier is determined by the amino acid residues of the even-numbered transmembrane helixes facing the transport pore (red stars). The residues in direct contact with the substrate are highlighted in yellow boxes. Only the even-numbered helixes of the most similar homologs are shown, indicating that TVAG_237680, TVAG_051820, TVAG_262210, TVAG_164560 and TVAG_196220 belong to the group of adenine nucleotide carriers. The corresponding residues of TVAG_197670 do not resemble any of the specificity motifs (Kunji and Robinson, 2006). BtAac1 and ScAac1, ScAac2 and ScAac3 are adenine nucleotide carriers from *Bos taurus* and *Saccharomyces cerevisiae*, respectively.

Multiple transmembrane domains were predicted in 12 of the identified proteins. Of these, five were classified as MCF members: one of these proteins is Hmp31 [21], and the other four have not been previously studied and have no obvious orthologs in other organisms. It was predicted that all five of the hydrogenosomal MCF proteins contain the characteristic six transmembrane alpha-helices. As with all carrier proteins, in these five hydrogenosomal proteins, the odd-numbered helices contain P-X-[DE]-X-X-[KR] signature motifs that, in the context of the three-dimensional structure, surround the pore and determine the substrate specificity of the carrier [28] (Fig. 3). An ADP-ATP exchange activity has been determined for Hmp31 in *Trichomonas gallinae*, and the other carrier proteins we identified in hydrogenosomes have sequence motifs (such as the RRRMMM signature; Fig. S1) that suggest that they also mediate nucleotide exchange.

### Single-spanning and C-tail-anchored proteins

According to the structure predictions, 33 putative hydrogenosomal proteins were classified as integral monotopic proteins with a single hydrophobic transmembrane domain (TMD). Of these proteins, 21 contain a TMD located in the middle of the protein, with N- and C-terminal flanking regions (single-spanning proteins), whereas 12 proteins have characteristics of C-tail anchored proteins (Table 2). The genes corresponding to the single-spanning proteins and C-tail-anchored proteins were previously annotated as encoding ‘hypothetical proteins’ with no significant homology in other organisms.

C-tail-anchored proteins consist of a large functional domain exposed to the cytosol and a short C-terminal transmembrane segment that is flanked at both ends by positively charged residues [29]–[31]. The predicted transmembrane segments of the hydrogenosomal C-tailed proteins are 19–23 amino acid residues in length, which is somewhat longer than those found in mitochondrial proteins. This increased length may reflect differences in the thickness of the lipid bilayer in the outer membranes of each organelle. The C-terminal ends that follow the transmembrane segments are 2–16 amino acid residues in length and contain 2–7 positively charged residues (Fig. S2).

**Figure 4:**
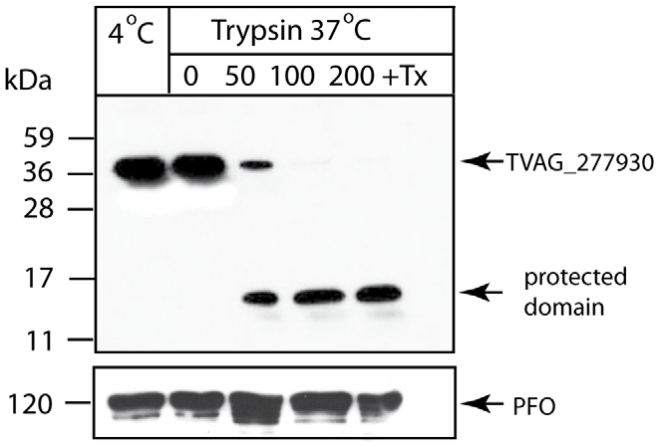
Expression of the C-tail anchored protein, TVAG_277930, in the hydrogenosomal outer membrane. Hydrogenosomes were isolated from a *T. vaginalis* strain expressing TVAG_277930 with a C-terminal hemagglutinin tag and incubated for 30 min at 4°C and 37°C with 0, 50, 100 or 200 µg/ml trypsin or µg/ml trypsin plus 0.5% Triton X-100 (Tx). Samples were analyzed by SDS-PAGE and immunoblotting using a mouse monoclonal anti-HA tag and anti-PFO antibodies. Pyruvate:ferredoxin oxidoreductase (PFO) was used as a control matrix proteins.

To validate the predicted topology of the C-tailed anchored protein TVAG_ 277930, we added a C-terminal HA tag and expressed the modified protein in *T. vaginalis*. We then confirmed that the expressed protein was targeted to hydrogenosomes by immune-fluorescent microscopy (Table 2). Hydrogenosomes from this transformed strain were isolated and treated with trypsin. Although proteolysis did not affect the mobility of the matrix protein, pyruvate:ferredoxin oxidoreductase, which is protected by the hydrogenosomal membranes (Fig. 4), proteolysis resulted in a shift of Tta1 mobility on SDS-PAGE from 36 kDa to ∼14 kDa. This result is consistent with the expected cleavage of the ∼22 kDa domain facing the cytosol, with the C-terminal domain and epitope-tag protected from trypsinolysis by the outer membrane. The C-terminal domain of this C-tail anchored protein was degraded only when Triton X-100 was added to solubilize the outer membrane (Fig. 4).

### Mitochondrial porins and other outer membrane β -barrel proteins

Tom40, an essential component of the TOM complex, is the main gate for membrane proteins imported into the mitochondria [16]. Tom40 is a β-barrel protein and, together with VDAC, belongs to the Pfam family, PF01459, whose members are also referred to as the ‘mitochondrial porins’ [32]. Recent structural studies have shown that the β-barrels of the mitochondrial porins are assembled from 19 beta-strands [33], [34]. In hydrogenosomal preparations, we identified 8 proteins that we designate as mitochondrial porins because they have sequence features of the PF01459 family, and secondary structure predictions using the PSI-PRED algorithm suggest that all 8 of the sequences contain 19 transmembrane beta strands (Fig. S3). The very last beta strand of all of the known mitochondrial outer membrane β-barrel proteins contains a beta signal motif, PxGxxHxH, where P stands for polar amino acid residue, G for glycine and H for hydrophobic acid. The signal is recognized by the SAM complex to facilitate the assembly of these proteins in the outer membrane [35]. All eight of the *T. vaginalis* mitochondrial porins contain this conserved motif (Fig. S3).

To distinguish whether the identified β-barrel proteins represent Tom40 or VDAC homologs, we performed independent HMM-based searches of predicted *T. vaginalis* proteins based on *T. vaginalis* genome sequences. Tom40 and VDAC HMMs were built from the protein sequences of an identical set of species. The Tom40 HMM search identified 6 of the 8 mitochondrial porins, which we therefore named Tom40-(1 through 6). No sequences were matched using the VDAC-specific HMM search under the HMMER 2 default parameters. It remains possible that one or more of the remaining mitochondrial porins functions as a VDAC and has a sequence that is too highly diverged to be aligned with the VDAC sequences from other eukaryotes.

**Figure 5:**
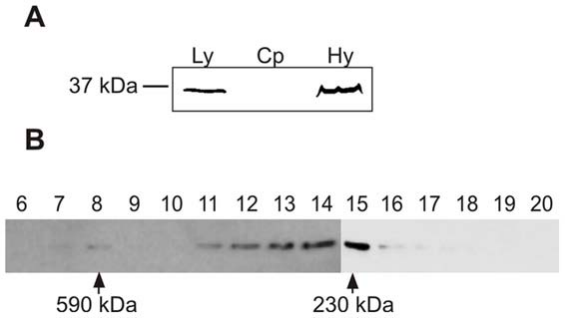
Cellular localization and gel filtration chromatography of trichomonad Tom40-3. (A) Total cell lysates (Ly) and cytosolic (Cp) and hydrogenosomal fractions (Hy) were prepared from trophozoites expressing an HA-tagged Tom40-3 (TVAG_450220) and analyzed by SDS-PAGE and immunoblotting using a mouse monoclonal anti-HA tag antibody. (B) Size exclusion chromatography of Tom40-3. Hydrogenosomes were solubilized in gel filtration running buffer containing 0.5% TX-100 and centrifuged, and the supernatant was subjected to gel filtration on a Superdex-200 column. Fractions of 500 µl (6-20) were analyzed by immunoblotting. Estimated sizes of the components of the putative trichomonad Tom40 complex are marked with arrows.

Given that the TOM complex is a multi-subunit molecular machine built around a Tom40 channel, we tested whether *T. vaginalis* Tom40 was also found as a part of a high-molecular-weight complex. We engineered a strain of *T. vaginalis* to express a HA-tagged version of Tom40-3 and verified that the protein was localized to hydrogenosomes (Fig. 5A). Hydrogenosomes were isolated from the transformed parasites, gently solubilized by 0.5% Triton X-100 and analyzed by gel filtration. Immunoblotting of the elution profile revealed a major peak of Tom40-3 present as oligomers of ∼230 kDa, with a smaller population of Tom40-3 in a complex of ∼590 kDa (Fig. 5B). These results are consistent with the mitochondrial porin, Tom40-3, being a subunit of a protein complex that assists hydrogenosomal protein import.

Hmp35 (hydrogenosomal membrane protein 35) is a unique form of a β-barrel protein identified in hydrogenosomes. The proteomic assessment of the hydrogenosomes identified what appears to be a second isoform of Hmp35 (Hmp35-2) and another two related proteins that are distinguished by a C-terminal extension (Hmp36-1 and Hmp36-2). The Hmp35 and Hmp36 proteins are encoded from four different genes in *T. vaginalis*; excluding the C-terminal extension, their DNA sequences are sufficiently similar to strongly indicate very recent gene duplications and a likely functional redundancy of the proteins. Previously, Hmp35 was predicted to be predominantly composed of beta sheets [36], and current PSIPRED predictions (Fig. S4) indicate that the polypeptide chain is arranged in up to 19 beta sheets, plus one alpha helix positioned in the middle of the protein sequence between beta sheets 10 and 11. We therefore extend the suggestion by Dyall et al. [36] to conclude that the Hmp35 and Hmp36 family of proteins are outer membrane β-barrels. A distinguishing feature of the Hmp35-1 and Hmp35-2 proteins is their cysteine-rich character, including a C-terminal domain that features the metal-binding motif, CX_6_CCX_2_CX_9_HX_15_CCXHXX_2_C [36]. The Hmp36-1 and Hmp36-2 proteins both lack this C-terminal cysteine-rich domain, and Hmp36-1 and Hmp36-2 contain only 2 and 5 cysteine residues, respectively. Although the last predicted beta-strand of Hmp35-1 and Hmp35-2 shows a match to the beta-signal motif, this motif is not clear in Hmp36-1 and Hmp36-2.

### Small TIM chaperones associated with hydrogenosomal membranes

**Figure 6:**
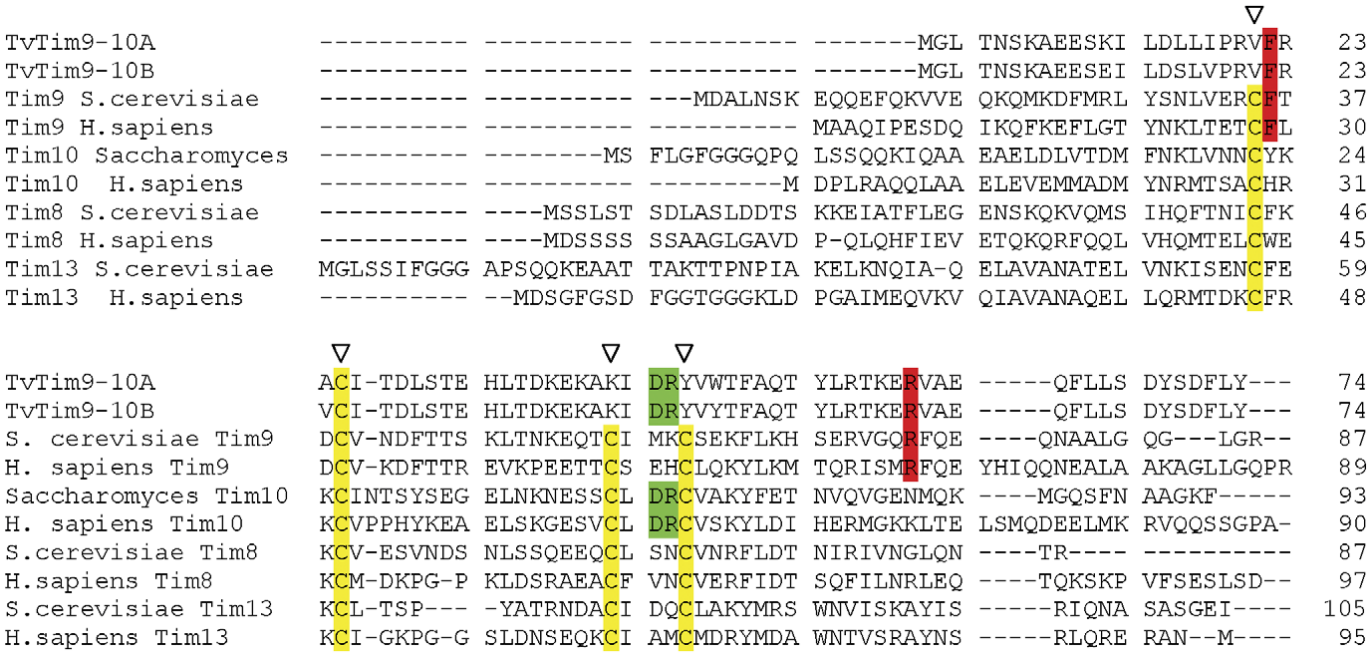
Sequence alignment of the *Trichomonas vaginalis* small Tims, TvTim9-10A (TVAG_287510) and TvTim9-10B (TVAG_026080) against the sequences of *S. cerevisiae* and human members of four small Tim families. Tim9: *S. cerevisiae*, O74700; *H. sapiens*, Q9Y5J7. Tim10: *S. cerevisiae*, P87108; *H. sapiens*, P62072. Tim8: *S. cerevisiae*, P57744; *H. sapiens*, O60220. Tim13: *S. cerevisiae*, P53299; *H. sapiens*, AAF15101. The conserved cysteine residues are marked by arrowheads (▿) and highlighted in yellow. The conserved Tim9 residues are highlighted in red, and conserved Tim10 residues are highlighted in green.

Integral membrane proteins of the outer and inner membranes of mitochondria are assembled into the membranes with the assistance of a group of ∼10 kDa chaperones called small TIMs. These chaperones are localized within the intermembrane space and are also found associated with membranes, reflecting their role in the delivery of nascent imported membrane proteins [37]–[39]. Sequence classifications have demonstrated that there are four small TIM families: Tim8, Tim9, Tim10 and Tim13 [40]. These families reflect the structural characteristics required to form hetero-hexameric functional chaperones of the type Tim8_3_:Tim13_3_ or Tim9_3_:Tim10_3_. In the hydrogenosomal membrane fraction, we identified two small TIM proteins that share 93% sequence identity (Fig. 6). The main difference between these paralogs is at position 12, where either a negatively-charged glutamic acid (TvTim9/10a) or positively charged lysine (TvTim9/10b) is present. A comparative analysis of the hydrogenosomal small TIMs is made difficult by sequence divergence, but the highest sequence identity for TvTim9/10a and TvTim9/10b was found with Tim9 of *S. cerevisiae.* An extraordinarily distinguishing feature of both hydrogenosomal proteins is the absence of the defining conserved twin cysteine motif, CX_3_CX_n_CX_3_C: TvTim9-10a and TvTim9-10b retain only a single cysteine (Cys25) (Fig. 6).

### Proteins of the Tim17/22/23 family and the hydrogenosomal TIM complexes

**Figure 7:**
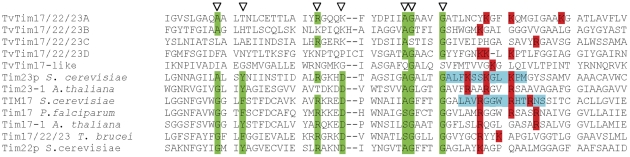
Alignment of the *T. vaginalis* Tim17/22/23 PRAT domains against eukaryotic orthologs. Organism names and accession numbers are as follows: *T. vaginalis* TvTim17/22/23A, TVAG_198350; TvTim17/22/23B, TVAG_061900; TvTim17/22/23C, TVAG_370860; TvTim17/22/23D, TVAG_379950; TvTim17-like, TVAG_447580; *A. thaliana* Tim23-1, AAR26373; *S. cerevisiae*, Tim17, CAA89438; *P. falicparum*, putative Tim17, XP_001348502; *A. thaliana*, Tim17-1, AAO63303; *T. brucei* Tim17/22/23 CBH18364; *S. cerevisiae* Tim22p NP_010064. Consensus PRAT sequence: G/AX_2_F/YX_10_RX_3_DX_6_ G/A/SGX_3_G. Conserved residues are marked by arrowheads and highlighted in green. Experimentally verified internal targeting signals are in blue. Positively charged residues at the C-terminus are in red.

The key components of the inner membrane translocase complexes, TIM23 and TIM22, belong to the Tim17/Tim22/Tim23 protein family. These proteins typically possess four transmembrane domains and an amino acid signature called the PRAT motif [41]. As characterized in yeast, the Tim17 and Tim23 proteins constitute the TIM23 complex that translocates proteins into the matrix, whereas the protein called Tim22 forms a distinct TIM22 complex to facilitate the import of MCFs and other multitopic proteins. The proteomics survey identified two hydrogenosomal proteins, TvTim17/22/23-1 and TvTim17/22/23-2, which match significantly with the Tim17/22/23 family in the PFAM database (E = 1,7^−18^ and E = 2,3^−14^; respectively). In addition, we found three other hydrogenosomal membrane proteins with some overall sequence similarity to the family but with insignificant matches (E>7,2^−6^) (Table 1). We refer to these three proteins as TvTimC, TvTimD and TvTim-like. The PRAT domain was partially conserved in TvTim17/22/23-1 and TvTim17/22/23-2, and it was conserved to a lesser extent in TvTimC and TvTim D and was absent in the TvTim-like protein. We suggest that TvTim17/22/23-1 and TvTim17/22/23-2 should be considered as candidate TIM complex subunits and that TvTimC and TvTimD should be considered as potential candidates (Fig. 7). We note also that TvTim17/22/23-1 possesses positively charged residues at its C-terminus (Lys130 and Lys131) and negatively charged residues at its N-terminus (Glu26 and Glu27), which are features of Tim23 [42]. However, limited conservation of the hydrogenosomal proteins in any of the Tim17, Tim22 and Tim23 subfamilies prevents a determination of their functional equivalence based on sequence alone. Consistent with this sequence divergence, the expression of TvTim17/22/23-1 or TvTim17/22/23-2 failed to support the growth of yeast strains lacking Tim17, Tim22 or Tim23 (Fig. S5A, B, C).

**Figure 8:**
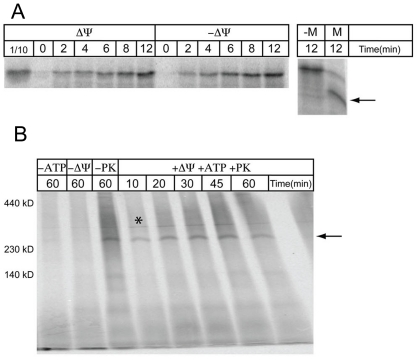
*In vitro* import and topology of TvTim17/22/23-1. (A) *In vitro* synthesized, [S^35^]-radiolabeled TvTim17/22/23-1 was incubated with isolated yeast mitochondria at 25°C for the indicated time, treated with 25 µg/mL proteinase K to degrade surface-associated proteins and analyzed by SDS PAGE and autoradiography. After incubation with the [S^35^]-radiolabeled TvTim17/22/23-1 for 12 minutes, yeast mitochondria were exposed to proteinase K in import buffer (see the Material and Methods) with a hypo-osmotic buffer (10 mM MOPS – “M”) or import buffer alone (“-M”) and resolved by SDS PAGE. The arrow indicates the fragment of TvTim17/22/23-1 protected by the inner mitochondrial membrane. Proteins were detected by autoradiography. Δψ, the reaction as described proceeding in the presence of transmembrane potential; -Δψ, the reaction as described when the transmembrane potential was dissipated by the addition of 1 nM valinomycine. (B) *In vitro* synthesized, [S^35^]-radiolabeled TvTim17/22/23-1 was incubated with isolated yeast mitochondria at 25°C for the indicated time. Proteins were then treated with 25 µg/mL proteinase K to degrade surface-associated proteins or incubated in the absence of externally added ATP (-ATP) when the transmembrane potential was dissipated by the addition of 1 nM valinomycine (-Δψ) or without PK treatment (-PK). One species was detected (∼280 kD) (arrow). (*), abundant respiratory chain complex radiolabeled by free [S^35^] methionine.

To test whether these hydrogenosomal proteins possess the information required for assembly into the inner membrane of yeast mitochondria, we measured the import of TvTim17/22/23-1 into mitochondria isolated from yeast. A time-dependent incorporation of the radiolabeled protein was observed (Fig. 8A), indicating that TvTim17/22/23-1 was imported across the outer membrane. The treatment of the mitochondria in hypo-osmotic buffer before the addition of proteinase K (PK) revealed the presence of a TvTim17/22/23-1 fragment that was ∼3 kDa smaller than the full-length protein, indicating that TvTim17/22/23-1 was incorporated into the inner membrane (and, thereby, largely protected from PK). Whereas no effect on the translocation of TvTim17/22/23-1 across the outer membrane was observed when the membrane potential (Δψ) was dissipated by valinomycine (Fig. 8A), the incorporation of TvTim17/22/23-1 into a high molecular complex (over 230 kDa) was observed, but only when TvTim17/22/23-1 was incubated with energized mitochondria (Fig. 8B). Thus, the translocation of TvTim17/22/23-1 across the outer membrane is independent of Δψ, whereas its insertion into the inner membrane complex depends on Δψ. These properties conform to those observed for Tim17/22/23 proteins from yeast [43].

### Presequence translocase-associated motor (PAM)

The final step of preprotein import across the inner membrane requires the function of the matrix-exposed PAM complex, which consists of two soluble matrix proteins, mtHsp70 and Mge1, and 3 essential membrane components (Tim44, Pam16, and Pam18). A complete set of putative membrane PAM components was identified in hydrogenosomes, and the membrane proteins, TvPam16, TvPam18 and TvTim44, localize to hydrogenosomes when expressed in *T. vaginalis* with a C-terminal HA tag (Table 1). The protein sequence of TVAG_436580 (TvPam18) conforms to the sequence characteristics of HMM for the Pam18 family (Fig. S6) [44]. The characteristic features of Pam16, which forms a subcomplex with Pam18, were identified in protein sequence TVAG_470110 (TvPam16), and the conserved hypothetical protein, TVAG_008790, is a candidate Tim44 (Table 1 and Figs. S7 and S8). It is noteworthy that the N-terminal domains of Tim44 vary in length and structure depending on the species [45], with the TvTim44 N-terminal domain consisting of 144 residues with a predicted hydrogenosomal targeting presequence (Fig. S8).

**Figure 9:**
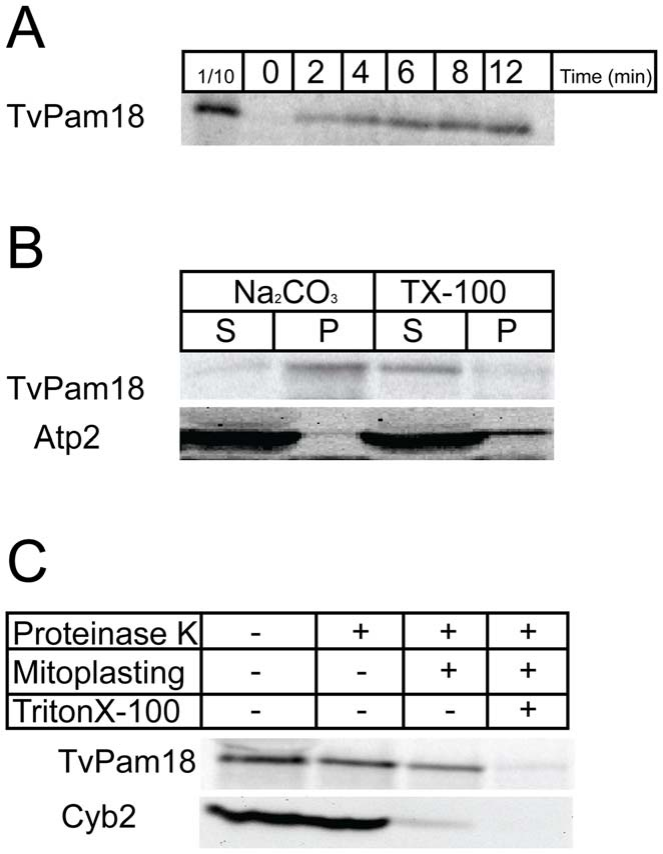
*In vitro* import and the topology of TvPam18. (A) *In vitro* synthesized, [S^35^]-radiolabeled TvPam18 was incubated with isolated yeast mitochondria at 25°C for the indicated time, treated with 25 µg/ml proteinase K to degrade surface-associated proteins and analyzed by SDS PAGE and autoradiography. (B) After incubation with [S^35^]-radiolabeled TvPam18 for 30 minutes, yeast mitochondria were treated with sodium carbonate (pH 11.5) and Triton X-100 and centrifuged at 100,000× g. Samples were resolved by SDS-PAGE, and the proteins were detected by western blotting and autoradiography. (S) soluble fraction; (P) insoluble fraction. (C) After incubation with [S^35^]-radiolabeled TvPam18 or GiPam18 for 30 minutes, yeast mitochondria were exposed to proteinase K in import buffer, hypo-osmotic buffer (10 mM MOPS – “M”) or 1% Triton X-100 (“TX”) and resolved by SDS PAGE. Proteins were detected by western blotting or autoradiography.

We sought to determine whether TvPam18 functions in the PAM complex in yeast. First, we tested whether [^35^S]-labeled TvPam18 accumulated in yeast mitochondria in a time-dependent manner and behaved as an inner membrane protein (Figs. 9A and B). To assess the topology of the imported TvPam18, yeast mitochondria were sequentially treated by proteinase K and a hypo-osmotic buffer. When the outer membrane was ruptured, the intermembrane space protein, Cyb2, was degraded, but TvPam18 was not affected (Fig. 9C). However, when the outer and inner membranes were lysed by Triton X-100, TvPam18 was degraded. Thus, TvPam18 assumes the same topology as the ScPam18 yeast protein does in mitochondria, with an N-terminal transmembrane domain in the inner mitochondrial membrane and its J-domain located in the mitochondrial matrix.

**Figure 10:**
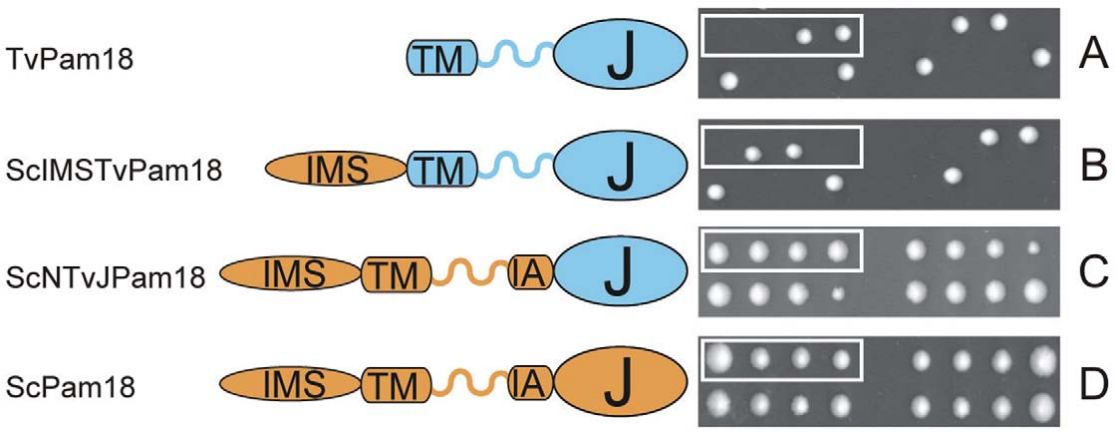
The J-domain of *Trichomonas vaginalis* TvPam18 can substitute for the J-domain of yeast ScPam18. Yeast Pam18/Δ*pam18* cells were transformed with plasmids carrying wild-type or modified Pam18 sequences. Cells were sporulated, and the tetrads were dissected onto YPD plates. Two viable colonies indicate no complementation by the candidate protein; four viable colonies indicate successful complementation by the candidate sequence. (A) TvPam18, wild-type *Trichomonas vaginalis* Pam18 sequence; (B) ScIMSTvPam18, ScPam18 IMS domain fused to wild-type TvPam18; (C) ScNTvJPam18, ScPam18 J-domain replaced by TvPam18 J-domain; (D) ScPam18, wild-type yeast Pam18 sequence; Orange, ScPam18 sequence; Blue, TvPam18 sequence; TM, transmembrane domain; IMS, intermembrane space domain; IA, interaction arm; J, J-domain.

To test whether TvPam18 can function in place of ScPam18, chimeric ScPam18-TvPam18 constructs were expressed in a heterozygous mutant yeast, Pam18/Δ*pam18* (Fig. 10). The heterozygous diploid cells were induced to sporulate, and the tetrads were dissected onto rich media plates. Because PAM18 is an essential gene, the Δ*pam18* haploid progeny should not form viable colonies on the dissection plates. Cells transformed with the plasmid encoding ScPam18 served as a control and showed four viable colonies from each tetrad (Fig. 10D). When TvPam18 was expressed, only two spores germinated to form colonies of haploid cells, indicating that TvPam18 was not able to restore full PAM function in yeast (Fig. 10A). Furthermore, the addition of the specific yeast intermembrane space domain to TvPam18 (ScIMSTvPam18) was not sufficient to bring TvPam18 into the correct context to function in yeast (Fig. 10B). Full complementation was seen only when the yeast N-terminal region and transmembrane segment were fused with the TvPam18 J-domain (ScNTvJPam18) (Fig. 10C). The growth rate and viability of wild-type and cells complemented with ScNTvJPam18 were indistinguishable at 25-30°C when the cells were grown on a fermentable (glucose) or non-fermentable (lactate) carbon source (Fig. S9A), and the efficiency of protein import into the mitochondria where the PAM complex was restored with ScNTvJPam18 was indistinguishable from the activity of ScPam18 (Fig. S9B).

## Discussion

**Figure 11:**
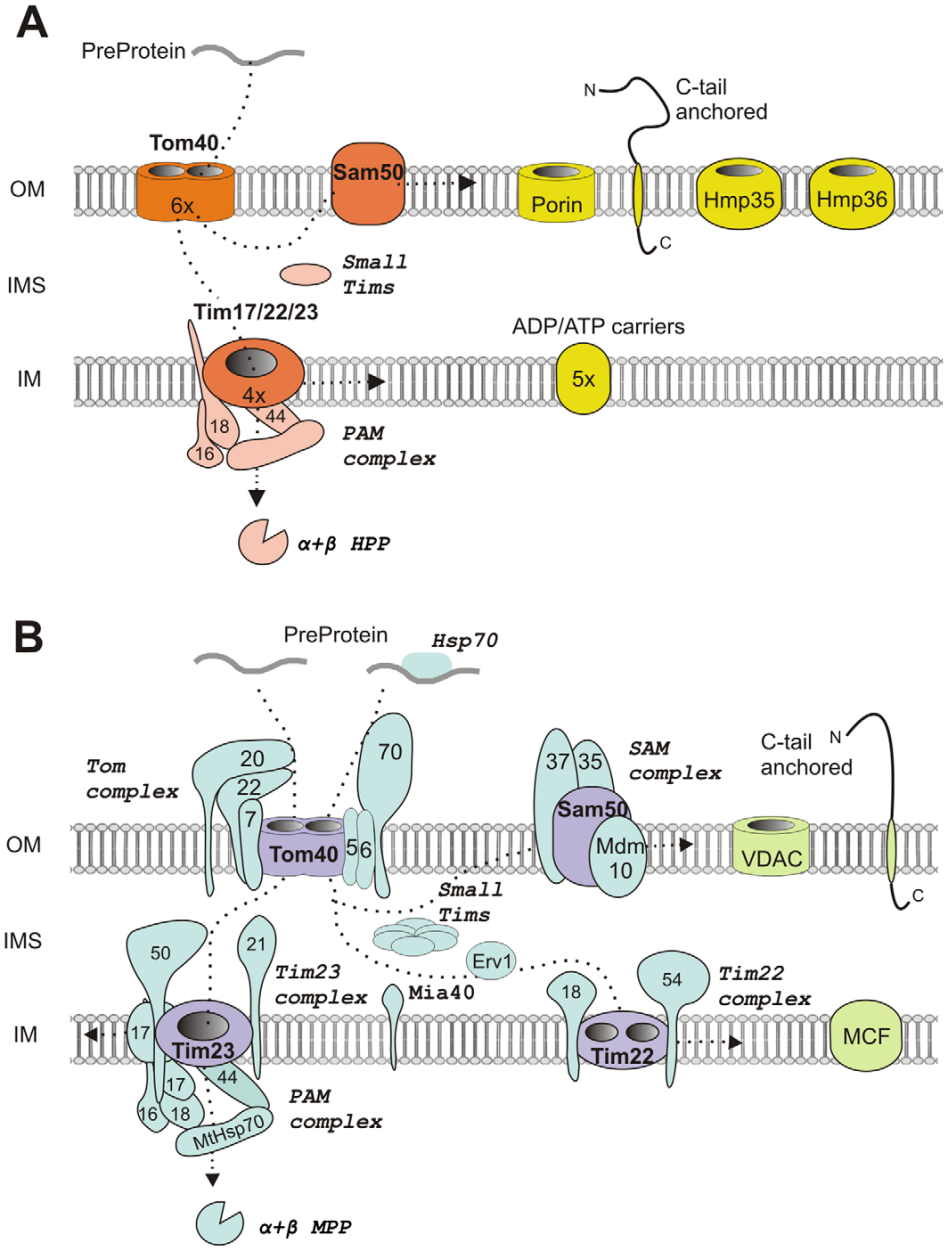
Comparison of the protein machineries of the outer and inner membranes of *T. vaginalis* hydrogenosomes (A) and *S. cerevisiae* mitochondria (B). *T. vaginalis* and *S. cerevisiae* belong to two distinct eukaryotic supergroups: Excavata and Opisthokonta, respectively. Excavates are exclusively unicellular and are often parasitic eukaryotes, whereas opisthokonts include both unicellular and multicellular organisms, such as fungi and animals. *T. vaginalis* hydrogenosomes and yeast mitochondria share core components of the outer and inner membranes (Tom40, Sam50, Tim17/22/23, and PAM machinery), although the protein sequences are extremely divergent. The associated components, such as Tom20 and Tom70, and the inner membrane component, Mia40, were not identified in the hydrogenosomes or other excavates, suggesting that these components were not present in the last common mitochondrial ancestor, although a secondary loss cannot be excluded. Conversely, the absence of Erv1 and the reduction of the small Tims family to a single type of highly modified TvTim9/10 most likely reflect the specific adaptation of hydrogenosomes to anaerobic conditions. The assembly of ADP/ATP within the inner hydrogenosomal membrane and the identification of divergent small Tims and Tim17/22/23 proteins indicate that a functional TIM22 complex is present; however, sequence divergence prevented the prediction of whether the Tim17/22/23 proteins form a single multifunctional channel or distinct TIM23 and TIM22 complexes. In addition to the TOM/TIM machineries, the hydrogenosomes possess a conserved pathway for the assembly of inner membrane C-tail anchored proteins. (A) The core components of the hydrogenosomal protein import machinery are shown in orange, and the subunits of the PAM machinery and the hydrogenosomal processing peptidase, HPP, are in pink. The putative components of the metabolite exchange system and C-tail-anchored protein are shown in yellow. (B) The core components of the mitochondrial protein import machinery are shown in purple, and associated components are depicted in blue. VDACs, C-tail-anchored proteins and MCFs (mitochondrial carrier family) are shown in green.

Mitochondria are surrounded by two distinct membranes, across which metabolites are exchanged to coordinate metabolic pathways in the cytosol with those that act within the organelle. Both the outer membrane and inner membrane possess a specific set of membrane proteins that facilitate this metabolite exchange as well as machineries for protein import, interactions with various cellular structures and other diverse functions. Our analysis of hydrogenosomal membrane proteins revealed the presence of membrane transporters, including major core components required for organelle biogenesis that are functional homologs of the mitochondrial systems, thus extending support for the common evolutionary origin of mitochondria and hydrogenosomes from an ancestral endosymbiont. However, our analysis also revealed remarkable distinctions in the membrane proteome of hydrogenosomes (Fig. 11A, B).

### Outer membrane proteins in hydrogenosomes

The outer hydrogenosomal membrane has proteins of at least two distinct architectures: β-barrel proteins and α-helical, monotopic proteins of tail-anchored topology. Proteins of the mitoporin family of β-barrel proteins include Tom40 (a protein transport pore) and VDAC (a metabolite pore). The proteome of hydrogenosomes includes multiple paralogs of the Tom40-type of sequence. Although hydrogenosomes do not have an obvious VDAC, Tom40-type channels can serve as metabolite channels in the outer membrane [46], [47]; it is possible that one or more of these ‘Tom40’ sequences functions as a metabolite pore.

Hydrogenosomes also have four isoforms of a β-barrel protein, Hmp35/Hmp36. Neither BLAST searches nor structural homology detection engines detected Hmp35 or Hmp36 homologs in any other available genome sequence. The functions of Hmp35 and Hmp36 are not known, but Hmp35 possesses a cysteine motif, CX_6_CCX_2_CX_9_HX_15_CCXHXX_2_C, in its C-terminal region that could function to coordinate metal ions [48], and it is possible that Hmp35 might function in metal ion transport. The transported metal ions may include the iron that is essential for the function of hydrogenosomal FeS proteins [49] and that accumulates within these organelles [50]. Consistent with this idea, we observed increased expression of Hmp35 in *T. vaginalis* grown on iron-rich media, whereas the expression of Hmp36 was not affected (unpublished results).

The α-helical C-tail-anchored proteins are the second category of proteins found in the outer hydrogenosomal membrane. An outer membrane topology was confirmed for Tta1, which was selected from the 12 proteins with characteristics of C-tail-anchored proteins. In mitochondria, the C-tail-anchored proteins include important components of the outer mitochondrial membrane, such as Tom5, Tom6, Tom7, cytochrome b_5_, Fis1 and VAMP-1B [29], [31]. A single C-tail-anchored protein, VAP, was recently identified in the mitosomes of the related excavate, *Giardia intestinalis* [51]. The hydrogenosomal C-tail-anchored proteins revealed no homology with any known mitochondrial protein in other organisms. Surprisingly, given the topology of many mitochondrial proteins in fungi, animals and plants [16], [52], including the receptors of the TOM complex, Tom70 and Tom20, none of the hydrogenosomal membrane proteins that we identified contained N-terminal signal-anchor sequences.

### The inner membrane proteome in the hydrogenosomes of *T. vaginalis*

The inner mitochondrial membrane possesses a large variety of α-helical, single-spanning or polytopic proteins that are required for (i) protein transport across the inner membrane (TIM complex), (ii) the exchange of metabolites (MCF), and (iii) respiratory chain function. The hydrogenosomal inner membrane proteome seems to be less complex. We identified candidate core subunits of a TIM complex and the complete PAM machinery. However, all of the hydrogenosomal proteins that matched with the Tim17/22/23 family in the PFAM database were too divergent to allow the determination of their functional equivalents that correspond to any of the Tim17, Tim22 and Tim23 subfamilies, and their expression failed to support the growth of yeast strains lacking Tim17, Tim22 or Tim23. The hydrogenosomal MCF protein, Hmp31, was efficiently targeted into the inner membrane of *S. cerevisiae* mitochondria, and conversely, when yeast AAC was expressed in *T. vaginalis*, it was integrated into a hydrogenosomal membrane [21]. These data, together with the identification and characterization of the TvTim17/22/23 proteins, indicate that hydrogenosomes possess a functional equivalent of the TIM22 complex. However, it remains to be established whether the assembly of hydrogenosomal AACs is mediated by a specialized TIM22 complex as required for the assembly of metabolite carrier proteins in yeast, humans and (most likely) plants [52]. Another possibility is that *T. vaginalis* has a single inner membrane translocase for the assembly of membrane proteins and the translocation of matrix components. Such a situation would not be unprecedented, as a single gene coding for a Tim17/22/23 protein has been identified in the genomes of *Trypanosoma brucei* [15] and microsporidians [53], which strongly suggests the presence of a single multifunctional TIM complex in these protists.

The adaptation of hydrogenosomes to function in oxygen-poor or anaerobic environments is likely the major factor that resulted in the remarkable differences between the hydrogenosomal membrane machineries and the mitochondrial systems. These adaptations include the complete loss of inner membrane complexes of the respiratory chain, including the components generating a transmembrane electrochemical potential. In mitochondria, the membrane potential is essential to activate the Tim23 channel and exerts an electrophoretic effect on the positively charged N-terminal targeting signals of transported proteins. It is not clear how a membrane potential is generated in hydrogenosomes or whether the membrane potential is necessary for protein translocation into hydrogenosomes, although a requirement for a small potential has been suggested [25]. Such a fundamental difference in the character of the inner membrane might explain the observed divergence of hydrogenosomal TIM components, together with modifications of the corresponding substrates. Indeed, it has been demonstrated that hydrogenosomal targeting signals of matrix proteins possess a considerably lower negative charge than mitochondrial targeting presequences [26].

### Metabolite transport across the hydrogenosomal membranes

A remarkable difference was observed with respect to the limited spectrum of MCF proteins. Mitochondrial carriers are considered to be a unique eukaryotic invention that allows metabolic communication between the organelles and the cytosol [6]–[11]. A spectrum of MCFs that are specialized for the transport of various substrates is conserved across all eukaryotic groups, from the excavate, *T. brucei*, which has at least twenty-five carriers proteins [15], to humans, with forty-four MCF members [20]. Hydrogenosomal membranes have five MCFs that appear to be paralogs of Hmp31, which facilitates the transport of ADP and ATP across the inner hydrogenosomal membrane [21], [24]. The presence of only a single MCF-type AAC in the hydrogenosomal inner membrane most likely reflects the reduction of metabolic pathways, such as the pyruvate dehydrogenase-dependent conversion of pyruvate, the citric acid cycle, citrulline synthesis, and lipid breakdown, for which various MCF proteins are required [6]. However, there are several hydrogenosomal pathways that are dependent on substrate import or for which a metabolite exchange could be expected. The hydrogenosomal energetic metabolism is based on the import of pyruvate and malate, whereas acetate is released as a metabolic end product [1]. The hydrogenosomal localization of two components of the glycine cleavage system [54], arginine deiminase [55], and aminotransferase (this study) strongly suggests a requirement for amino acid exchange. Transporters for these substrates remain to be identified; however, they are unlikely to be of MCF types.

In addition to functional adaptations, some of the peculiarities in the hydrogenosomal membrane proteome might reflect the distant evolutionary history of *T. vaginalis* and other model organisms, such as *S. cerevisiae* and vertebrates (Figure 11). A case in point comes from the analysis of the TOM complex. The core TOM complex components have been found in all eukaryotic lineages, suggesting their presence in a common mitochondrial ancestor [14]. However, the phylogenetic distribution of the additional components that optimize the function of the TOM complex is lineage-specific. In *S. cerevisiae*, the TOM complex comprises seven components (Tom70, Tom40, Tom22, Tom20, Tom7, Tom6, and Tom5) [16]. In *T. vaginalis*, we did not identify Tom20 or Tom70, which function as receptors for N-terminal and inner targeting signals, although both types of signals are conserved in this organism [25], [27]. The absence of these components seems to be common to the eukaryotic group, Excavata [56], to which trichomonads belong, as they have not been identified in the related protists, *Giardia intestinalis*, *Trypanosoma brucei* or *Naegleria gruberii* [51], [57] (unpublished results), or in plants [14], [58], [59]. Although there may be a large number of membrane proteins (58%) in the hydrogenosomes of *T. vaginalis* without known functional domains, whether these components represent highly divergent homologs of known systems that were not recognized or represent novel systems will require further functional investigations.

## Materials and Methods

### Cell cultivation

*T. vaginalis* strain T1 (kindly provided by J.-H. Tai. Institute of Biomedical Sciences, Taipei, Taiwan) was grown in Diamond's Tryptone-Yeast extract-Maltose medium (TYM) with 10% (v/v) heat-inactivated horse serum at 37°C.

### Extraction of membrane proteins in Triton X-114

Highly purified hydrogenosomes were obtained from cell lysates by differential and Percoll-gradient centrifugation [60]. Membrane proteins were isolated using Triton X-114 as previously described [61]. Briefly, hydrogenosomes were solubilized for 1 hour on ice with 2% (w/v) Triton® X-114 (Sigma) in Tris buffer (150 mM NaCl, 10 mM Tris·HCl and 1 mM EDTA, pH 7.4) at a ratio of 1 mg of hydrogenosomal pellet to 4 ml of the buffer. Solubilized hydrogenosomes were centrifuged at 20,00× g for 20 minutes (min) at 4°C. The pellet was discarded, and the supernatant was incubated for 3 min at 37°C. The Triton X-114 phase was separated from the aqueous phase by centrifugation for 1 min at 13,000× g. The aqueous supernatant with soluble proteins was discarded, and the Triton X-114 phase containing the membrane proteins was redissolved in 10 volumes of Tris buffer at 4°C. The ensuing extraction of the Triton-X114 phase at 37°C and solubilization in ice-cold Tris buffer was repeated twice. Proteins in the final Triton X-114 phase were precipitated by the addition of 10 volumes of ice-cold acetone and air-dried.

### SDS-PAGE and liquid chromatography

Proteins were separated by 1D SDS PAGE and digested in-gel (slices) by trypsin; subsequently, the tryptic peptides were separated by reverse-phase nano liquid chromatography. The Triton X-114-extracted proteins were solubilized in sample buffer (20% glycerol, 4% SDS, 0.02% bromophenol blue and 125 mM Tris·HCL, pH 7.4) and separated on a 12% gel by SDS PAGE. The gel was stained with Coomassie Brilliant Blue R-250, and the lanes with separated proteins were cut to 1-mm wide slices. Each slice was transferred to a separate microtube, and the proteins were subjected to in-gel tryptic digestion using sequencing-grade modified trypsin (Promega) as described previously [62]. The tryptic peptides were separated by liquid chromatography using an Ultimate 3000 HPLC system (Dionex). The peptide samples, diluted in 0.3% TCA with 10% ACN, were loaded onto a PepMap 100 C18 RP column (3-µm particle size, 15-cm length, 75-µm internal diameter; Dionex) at a flow rate of 300 nl per minute. The peptides were eluted by a 45-min linear gradient of 5–80% (v/v) ACN in 0.1% (v/v) TCA over a period of 45 min. The eluate (60 nl) was mixed 1∶3 with matrix solution (20 mg/ml a-cyano-4-hydroxycinnamic acid in 80% ACN) prior to spotting onto MALDI target plates using a Probot microfraction collector (Dionex).

### Mass spectrometry and MS/MS data analysis

Spectra were acquired using a 4800 Plus MALDI TOF/TOF analyzer (Applied Biosystems/MDS Sciex) equipped with an Nd:YAG laser (355 nm) with a firing rate of 200 Hz. All of the spots were measured in the MS mode, and up to 10 of the strongest precursors were selected for MS/MS, which was performed with a collision energy of 1 kV and an operating pressure of collision cell set to 10^−6^ Torr. Peak lists from the MS/MS spectra were generated using GPS Explorer v. 3.6 (Applied Biosystems/MDS Sciex) and searched by local Mascot v. 2.1 (Matrix Science) against annotated proteins in the TrichDB database (http://trichdb.org/trichdb/). Database search criteria were as follows: enzyme - trypsin; taxonomy - *Trichomonas vaginalis*; fixed modification - carbamidomethylation; variable modification - methionine oxidation; peptide tolerance −120 ppm, allowing one missed cleavage; and MS/MS tolerance −0.2 Da.

### Protein sequence analysis

All of the identified protein sequences were manually annotated based on searches in the TrichDB (http://trichdb.org/trichdb/), Uniprot Protein knowledgebase (http://www.uniprot.org/), NCBI (http://blast.ncbi.nlm.nih.gov/Blast.cgi) and Pfam (http://pfam.sanger.ac.uk/search) databases. The protein sequences (<1000 residues) were submitted (*i*) against a 90% redundancy-reduced NCBI nr database by means of simple pair-wise alignment Psi-BLAST for 8 iterations at an e-value cutoff of 10^−3^ and (*ii*) against the Pfam 23.0 A+B database of families represented by multiple sequence alignments and hidden Markov models (HMMs) at an e-value of 0.044.

TargetP and SignalP, based on the combination of artificial neural networks and hidden Markov models, respectively (http://www.cbs.dtu.dk/services/), together with PsortII (http://psort.ims.u-tokyo.ac.jp/), were used to predict the subcellular location.

An application based on the NetBeans Platform (Hunter) was used to predict the subcellular localization of proteins according to their N-terminal amino acid sequence, as previously described [26]. The following parameters were used: (i) the presequence start motif - ML[ACGQRSTV] or MS[ILV] or MIS or MTL; (ii) a cleavage site motif - R.F[TKILFSAGQ] or R[AFNESG][TYILFSAGQ] or K[AFNESG][TYILFSAGQ] or K.F[TKILFSAGQ]. The secondary structure and topology of alpha-helix integral membrane proteins was predicted using two bioinformatic tools: TMHMM (http://www.cbs.dtu.dk/services/) and Memsat3 (http://bioinf.cs.ucl.ac.uk/memsat/).To determine members of protein families, a hidden Markov model analysis was performed according to the method of Likic et al. [63].

### Selectable transformation of *T. vaginalis* and immunofluorescence microscopy

Selected genes were amplified by PCR from *T. vaginalis* genomic DNA and inserted into the TagVag2 plasmid. Cells were transformed and selected as described previously [64]. *T. vaginalis* cells expressing recombinant proteins with a C-terminal hemagglutinin (HA) tag were identified with a mouse anti-HA mAb [60]. The malic enzyme was detected with a rabbit anti-malic enzyme polyclonal antibody [65]. A secondary AlexaFluor-488 (green) donkey anti-mouse antibody and AlexaFluor-594 (red) donkey anti-rabbit antibody were used for the visualization of the target proteins. Cells were observed using an OLYMPUS Cell-R, IX81 microscope system, and images were processed using ImageJ 1.41e software (http://rsb.info.nih.gov/ij/).

### Structural modeling

The model of TvTim44 (residues 144–326) was built using the human Tim44 structure (PDB ID: 2cw9) [66] as a template. The alignment was constructed by MUSCLE [67] and manually edited. The 3D structure model of TvTim 44 was built using Modeller 9v7 [68]. The quality of the final model was checked using What Check [69] and ProSA-web services [70]. The electrostatic potential on the solvent-accessible surface of TvTim44 was calculated using APBS 1.3 [71].

### Sequential proteolytic degradation, protein extraction and protein import into *Saccharomyces cerevisiae* mitochondria

Mutant yeast strains and their corresponding wild-type control strains were grown in parallel in YPLac medium at 30°C. The mitochondria were isolated by differential centrifugation, and protein import assays were performed as previously described [72]. BN PAGE analysis following the import of the precursor proteins was performed according to a previously described method [72]. Sequential proteolytic degradation following the import of radiolabeled precursors and protein extraction was performed as described previously [73].

### Functional complementation

Functional complementation of yeast mutants was performed by transfection of a heterologous-protein encoding plasmid into Δ*pam18/*Pam18, Δ*tim17/*Tim17, Δ*tim22/*Tim22, or Δ*tim23/*Tim23 cells. To cause the yeast to sporulate, the cells were grown in rich media at 30°C to mid-log phase, isolated by centrifugation, and then transferred to a 1% potassium acetate solution and incubated with shaking at 25°C for 3 to 4 days. The resulting tetrads were dissected onto YPD plates and incubated at 30°C.

### Growth and viability analysis

Yeast cells with a disrupted *pam18* gene and carrying the pScPam18 or pScNTvJPam18 construct were grown to a mid-logarithmic growth phase (0.6< OD_600_<0.8) in rich media and diluted to an OD_600_ of 0.2. Each cell suspension was then further diluted in 7 five-fold steps in sterile double-distilled water, and 5 µl of the last 6 diluted cell suspensions was spotted onto media plates. The plates were incubated at 25°C, 30°C or 37°C until colonies were visible.

### Sequential proteolytic degradation of C-tail anchored proteins targeted to hydrogenosomes

Aliquots of Percoll-purified intact hydrogenosomes (3 mg) were resuspended in 1 ml of ST buffer (250 mM sucrose, 10 mM Tris, pH 7.4, 0.5 mM KCl, 50 µg/ml TLCK and 10 µg/ml leupeptin). Trypsin was added to final concentrations of 50–200 µg/ml, and the samples were incubated on ice or in a water bath at 37°C for 30 min. After incubation, soybean trypsin inhibitor was added (5 mg/ml), and the samples were analyzed by immunoblotting using a monoclonal mouse anti-HA antibody and an anti-pyruvate:ferredoxin oxidoreductase antibody (kindly provided by Patricia Johnson, UCLA, USA and Guy Brugerolle, University of Clermont Ferrand, France, respectively).

### Gel size analysis of Tom40

Approximately 30 mg of Percoll-purified hydrogenosomes were solubilized in 0.5 ml of ice-cold gel size running buffer (150 mM NaCl, Tris [pH 8.0], 0.5 mM MgCl_2_, 5% ethylene glycol, 50 µg/ml TLCK and 10 µg/ml leupeptin) with 0.5% Triton X-100 on ice for 1 hour. Solubilized hydrogenosomes were centrifuged at 13,000× g for 10 min. The supernatant was loaded onto a Superdex 200 10/300 GL column (GE healthcare; equilibrated with Gel Filtration Standards©, BioRad) and separated using a flow rate of 0.5 ml/min. Fractions (500 µl) were collected, and the proteins were extracted using TCA-methanol/chloroform and analyzed by immunoblotting using a monoclonal mouse anti-HA antibody.

References for the supporting information are available as a separate document in References S1.

## Supporting Information

Figure S1 **Mitochondrial carrier proteins in** ***Trichomonas vaginalis*** **.** Sequence alignment of *Trichomonas vaginalis* mitochondrial carrier homologs ADP/ATP carrier 1 (Hmp31, TVAG_237680) AAC-2 (TVAG_051820), AAC-3 (TVAG_164560), AAC-4 (TVAG_197670) and AAC-5 (TVAG_262210) and bovine mitochondrial AAC (NP_777083). Solid lines above the alignment represent alpha-helixes, as deduced from the structure of the bovine AAC [1]. The signature motif of the AAC protein family is shown in box.
(TIF)

Figure S2 **The C-terminal domains of putative C-tailed anchored proteins identified in** ***T. vaginalis*** **hydrogenosomal membranes.** Predicted transmembrane domains (TMD) are highlighted in yellow. Positively charged residues are in red, and negatively charged residues are in green. AA TMD indicates the number of amino acids in the TMD.
(DOC)

Figure S3 **Protein sequence alignment of candidate porin_3 family proteins in hydrogenosomal membranes.** The secondary structure of all of the protein sequences, as predicted by PSIPRED, is shown above the protein alignment. Predicted beta-strands are in green, and predicted alpha helixes are in purple. The presence of the beta-signal is highlighted in red.
(PDF)

Figure S4 **Sequence alignment of** ***Trichomonas vaginalis*** **β-barrel proteins, Hmp35 and Hmp36.** Cysteines and histidines of the putative metal binding motif, CX_6_CCX_2_CX_9_HX_15_CCXHXX_2_ C, are highlighted in yellow. Hmp35-1 (TVAG_590550), Hmp35-2 (TVAG_104250), Hmp36-1 (TVAG_031860) and Hmp36-2 (TVAG_216170).
(PDF)

Figure S5 ***Trichomonas vaginalis*** **TvTim17-22-23A and TvTim17-22-23B cannot substitute for Tim17, Tim22 and Tim23 in** ***Saccharomyces cerevisiae*** **.** Yeast Tim17/Δ*tim17*, Tim22/Δ*tim22* or Tim23/Δ*tim23* cells were transformed with plasmids carrying TvTim17-22-23A (A), TvTim17-22-23B (B) or TvTim17-22-23A, respectively, where key residues were mutated to restore the PRAT motif (T97Y D112K) (C). Cells were sporulated, and the tetrads were dissected onto YPD plates. Two viable colonies indicate no complementation by the candidate protein, whereas four viable colonies indicate successful complementation by the candidate sequence.
(PDF)

Figure S6 **Alignment of** ***T. vaginalis*** **Pam16 and Pam18 against eukaryotic orthologs.** The diagnostic features identified in TvPam18 are (i) a J-domain at the C-terminus of the protein with a typical arrangement of three helixes and a short linker with a conserved HPDXGGS sequence motif connecting helixes II and III. The invariant HPD tripeptide is critical for the stimulation of the ATPase activity of Hsp70 by Pam18. (ii) A transmembrane α-helix that is close to the N-terminus. TvPam18 also contains a short N-terminal extension that is predicted to be a targeting presequence and a short N-terminal intermembrane space domain. However, TvPam18 does not contain a conserved interaction arm in front of helix I, which was structurally defined by [2] as one of the means by which Pam18 interacts with Pam16. The Pam16 protein family contains a degenerate J-domain with homology to Pam18 that lacks the HPD tripeptide in the linker motif; thus, it is unable to stimulate the ATPase activity of Hsp70. In TvPAM16, HPD is replaced by a D90, L91, E92 tripeptide, whereas the GGS motif of the linker is conserved. Importantly, TvPam16 contains a conserved L99 in the J-like domain that corresponds to L97 in the yeast ortholog. This residue has been shown to mediate an essential interaction between the Pam16 J-like domain and the J domain of Pam 18 that stabilizes the heterodimer [3]. A second characteristic feature predicted in TvPam16 is an N-terminal hydrophobic domain that is required for the interaction of Pam18:Pam16 heterodimer with the TIM23 translocon. The J-like domain of TvPam16 is underlined. Helical structures of the J-like domain (in red) were predicted by PSIPRED (http://bioinf.cs.ucl.ac.uk/psipred/). Arrowheads indicate the degenerate HPD motif of Pam16 between helixes II and III. The HPD motif of Pam18 is boxed. The leucine and asparagine residues essential for the Pam16-Pam18 interaction are marked with a star. Organisms and accession numbers: *T. vaginalis*, TVAG_470110; *S. cerevisiae*, Pam16, P42949; *H. sapiens*, Pam16, Q9Y3D7; *D. discoideum*, Pam16, XP_640279; *E. cuniculi*, Pam16, ECU11_0700; *T. vaginalis*, Pam18, TVAG_436580; *S. cerevisiae*, Pam18, Q07914; *H. sapiens*, NP_660304.
(PDF)

Figure S7 **Alignment of the Tim44 domain of** ***T. vaginalis*** **against eukaryotic and bacterial orthologs.** Conserved hydrophobic residues that form the large hydrophobic pocket of Tim44 are highlighted in yellow [4], [5]. The conserved proline mutation, which causes familial oncocytic thyroid carcinoma, is in red [6]. Organisms and accession numbers: *Saccharomyces cerevisiae*, Q01852; *Schizosaccharomyces pombe*, NP_595905; *Phytophora infestans*, XP_002997475; *Tribolium castaneum*, XP_975336; *Homo sapiens*, NP_006342; *Caenorhabiditis elegans*, O02161; *Caulobacter crestentus*, AAK25703.
(PDF)

Figure S8 **Tim44 is a peripheral membrane protein exposed at the matrix side of the inner membrane that provides a molecular scaffold for the assembly of the import motor** [**7**] **.** The BLAST algorithm (NCBI BLAST, reference) using the PDB database of macromolecular structures detected a sequence similarity between the C-terminal part of TVAG_008790 and the C-terminal part of yeast Tim44 (E = 6^−4^). This result was further supported by recognition of the C-terminal Tim44 domain by PFAM (E = 7^−3^) and HHsenser (E = 1^−4^) (Table S2). The structure of the C-terminal part of human Tim44 [8] was used to build the model of the C-terminal part of TVAG_008790 (residues 144-326). The resulting structure shows that all of the secondary structures present in human Tim44 appear in TvTim44 (Fig. 8B). The C-terminus of TvTim44 can form a large characteristic pocket with the conserved hydrophobic residues (Fig. 8C and Fig. S7 alignment) that were suggested to participate in the binding of Tim44 to the inner membrane [8]. A significant difference can be observed at position 225 of TvTim44, where an Arg replaces a hydrophobic Leu or Phe in orthologous species (Fig. S7 alignment). The positively charged domain of human Tim44 implicated in the binding of cardiolipins (residues 289-295) is not well conserved in TvTim44, although calculations of the electrostatic potential of TvTim44 also suggest a positive charge in this area (Fig. 8D charge identification). The low conservation of this domain likely reflects an absence of cardiolipin in *Trichomonas vaginalis* [9].
(PDF)

Figure S9 **ScNTvJPam18 can support wild-type rates of cell viability and** ***in vitro*** **protein import.** (A) Equal cell numbers of wild-type or complemented yeast were serially diluted onto medium containing glucose or lactic acid as a carbon source and incubated at 25°C, 30°C or 37°C. (B) Mitochondria from wild-type and complemented cells were isolated and incubated at 25°C with [^35^S]-labeled precursors for the indicated time, treated with 25 µg/ml proteinase K to degrade the surface-associated proteins, and analyzed by SDS-PAGE and digital autoradiography.
(PDF)

References S1 **References for the supporting information figures and tables.**
(DOC)

Table S1 **Complete list of proteins identified by nanoLC MS/MS in four independent experiments.**
(DOC)

Table S2 **Identification of hydrogenosomal proteins using TrichDB** (http://trichdb.org/trichdb/), Uniprot (http://www.uniprot.org/), and PFAM A+B (http://pfam.sanger.ac.uk/) searches.
(DOC)

Table S3 **Putative matrix proteins identified in** ***T. vaginalis*** **hydrogenosome.**
(DOC)

Table S4 **List of putative non-hydrogenosomal proteins.**
(DOC)
