# Supplementary figures and images for: The Core Components of Organelle Biogenesis and Membrane Transport in the Hydrogenosomes of Trichomonas vaginalis
Source: PLoS One. 2011 Sep 15;6(9):e24428. doi: 10.1371/journal.pone.0024428 (PMC3174187; doi:10.1371/journal.pone.0024428)

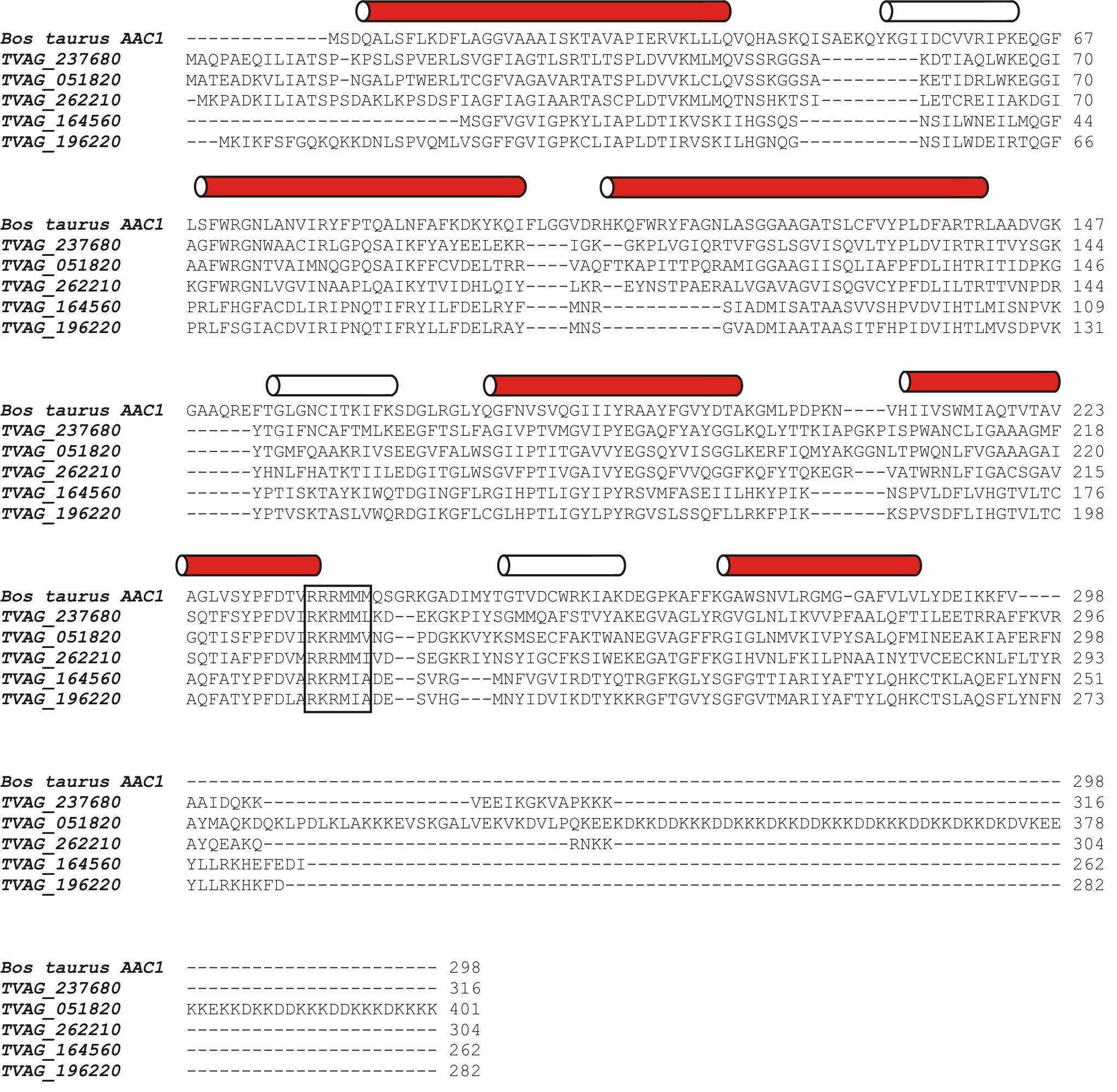

Supplement: Figure S1 — Mitochondrial carrier proteins in Trichomonas vaginalis . Sequence alignment of Trichomonas vaginalis mitochondrial carrier homologs ADP/ATP carrier 1 (Hmp31, TVAG_237680) AAC-2 (TVAG_051820), AAC-3 (TVAG_164560), AAC-4 (TVAG_197670) and AAC-5 (TVAG_262210) and bovine mitochondrial AAC (NP_777083). Solid lines above the alignment represent alpha-helixes, as deduced from the structure of the bovine AAC [1]. The signature motif of the AAC protein family is shown in box. (TIF) [file pone.0024428.s001.tif]

A

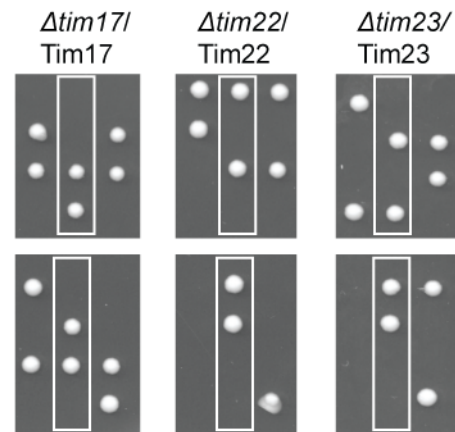

B

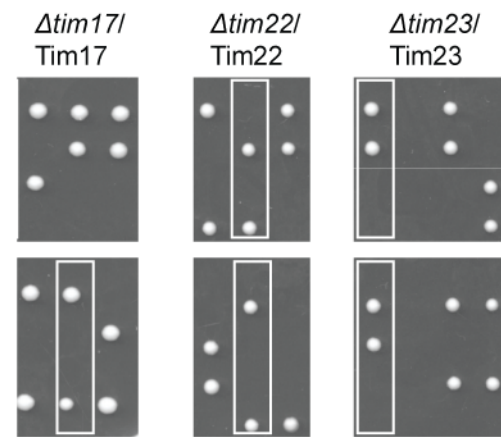

C

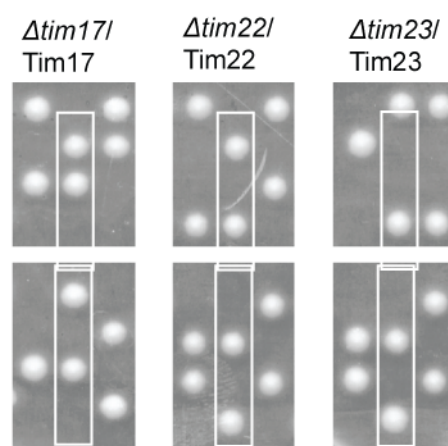

Supplement: Figure S5 — Trichomonas vaginalis TvTim17-22-23A and TvTim17-22-23B cannot substitute for Tim17, Tim22 and Tim23 in Saccharomyces cerevisiae . Yeast Tim17/Δtim17, Tim22/Δtim22 or Tim23/Δtim23 cells were transformed with plasmids carrying TvTim17-22-23A (A), TvTim17-22-23B (B) or TvTim17-22-23A, respectively, where key residues were mutated to restore the PRAT motif (T97Y D112K) (C). Cells were sporulated, and the tetrads were dissected onto YPD plates. Two viable colonies indicate no complementation by the candidate protein, whereas four viable colonies indicate successful complementation by the candidate sequence. (PDF) [file pone.0024428.s005.pdf]

A

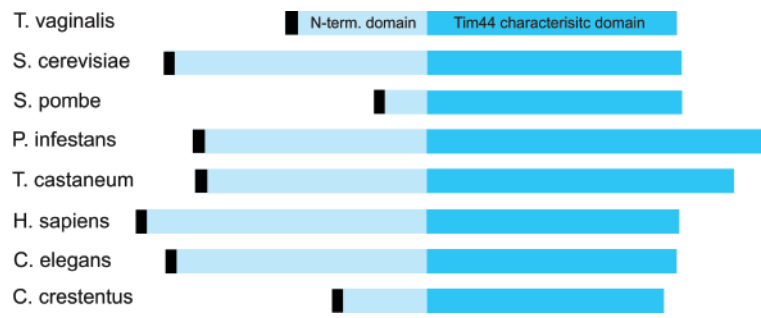

B

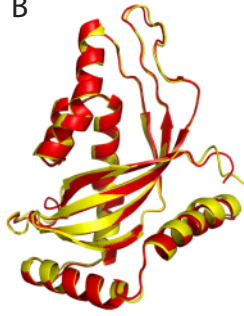

C

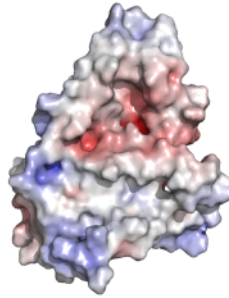

D

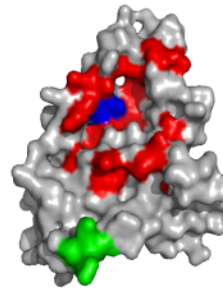

Supplement: Figure S8 — Tim44 is a peripheral membrane protein exposed at the matrix side of the inner membrane that provides a molecular scaffold for the assembly of the import motor [7] . The BLAST algorithm (NCBI BLAST, reference) using the PDB database of macromolecular structures detected a sequence similarity between the C-terminal part of TVAG_008790 and the C-terminal part of yeast Tim44 (E = 6−4). This result was further supported by recognition of the C-terminal Tim44 domain by PFAM (E = 7−3) and HHsenser (E = 1−4) (Table S2). The structure of the C-terminal part of human Tim44 [8] was used to build the model of the C-terminal part of TVAG_008790 (residues 144-326). The resulting structure shows that all of the secondary structures present in human Tim44 appear in TvTim44 (Fig. 8B). The C-terminus of TvTim44 can form a large characteristic pocket with the conserved hydrophobic residues (Fig. 8C and Fig. S7 alignment) that were suggested to participate in the binding of Tim44 to the inner membrane [8]. A significant difference can be observed at position 225 of TvTim44, where an Arg replaces a hydrophobic Leu or Phe in orthologous species (Fig. S7 alignment). The positively charged domain of human Tim44 implicated in the binding of cardiolipins (residues 289-295) is not well conserved in TvTim44, although calculations of the electrostatic potential of TvTim44 also suggest a positive charge in this area (Fig. 8D charge identification). The low conservation of this domain likely reflects an absence of cardiolipin in Trichomonas vaginalis [9]. (PDF) [file pone.0024428.s008.pdf]

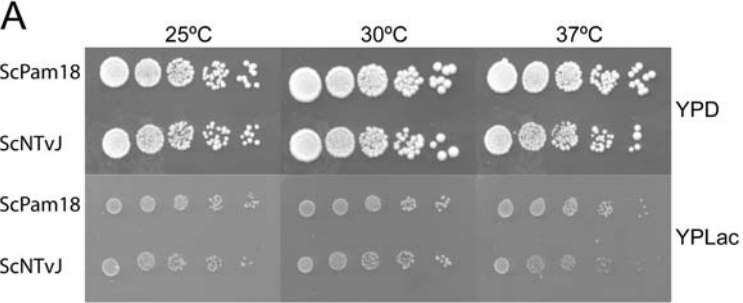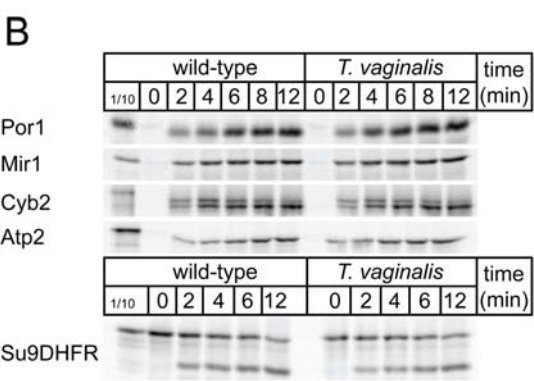

Supplement: Figure S9 — ScNTvJPam18 can support wild-type rates of cell viability and in vitro protein import. (A) Equal cell numbers of wild-type or complemented yeast were serially diluted onto medium containing glucose or lactic acid as a carbon source and incubated at 25°C, 30°C or 37°C. (B) Mitochondria from wild-type and complemented cells were isolated and incubated at 25°C with [35S]-labeled precursors for the indicated time, treated with 25 µg/ml proteinase K to degrade the surface-associated proteins, and analyzed by SDS-PAGE and digital autoradiography. (PDF) [file pone.0024428.s009.pdf]
