# Supplementary material for: The Core Components of Organelle Biogenesis and Membrane Transport in the Hydrogenosomes of Trichomonas vaginalis
Source: PLoS One. 2011 Sep 15;6(9):e24428. doi: 10.1371/journal.pone.0024428 (PMC3174187; doi:10.1371/journal.pone.0024428)
Supplement: Figure S2 — The C-terminal domains of putative C-tailed anchored proteins identified in T. vaginalis hydrogenosomal membranes. Predicted transmembrane domains (TMD) are highlighted in yellow. Positively charged residues are in red, and negatively charged residues are in green. AA TMD indicates the number of amino acids in the TMD. (DOC) [file pone.0024428.s002.doc]

**Accession**

**Number TMD AA TMD**

TVAG_190830 -EDRSYAGIGKIIAIGGGIAAFVAGFSYAIYKKFSRK-COOH 20

TVAG_272350 -YGEPKPTDWKFIGTMVAIGVGAGLATHWLIKLITKVAKKKE-COOH 20

TVAG_137270 -PDDEYPTINTKAMILTGIAGAAVLVGAIIIIAKKLMKK-COOH 20

TVAG_277930 -HGIPFNLSKVGISKPLIVGGAVIAAGFLLYKGIKRFMNHK-COOH 23

TVAG_369980 -NEQNQEKLNSFYNKFWGIFSVVAFFGVIIFCKDEKK-COOH 21

TVAG_393390 -RKQQKQKKVLIIVLAVMAVVTVCVAVLVIILWLRK-COOH 23

TVAG_211970 -IRKKLVSSNISTYVIAGTAVLAASAAAFLFFSRRK-COOH 23

TVAG_174010 -AKAKPQTNTSFSTIIGLGITVGVIVGVVVALYKKFKHH-COOH 23

TVAG_240680 -DSSRKFKKTVLPYLIIAIVVVVIGVAVGLGFYFYKKGSLDSILPNSRLV-COOH 23

TVAG_458060 -KGVSTGIKASAAVMAGLAIAGFAALAFSYMKRRK-COOH 20

TVAG_090120 -EGKDDSFNMNDLYLILGIGAAIGVGAFCFYKIWKRLQRK-COOH 23

TVAG_283120 -DEKETTSTYKIITAVAVSVSICAAAYFLFRKKKLPEIKTAKK-COOH 19

Tom5 NP_015459 -QEQTEKTLKQAAYVAAFLWVSPMIWHLVKKQWK-COOH 18

VAMP1B NP_058439 -KRKYWWKNCKMMIMLGAICAIIVVVIVRRD-COOH 17
