# Supplementary material for: The Core Components of Organelle Biogenesis and Membrane Transport in the Hydrogenosomes of Trichomonas vaginalis
Source: PLoS One. 2011 Sep 15;6(9):e24428. doi: 10.1371/journal.pone.0024428 (PMC3174187; doi:10.1371/journal.pone.0024428)
Supplement: Figure S3 — Protein sequence alignment of candidate porin_3 family proteins in hydrogenosomal membranes. The secondary structure of all of the protein sequences, as predicted by PSIPRED, is shown above the protein alignment. Predicted beta-strands are in green, and predicted alpha helixes are in purple. The presence of the beta-signal is highlighted in red. (PDF) [file pone.0024428.s003.pdf]

```
      CCCCCCCCCCCCCCCCCCCCCCCCCEEEECCCCCC     CEEEEEEEEEECCEEEEEECCCCCCCCC
       CCCCCCCCCCCCCEEEECCCCCCEEEEECCCCCCC    CEEEEEEEEECCCEEEEEECCCCCCEEE
        CCCCCHHHHHHCCCCCCCCCCEEEECCCCCCC     CCEEEEEEEEECCCEEEEEECCCCCCEEC
         CCCCCCCCCCEEEECCCCCCCCCCCCEEEEECCCCCCEEEECC CCEE    EEEEECCCCCCCEEEEEEEECCCEEEEE
                 CCCCCCCCCCCHHHHHHHCCCCCCCCC CCCCEEEEEEEEE    CCCCCCEEEEEEECCCCCCHHHHHHCCC
           CCCCCCCCCCCCCCCCCCCHHHHCCEECCCCCCC CCCCEEEEEEEEC    CCCCCCEEEEEEECCCCCHHHHHHHCCC
                       CCCCEEHHHHCCCCCCCCCCCEEEECCCCCCCCEEEEECCCCCCCCCEEEEEEEEECCCEEEEEEEC
                       CHHHHHCCCCCCCCCCCCCCCCEEECCCCCCCCC      EEEEEEEECCCCEEEEEECCCCCCCCC
Tom40-1  ------------MILESFSQVTSRDNAILELPYYPGLKLEASYNLGN-----DAYFHSYVSFRPLKISIVPGPNGQYNSI 63
Tom40-3  -------------MLSSFLHTNLRHQEIFDVGAFSGLNITFDSPTQSK----DTTISTVIFLQPLSISIVRGEGGKPETR 63
Tom40-2  --------------MKSGTEYYESMNNLFDNKNFPGIKFDVELNQSP-----ETTINSSINIRHPYFNIIEKNSQTKIVP 61
Tom40-4  MSEPEPKTEQTVTLLPQYEDINAENDVMFTLNDTKGLSLKLGG-KGKF----DVNYSIGGALRPTKVAIVPQVNGEFSIL 75
Tom40-5  --------MIFDPKQTKCPEFEAFESEIVGPTSLP-PNNTFEIVVAKK----LKDIGNLISVLSIKPRLYPLKYADQAGE 67
Tom40-6  --MGALQSTPEQKAPELVGDFDTLLGKVTDVASLN-LINGFEFTHVYQ----PQPSHSIVQTISISPRIHPVELAAQAGE 73
Porin-1  --------------MKQLKVILAQTKKLFNIPIQNKLCFNAPVMNNNFSLQLEKPSQKDVQMCVTTNLKFKNGLIVATAH 66
Porin-2  --------------MWERLAHHLEDSRLHLRPNIPSITFKKEADFLQG------RFTVNYPLSFNAELFVSSKGFKDASF 60


         CCCCEEEEEEEEEECCCCEEEEECCCCCEEEEEEEECCCCCEEEEEEEECCCCCCCEEEECCCCCEEEEEEEEECCCCEE
         ECCEEEEEEEEEECCCCCCEEEECC CCCEEEEEEECCCCCCEEEEEEECCCCCCCCCCCCCCCCEEEEEEEEEECCCEE
         CCCCEEEEEEEEECCCCCCEEEECCCCEEEEEEECCCCCCCEEEEEEECCCCCCCCCEECCCCCCEEEEEEEEEEC
         ECHHHHHHHHHHHCCCCCEEEECCCCCCCCCCCEECCCCCCCCCCCCCCHHHHHHHHHHHHHCCCCCCCEEEEECC
         EECCCCEEEEEEECCCCCEEEEEECCCCCEEEEEEECCCCCCEEEEEEEECCEEEEEEEEECCCCCCEEEEEEEEH
         EECCCCEEEEEEEEECCEEEEEECCCCCCCCEEEEEEECCCEEEEEEEEEECCCEEEEEEECCCCCCCHHHHCCC
         CCCCEEEEEEECCCCCCCEEEEECCCCCCCEEEEEEEEEEEEECEEECCCEEEEEECCCCCCCCCEEEEEECCEEEC
         CCCCCCCCCEEEEECCCCEEEEEEECCCCCCCCCHHHHCCEEEEEECCCCCCCEEEEEEEEECCEEE
Tom40-1  QNQTAVAYTTFKKTINKNIYLLTLGNQNSSFSLYFKQIPKVNIMSRFQIGDGMLSPAIKFSYLGKYIHPVIQISGAAQKL 143
Tom40-3  SNQTTNVKFNYQKTNEKGSIIMNLG-TSQFFFVNIKPIPNIGFSAQASVNRGSVCPSIGTNLVGKHFYFRSMLTMVGQKL 142
Tom40-2  SPIRIAQVVLNHQTSKLSNVILSLGQKNYISYQKSNSNKNTSVRCRCDFTSTSFIPSILLENNSKYHNFSIQAQTR---- 137
Tom40-4  SLPIRSLYLSLLKSFPKCSITSTLSSDMVDLSAIVNPTPFAIGKAGFLTNFQMWNAYAFAIFRQPWSNYCVSFSTS---- 151
Tom40-5  QRLPGNAAVNFTTDKIGFPISIDVSRDSPILFSATMPIIDSLNLAVSTTLEKKMKMSFTLNHFNPYTNLSFNYSVA---- 143
Tom40-6  KVIPGNYSAVFVGTVSDMEYRVALPLFGSPNVQLTVPLSQYSKTSLYVDCTLNPGITGYLKYRGPKVASEFAFSAS---- 149
Porin-1  SDNPCSILFQTNAIYPGVKFTTNISSNEKNTIISKVSYCTIYKNMIFTPKVKVILQDKPYNYLNTDVHFKTNVNYYG--- 143
Porin-2   HPSFSPNVPFYLNVTSDKTYTAAFNFKLPNEKLPEKLRNNTFKFSLSGDQSNYLSSVFALTHQNKYS------------ 127


         EEEECCCCCEEEEEEEEEEEEEEECCC CCCCHHHHHHHHHHHCC   EEEEEEEEEECCCCEEEEEEEECCCCCHHHHH
         EEEECCCCCCCCEEEEEEEEEEEEECC CCEEEHHHHHHHHCCCCCCEEEEEEEEHHHCCCCCCCCHHHHCCCCHHHHHE
                    CEEEEEEEEEEEECCC CCEEEHHHHHHHHCCCC    CEEEEEEEECCCCCCEEEEEECCCCCCCEEE
                    CCCCCCCCCCCCCCCC CCCCEEEEECCCC       CCCCEEEEEEECCCCEEEEECCC CCCHHHH
                    HHHHHHHCCEEECCCCCCCEEEECCCEEECCCCC    EEEEEEEEECCCCCCEEEEECCCCEEEEEE
                    CEEECCCCCCHHHHHH CCCCCEEEEECCCCCCC   EEEEEEEECCCCCCCCEE EEECCCCEEEEEE
                    CHHHHHHHHHHHCCCCHHHHHHCCCEEEEEEECCEEEECEEEEEEEECCCCEEEEEEEEECC   CEEEEE
                       EEEEEEEEEEEEEECCCCCEEEEEEECCCCC       CCHHHHHHCCCCCCHHHHHHHHHCCCEE
Tom40-1  IDVQANDFKFYNFSLVNSMQISTTIGR-PDLMLGVQYIKSLRQQG---VYSLSTFVRNENEKRVLSLSFVKEDVSFVALQ 219
Tom40-3  ISVTDNKFNFFDVNRISTIDTSINLGR-NDFSAGLQIVRRFLGENSRWAYSVLVQKEWDNNNSAFDIAAVADPLFNLCLR 221
Tom40-2  -----------DMIYIALIDTKMTLRY-KNFIFTYQNVRNCVKGN----TAYSILLHYKHGLRDFQAAIMKDNFRTLTLR 201
Tom40-4  -----------ELKKPELQSASAVIGH-PNINFGIQYTPQD--------PAAKLSLMCYANIKSYKFHMASN-IMNYKAR 210
Tom40-5  -----------DSFKTALYNFKIASGVPKKFCVGFNGAISSKGNT-----EYSLIGSIKANQLNLSTILSKSQNLTISFV 207
Tom40-6  -----------NYFTQGNIIANFSTMY-KKTLFGAIIQKPLFSAG----YVFRAGCATDFGKFNDGIFLSLEPKLRINKS 213
Porin-1  -----------CGFTALFKKLEQSFTLIPYIRKFGHHAISLKFKNYKVVPEIYTIFPYEDFQFAFHTFYPTR---SLSFY 209
Porin-2  --------------ISLALRPCIIGGFYHGENVGVEFAYSLAEEQ-------PSMSAMFNKRFDRTEVSLMASMYGEGTA 186


         HHHHCC CEEEEEEEEEECC CCCEEEEEEEEEECC   EEEEECCCCC EEEEEEEEECCCCEEEEEEECCCCCCC
         EEEECCCCCEEEEEEEECCC CCCCEEEEEEEECCC   CEEEEEEECCCEEEEEEECCCCCCEEEEEEEECCCCCC
         EEECCCCCEEEEEEEEECCC CCCCEEEEEEEECCC   EEEEEECCCCEEEEEEEEEEECCCEEEEEEEEECCCCC
         HHHCCCCCCEEEEEEEECCCCCEEECCCCEEEECCC   CEEEEEEECCCCHHHHHHHHHHHCEEEECCCCCCCCCC
         EEECCCCCCEEEEEEEECCCEEEEEEEEEEHHHCC   CEEEEEEECCCCEEEEEEEEECCCCEEEEEEEHHHHHHH
         EEECCCCCCCCCEEEEEEECCHHHCCHHHHHHHHHHH   EEEEEEEEECCCCCHHHHHHHCCCCEEEEEEEHHHHHHH
         EEECCCCCCCCCCCEEEEECCCCCCEEEEEECCCCCCCCCCEEEEEECCCCCEEEECCCCCCEEEEEEEEEEEEECCCCC
         EEECCCCEEEEEEEECCCCCEECCCCEEEEEECCCCCCCCEEEEECCCEEEEEECCCCCCCCCCCCCCEEEEEEECCCC
Tom40-1  YIRKLK-NWNFGISYAISTA-LLPYLTIASTVELGR---SKVQTNVSDK-LISSLYSYKVSDNMNFEICGTLNHKAK--- 290
Tom40-3  YQRQVNPNWKFGITFATSKY-IEPRLQLTCKANLGK---STVHSSILTTGVVESKFTSKVSDTFTITVASILDHPAK--- 294
Tom40-2  HKHEINDKLTVGASLTADSN-LNSKVELAWIAKVND---AIIHSSIDTKLNVSTVYKQELYPQCNILVSTHLDHKNA--- 274
Tom40-4  LVKTINPQLSVGLQVRVNRMPAFVAADLSWIYTNKD---TQVHSIISTNGDVFSELSRKLNEKVALNVNCCLNHLEA--- 284
Tom40-5  GTHHPSDKTVSSAVLQFNPSTLQTDILFGFQRAFMS---SKVSAVMNTKGSVGSVFQKDVNEKYSIIISSFADHFQK--- 281
Tom40-6  ITYSPNEDTTGGIQLDILPSELRAKSAFFINRNFAM---TVFAFNVTSDATISSSLQRRFGKGYQMTLTSQADIFRK--- 287
Porin-1  SIYHPNLRNLFGANFTISHIGHGPEYLFMIGCSHNIGNDSSIRYVLRSDSKASFELNVPYKNFLKMTFTGNFEYSRANKL 289
Porin-2  LITSDFDKVRISSYLRANPFTFDSTATFGIQIPFGNSFGNISFSIPSKTIRFEAALNLQYEEDKKSGITVYWPFSPN--- 263


         CEEEEEEEEEECCCCCCCC
         CCEEEEEEEECC
         CCCEEEEEEEEEECCCC
         CCCEEEEEECCCC
         HHHEEEEEEEEEECCCCC
         HHHCCEEEEECC
         CCCCCEEEEEEECC
         CCCCCCC
Tom40-1  SYTIGLGFVFNPDDFVST 308
Tom40-3  NYKLGLGFYSQ------- 305
Tom40-2  QYNFGLGFQWVENSTN-- 290
Tom40-4  DYSFGIDLSINQ------ 296
Tom40-5  LYSLGMAVSVRDTSSD-- 297
Tom40-6  HYNFGLGLVFA------- 298
Porin-1  NSNFGAVIIFDKK----- 302
Porin-2  --TIKSVLA--------- 270
```
