# Supplementary material for: The Core Components of Organelle Biogenesis and Membrane Transport in the Hydrogenosomes of Trichomonas vaginalis
Source: PLoS One. 2011 Sep 15;6(9):e24428. doi: 10.1371/journal.pone.0024428 (PMC3174187; doi:10.1371/journal.pone.0024428)
Supplement: Figure S4 — Sequence alignment of Trichomonas vaginalis β-barrel proteins, Hmp35 and Hmp36. Cysteines and histidines of the putative metal binding motif, CX6CCX2CX9HX15CCXHXX2 C, are highlighted in yellow. Hmp35-1 (TVAG_590550), Hmp35-2 (TVAG_104250), Hmp36-1 (TVAG_031860) and Hmp36-2 (TVAG_216170). (PDF) [file pone.0024428.s004.pdf]

```
Hmp35-1  MEPKTFETVG DEATKLNIKP GLGVCVEENG TKVGVFTDLQ LQNPSINSSF KLP-VGTVDC  59
Hmp35-2  MEPKTFETVG DEATKLNIKP GLGVCVEENG TKVGVFTDLQ LQNPSINSSF KLP-VGTVDC  59
Hmp36-1  MEPIKYEKLG KKFTEIEFKP GVAVTLKQKK AECVLSVDEK IENPALSAEF KVKKAGKAIV  60
Hmp36-2  MNPDSYKKIG QKLTKLEYQS GFAVTLKYKK TKTTASCDEE FATPALTSEF KVKKVGKIQC  60

Hmp35-1  NIAKDGVVGV VLKPDLKQHG VTVSINPIEK NFTFSVKKEC TCTHYEATLG YNSAKKAAEA  119
Hmp35-2  NIAKDGVVGV VLKPDLKQHG VTVSINPIEK NFTFSVKKEC TCTHYEATLG YNSAKKAAEA  119
Hmp36-1  GFA-EGKPQI SIIPKLDKIK TELTINPLEN TFVFSTKKKI KKMKTKAIFG FDSAKLAPSL  119
Hmp36-2  GFK-DGKSQL IFSPKLEKLD AKLDFNAIDN SFLFEYAKKV KPINSLVTIK YDSAKNEPSV  119

Hmp35-1  HLYKQFAVKD SKVNCYLDIT GVNNTKPDVN YRIRYDLDKI GLRTYWD--G KDQRFAAFLD  177
Hmp35-2  HLYKQFAVKD SKVNCYLDIT GVNNTKPDVN YRIRYDLDKI GLRTYWD--G KDQRFAAFLD  177
Hmp36-1  LLMPKFKVNK MKVHADILLK KPEEDLPPVL FDAQAKIKKV NFVACFDEES KEYQAGVFAK  179
Hmp36-2  LIQPKFKLNK INFDANLLFK KPVENCPPVI FQAHAAYKKL FLCSCFNEEE KEYRAAAFAK  179

Hmp35-1  LKKAMIG-TH FFFN----KA VKSVDIYGLK SFKCGKASLI ATVLGEQR-C RLNFEG-CCK  230
Hmp35-2  LKKAMIG-TH FFFN----KA VKSVDIYGLK SFKCGKASLI ATVLGEQR-C RLNFEG-CCK  230
Hmp36-1  IAKKLHAGAL FHFQPTQENL LASIDLYSKL KYKGHKAGLI VSSLQTG--- KFNAQGKLGK  236
Hmp36-2  LFKGIHAGAL LHFDPLAEKQ MKAVDVFAKY SFKKGKIAAI TTLLGEKPVV KVNAKAKVGK  239

Hmp35-1  DCSYGAKIDY LHGEKPEITA KFGGASKCCS HFSCKYLATV TKKEA--TTF GIQTSGTFPL  288
Hmp35-2  DCSYGAKIDY LHGEKPEITA KFGGASKCCS HFSCKYLATV TKKEA--TTF GIQTSGTFPL  288
Hmp36-1  KVKYGLNVNY DKD----ING SFG-IKADLK KMDLNLVLKA TKEAEKEITP GLLGEAKFKI  291
Hmp36-2  KAKIGINTVY GET----ITG DFG-VKADLK KFKLNLIVNA KKESD-EISE GLIAQTAFKV  293

Hmp35-1  HGFGKATVGC ALADVKDYKN IGYSFLVE-- - 316
Hmp35-2  HGFGKATVGC ALADVKDYKN IGYSFLVELN - 318
Hmp36-1  KKLGKAKVGL SIPNLSDAKE INYFVNLKIK D 322
Hmp36-2  KRVGKAKVGV AIPSIQEAQK AHLFVNLKIK D 324
```
