# Supplementary material for: The Core Components of Organelle Biogenesis and Membrane Transport in the Hydrogenosomes of Trichomonas vaginalis
Source: PLoS One. 2011 Sep 15;6(9):e24428. doi: 10.1371/journal.pone.0024428 (PMC3174187; doi:10.1371/journal.pone.0024428)
Supplement: Figure S6 — Alignment of T. vaginalis Pam16 and Pam18 against eukaryotic orthologs. The diagnostic features identified in TvPam18 are (i) a J-domain at the C-terminus of the protein with a typical arrangement of three helixes and a short linker with a conserved HPDXGGS sequence motif connecting helixes II and III. The invariant HPD tripeptide is critical for the stimulation of the ATPase activity of Hsp70 by Pam18. (ii) A transmembrane α-helix that is close to the N-terminus. TvPam18 also contains a short N-terminal extension that is predicted to be a targeting presequence and a short N-terminal intermembrane space domain. However, TvPam18 does not contain a conserved interaction arm in front of helix I, which was structurally defined by [2] as one of the means by which Pam18 interacts with Pam16. The Pam16 protein family contains a degenerate J-domain with homology to Pam18 that lacks the HPD tripeptide in the linker motif; thus, it is unable to stimulate the ATPase activity of Hsp70. In TvPAM16, HPD is replaced by a D90, L91, E92 tripeptide, whereas the GGS motif of the linker is conserved. Importantly, TvPam16 contains a conserved L99 in the J-like domain that corresponds to L97 in the yeast ortholog. This residue has been shown to mediate an essential interaction between the Pam16 J-like domain and the J domain of Pam 18 that stabilizes the heterodimer [3]. A second characteristic feature predicted in TvPam16 is an N-terminal hydrophobic domain that is required for the interaction of Pam18:Pam16 heterodimer with the TIM23 translocon. The J-like domain of TvPam16 is underlined. Helical structures of the J-like domain (in red) were predicted by PSIPRED (http://bioinf.cs.ucl.ac.uk/psipred/). Arrowheads indicate the degenerate HPD motif of Pam16 between helixes II and III. The HPD motif of Pam18 is boxed. The leucine and asparagine residues essential for the Pam16-Pam18 interaction are marked with a star. Organisms and accession numbers: T. vaginalis, TVAG_470110; S. cerevisiae [file pone.0024428.s006.pdf]

```
                                                    CHHHHHHHH HEECCHHHHH HHHHHHHHHH HHCCCCCCHH
TvPam16                ---------- --------- -MVIVELAGL VISSTKVVCS TFINGFKHAA AANAPNGNAF  39
S.cerevisiae Pam16     ---------- --------- -MAHRAFIQV IITGTQVFGK AFAEAYRQ-- AASQSVKQGA  37
H.sapiens Pam16        ---------- --------- -MAK-YLAQI IVMGVQVVGR AFARALRQEF AASRAAADAR  38
D.discoideium Pam16    ---------- --------- -----MAARL IAKIVFTSGT VLVRSIQMAY KQALLQAES-  34
E.cuniculi Pam16       ---------- --------- --------ME AGKALMVLLG EGSK----VL WKAAVK----  24

                                      CHHHHH HHHHHHHHHH HHHHHHHHH     HHHHHHHH HHHHHHCCCC
TvPam18                ---------- ----MSIVNK FVEKALSLPT YAKAAIGAG- --VGLGVTSA VFSLFHTDKV  43
S.cerevisiae Pam18     MX35 VGIQNG SQGQKTGMDL YFDQALNYMG EHPVITGFGA FLTLYFTAGA YKSISKGLNG  59
H.sapiens Pam18        MASTVVAVGL TIAAAGFAGR YVLQAMKHME PQVKQ----- ---------V FQSLPKS---  43
```

```
                                  I. helix              II. helix
                       HHHCCCCCCC CCCCCCCHHH HHHHHCCCCC C-CCCHHHHH HHHHHHHHCC CCCCCCHHHH
TvPam16                QKFAGAVFGI QFQTRMMPDE ARQILGFEQK D-KLDIKSIK EHLDRMIKLN DLEKGGSPYL  98
S.cerevisiae Pam16     TNASRRGTGK GEYGGITLDE SCKILNIEES KGDLNMDKIN NRFNYLFEVN DKEKGGSFYL  97
H.sapiens Pam16        GRAGHRSAAA SNLSGLSLQE AQQILNVS-- --KLSPEEVQ KNYEHLFKVN DKSVGGSFYL  94
D.discoideium Pam16    -GMGAAAGSM DVKSKMSPIE ARKILGLEN- VETVSKEDID KKYNELLTIN DPKDGGSEYL  92
E.cuniculi Pam16       -TASDIIFRD ----RMSRME AEMVLDVTP- --STDKDKVR DAFLRIYCSN SKENGGSPYI  76

                       CCCCCCCCCC CCCCCCCHHH HHHHHCCCC    CCCHHHHH HHHHHHHHHH CCCCCCCHHH
TvPam18                VGLPPAANLN GFAPTLTRKE AELILNLPP- --NYTNQDIQ KHHRTLMALH HPDKGGSPYI  100
S.cerevisiae Pam18     GKSTTAFLKG GFDPKMNSKE ALQILNLTEN --TLTKKKLK EVHRKIMLAN HPDKGGSPFL  117
H.sapiens Pam18        -AFSGGYYRG GFEPKMTKRE AALILGVSP- --TANKGKIR DAHRRIMLLN HPDKGGSPYI  99
```

```
                        III. helix
                       HHHHHHHHHH HCC
TvPam16                NERFIAASHV LAK------- ---------- ---------- ---------- -- 111
S.cerevisiae Pam16     QSKVYRAAER LKWELAQREK NAKAKAGDAS TAKPPPNSTN SSGADNSASS NQ 149
H.sapiens Pam16        QSKVVRAKER LDEELKIQAQ EDREKGQMPH T--------- ---------- -- 125
D.discoideium Pam16    QIKISGAKHC LHSALKEGKK I--------- ---------- ---------- -- 113
E.cuniculi Pam16       QSRALAAYTV LSNTSLD-PP DCPSPETHQE AN-VSILSAK NKRT------ -- 118

                       HHHHHHHHHH HHHCCCC
TvPam18                ATKVNESRDF LTVGIRA--- ---------- ---------- ---------- -- 117
S.cerevisiae Pam18     ATKINEAKDF LEKRGISK-- ---------- ---------- ---------- -- 135
H.sapiens Pam18        AAKINEAKDL LEGQAKK--- ---------- ---------- ---------- -- 116
```
