# Supplementary material for: The Core Components of Organelle Biogenesis and Membrane Transport in the Hydrogenosomes of Trichomonas vaginalis
Source: PLoS One. 2011 Sep 15;6(9):e24428. doi: 10.1371/journal.pone.0024428 (PMC3174187; doi:10.1371/journal.pone.0024428)
Supplement: Figure S7 — Alignment of the Tim44 domain of T. vaginalis against eukaryotic and bacterial orthologs. Conserved hydrophobic residues that form the large hydrophobic pocket of Tim44 are highlighted in yellow [4], [5]. The conserved proline mutation, which causes familial oncocytic thyroid carcinoma, is in red [6]. Organisms and accession numbers: Saccharomyces cerevisiae, Q01852; Schizosaccharomyces pombe, NP_595905; Phytophora infestans, XP_002997475; Tribolium castaneum, XP_975336; Homo sapiens, NP_006342; Caenorhabiditis elegans, O02161; Caulobacter crestentus, AAK25703. (PDF) [file pone.0024428.s007.pdf]

```
T.vaginalis    ~~~NAFIRVP FQALANASSV VVSKLTQPSK EQLVQMSIQM YYPTFTISEF KQWIEKSFLP 200
S.cerevisiae   ~~~NPLIVV- MRKITNKVG- ---GFFAETE SSRVYSQFKL MDPTFSNESF TRHLREYIVP 299
S.pombe        ~~~HPIVSS- IRDMADSISG VWSRMFSETE ASQVMRRFKE IDPSFNTEHF LQYLREYIVP 295
P.infestans    ~~~EVWETS- QNPWVYRLSS IYDGLFGETP MAVAIKEIRR AEPDFILEEW KENIEEVVLP 339
T.castaneum    ~~~NPVIRA- SRLLTDKVSD IMGGLFQKTE LSETLTEICK IDPTFDTKKF LKQCETDIIP 297
H.sapiens      ~~~NAFIRA- SRALTDKVTD LLGGLFSKTE MSEVLTEILR VDPAFDKDRF LKQCENDIIP 325
C.elegans      ~~~NMAVRM- MRGVTEKIGS VFSG---QNE VSEVLTEIHK IDANFDKQEW LRFCETKIIP 297
C.crestentus   ~~~RRVGRQP EDAAVTAMPG AAPAPVDPVL QAEGVATLRA RQPDFDPARF LMGVR-AAYE 118

T.vaginalis    VLLERYLRGN IKQLEELTSQ QVAKERQLAV VQFLSNRLII -RTKLLSITD VDIMGFDFTN 259
S.cerevisiae   EILEAYVKGD VKVLKKWFSE APFNVYAAQQ KIFKEQDVYA -DGRILDIRG VEIVSAKLLA 358
S.pombe        EVTEAYVKGD KEVLKTWLSE APFSVYETTT KEYAKHGVVS -VGKILDIRG VDIMSQRLLQ 354
P.infestans    GVLEAFLRGN SRDLKKWFGE AAYSRMNIAI RERKSEGLVM -DPHVLSIDN VEVIEATAED 398
T.castaneum    NILEAMTRGD LEVLKDWCHE GPFNLFAIPI KEAYKKGYKI -DSKVLDVDN VDLVMGKVME 356
H.sapiens      NVLEAMISGE LDILKDWCYE ATYSQLAHPI QQAKALGLQF -HSRILDIDN VDLAMGKMME 384
C.elegans      NILEAFIRFD LEVLQSWCHE RAYTQLSTVV KEYQKMHFST KDSRIIDINK VEMATGKMME 357
C.crestentus   QIVKAYAEGD RETLTPLLAP DVMDNFERAM VARETAGRTE -KVEFLTPPR VDLERVDVVG 177

T.vaginalis    --RMPSILVR CSADHTNEVI TMNSGTIVEG GPQDICHTDF LVVLTIDAS- ---KDTPRWI 313
S.cerevisiae   PQDIPVLVVG CRAQEINLYR KKKTGEIAAG DEANILMSSY AMVFTRDPEQ IDDDETEGWK 418
S.pombe        PNDIPVFIVT FRTQEVHMFK DASSGELVAG KDDRIQQCTY ASVFTRVEDE LDNPETRGWR 414
P.infestans    -KQAPIILMR FQAQQINCIR NRE-GEVVEG SEDEVLAYYY IFAFQRDYD- -EEQETLRWR 454
T.castaneum    --QGPVLIIS FTSQQMMCVR DPN-GNVVEG DPEKVMRVAY VWVLCRDIS- -EPDPRAAWR 411
H.sapiens      --QGPVLIIT FQAQLVMVVR NPK-GEVVEG DPDKVLRMLY VWALCRDQD- -ELNPYAAWR 439
C.elegans      --QGPVLIIS FQVYMINVTK NAD-GKVVEG DPDNPKRINH IWVLCRDVE- -EYNPALAWK 412
C.crestentus   --DTAKAVVR ILAEVRTRTK DER------G EGVDDRRTAE LWTFEREVK- ---STDPNWH 225

T.vaginalis    ASELRPDSTS NRI 326
S.cerevisiae   ILEFVRGGSR QFT 431
S.pombe        IVDFARARAV DYF 427
P.infestans    IVDLHMQRGG RYY 467
T.castaneum    LLDLSASSNE QLV 424
H.sapiens      LLDISASSTE QIL 457
C.elegans      LLEVHMQETP LAL 425
C.crestentus   LTFVAAAEA- --- 324
```
