# Supplementary material for: The Core Components of Organelle Biogenesis and Membrane Transport in the Hydrogenosomes of Trichomonas vaginalis
Source: PLoS One. 2011 Sep 15;6(9):e24428. doi: 10.1371/journal.pone.0024428 (PMC3174187; doi:10.1371/journal.pone.0024428)
Supplement: References S1 — References for the supporting information figures and tables. (DOC) [file pone.0024428.s010.doc]

**Supplementary** **References**

1. Pebay-Peyroula E, Dahout-Gonzalez C, Kahn R, Trezeguet V, Lauquin GJ et al. (2003) Structure of mitochondrial ADP/ATP carrier in complex with carboxyatractyloside. Nature 426: 39-44.

2. Mokranjac D, Bourenkov G, Hell K, Neupert W, Groll M (2006) Structure and function of Tim14 and Tim16, the J and J-like components of the mitochondrial protein import motor. EMBO J 25: 4675-4685.

3. D'Silva PR, Schilke B, Hayashi M, Craig EA (2008) Interaction of the J-protein heterodimer Pam18/Pam16 of the mitochondrial import motor with the translocon of the inner membrane. Mol Biol Cell 19: 424-432.

4. Handa N, Kishishita S, Morita S, Akasaka R, Jin ZM et al. (2007) Structure of the human Tim44 C-terminal domain in complex with pentaethylene glycol: ligand-bound form. Acta Crystallogr Sec D-Biol Crystallogr 63: 1225-1234.

5. Josyula R, Jin ZM, Fu ZQ, Sha BD (2006) Crystal structure of yeast mitochondrial peripheral membrane protein Tim44p C-terminal domain. J Mol Biol 359: 798-804.

6. Bonora E, Evangelisti C, Bonichon F, Tallini G, Romeo G (2006) Novel germline variants identified in the inner mitochondrial membrane transporter TIMM44 and their role in predisposition to oncocytic thyroid carcinomas. Br J Cancer 95: 1529-1536.

7. D'Silva P, Liu QL, Walter W, Craig EA (2004) Regulated interactions of mtHsp70 with Tim44 at the translocon in the mitochondrial inner membrane. Nat Struct Mol Biol 11: 1084-1091.

8. Marom M, Safonov R, Amram S, Avneon Y, Nachliel E et al. (2009) Interaction of the Tim44 C-terminal domain with negatively charged phospholipids. Biochemistry 48: 11185-95

9. Guschina IA, Harris KM, Maskrey B, Goldberg B, Lloyd D et al. (2009) The microaerophilic flagellate, *Trichomonas vaginalis*, contains unusual acyl lipids but no detectable cardiolipin. J Euk Microbiol 56: 52-57.
