# Supplementary material for: The Core Components of Organelle Biogenesis and Membrane Transport in the Hydrogenosomes of Trichomonas vaginalis
Source: PLoS One. 2011 Sep 15;6(9):e24428. doi: 10.1371/journal.pone.0024428 (PMC3174187; doi:10.1371/journal.pone.0024428)
Supplement: Table S1 — Complete list of proteins identified by nanoLC MS/MS in four independent experiments. (DOC) [file pone.0024428.s011.doc]

**Table S1.** Complete list of proteins identified by nanoLC MS/MS in four independent experiments.

| **TrichDB** | **LC MS/MS** | | | | | | | |
| --- | --- | --- | --- | --- | --- | --- | --- | --- |
| accession No. | #1 | | #2 | | #3 | | #4 | |
|  | score | peptides | score | peptides | score | peptides | score | peptides |
| TVAG_399510 | 44 | 2 | 282 | 6 |  |  | 79 | 2 |
| TVAG_332970 |  |  | 82 | 2 |  |  |  |  |
| TVAG_450220 | 303 | 5 | 293 | 6 | 67 | 1 | 276 | 5 |
| TVAG_123100 |  |  |  |  | 195 | 4 |  |  |
| TVAG_341190 | 36 | 1 |  |  |  |  |  |  |
| TVAG_195900 | 314 | 5 | 518 | 8 |  |  |  |  |
| TVAG_178100 |  |  |  |  | 1297 | 31 | 560 | 13 |
| TVAG_287510 |  |  |  |  | 82 | 2 |  |  |
| TVAG_026080 |  |  |  |  | 150 | 4 | 104 | 2 |
| TVAG_198350 |  |  |  |  | 94 | 2 |  |  |
| TVAG_370860 |  |  | 48 | 1 | 76 | 2 | 74 | 1 |
| TVAG_061900 | 103 | 2 | 123 | 4 | 146 | 5 |  |  |
| TVAG_379950 |  |  |  |  | 242 | 4 |  |  |
| TVAG_447580 | 284 | 5 | 200 | 6 | 275 | 10 | 45 | 1 |
| TVAG_008790 |  |  |  |  | 430 | 6 |  |  |
| TVAG_470110 | 352 | 5 | 302 | 14 | 485 | 15 | 196 | 3 |
| TVAG_436580 | 114 | 5 | 341 | 12 | 188 | 7 | 172 | 5 |
| TVAG_146920 |  |  |  |  |  |  |  |  |
| TVAG_340380 |  |  | 110 | 2 | 85 | 2 |  |  |
| TVAG_590550 | 1313 | 21 |  |  |  |  |  |  |
| TVAG_104250 | 1599 | 25 | 306 | 9 | 2113 | 141 |  |  |
| TVAG_031860 | 1593 | 22 |  |  | 2491 | 61 | 1245 | 19 |
| TVAG_216170 | 1711 | 19 | 60 | 1 | 2443 | 122 |  |  |
| TVAG_237680 | 1400 | 25 | 792 | 34 | 664 | 42 |  |  |
| TVAG_051820 | 672 | 16 | 496 | 28 | 1158 | 145 |  |  |
| TVAG_164560 | 48 | 2 | 40 | 1 | 248 | 4 |  |  |
| TVAG_196220 |  |  |  |  | 154 | 3 |  |  |
| TVAG_262210 | 222 | 5 | 182 | 3 |  |  |  |  |
| TVAG_039960 |  |  | 100 | 4 |  |  |  |  |
| TVAG_455090 | 807 | 12 | 782 | 18 | 1009 | 42 |  |  |
| TVAG_489980 | 36 | 3 |  |  |  |  |  |  |
| TVAG_127990 |  |  |  |  |  |  | 54 | 3 |
| TVAG_440200 | 831 | 16 | 862 | 37 | 1449 | 95 |  |  |
| TVAG_136450 |  |  | 162 | 3 |  |  |  |  |
| TVAG_090120 | 57 | 2 |  |  |  |  |  |  |
| TVAG_192370 | 55 | 2 |  |  | 244 | 7 | 173 | 4 |
| TVAG_190830 |  |  |  |  | 152 | 4 |  |  |
| TVAG_458060 |  |  |  |  | 103 | 2 |  |  |
| TVAG_272350 | 37 | 2 |  |  | 468 | 13 |  |  |
| TVAG_240680 |  |  |  |  | 299 | 5 |  |  |
| TVAG_137270 | 458 | 9 | 543 | 12 | 802 | 22 | 234 | 7 |
| TVAG_277930 | 329 | 5 | 652 | 12 | 210 | 5 | 146 | 4 |
| TVAG_283120 | 412 | 6 | 409 | 7 | 543 | 16 | 368 | 5 |
| TVAG_174010 |  |  | 195 | 3 | 409 | 7 |  |  |
| TVAG_369980 | 87 | 3 | 146 | 3 | 230 | 9 | 65 | 1 |
| TVAG_393390 |  |  | 306 | 6 | 283 | 8 | 149 | 4 |
| TVAG_211970 | 44 | 1 |  |  | 85 | 4 |  |  |
| TVAG_032990 |  |  |  |  | 114 | 2 |  |  |
| TVAG_080160 | 45 | 2 |  |  |  |  |  |  |
| TVAG_094480 |  |  | 37 | 1 |  |  | 39 | 2 |
| TVAG_152710 | 37 | 1 | 35 | 2 |  |  |  |  |
| TVAG_178320 |  |  | 140 | 2 |  |  |  |  |
| TVAG_182990 | 39 | 1 | 33 | 2 | 40 | 1 |  |  |
| TVAG_210010 | 51 | 3 |  |  |  |  |  |  |
| TVAG_218130 |  |  | 348 | 6 | 34 | 1 |  |  |
| TVAG_225560 |  |  |  |  | 115 | 5 |  |  |
| TVAG_251750 |  |  |  |  |  |  | 35 | 2 |
| TVAG_252220 | 36 | 2 |  |  |  |  |  |  |
| TVAG_295140 |  |  |  |  |  |  | 39 | 3 |
| TVAG_331680 | 41 | 2 |  |  |  |  |  |  |
| TVAG_333160 |  |  |  |  | 56 | 2 |  |  |
| TVAG_337270 | 38 | 2 | 36 | 1 |  |  |  |  |
| TVAG_341690 | 39 | 2 | 33 | 1 |  |  | 41 | 2 |
| TVAG_370950 | 135 | 2 |  |  |  |  |  |  |
| TVAG_403380 | 35 | 2 |  |  |  |  |  |  |
| TVAG_413430 | 36 | 2 |  |  |  |  |  |  |
| TVAG_425430 | 39 | 2 |  |  |  |  |  |  |
| TVAG_423530 | 181 | 6 | 180 | 6 | 349 | 9 | 170 | 5 |
| TVAG_239660 |  |  |  |  | 250 | 6 |  |  |
| TVAG_432650 |  |  |  |  | 275 | 3 |  |  |
| TVAG_008840 |  |  |  |  | 48 | 2 |  |  |
| TVAG_456770 |  |  |  |  | 159 | 3 |  |  |
| TVAG_027170 |  |  | 39 | 1 | 48 | 1 |  |  |
| TVAG_076230 |  |  | 248 | 5 | 135 | 3 |  |  |
| TVAG_217870 | 40 | 1 |  |  | 89 | 2 |  |  |
| TVAG_257780 | 454 | 9 | 57 | 1 | 644 | 17 |  |  |
| TVAG_296220 |  |  | 37 | 1 | 142 | 4 |  |  |
| TVAG_133030 | 194 | 6 | 53 | 1 | 1100 | 34 |  |  |
| TVAG_113870 | 92 | 3 |  |  |  |  |  |  |
| TVAG_164890 | 282 | 6 | 156 | 7 | 1131 | 42 |  |  |
| TVAG_060450 |  |  | 56 | 1 | 368 | 12 |  |  |
| TVAG_270750 | 89 | 2 | 240 | 3 | 118 | 4 |  |  |
| TVAG_489800 |  |  |  |  | 359 | 6 |  |  |
| TVAG_003900 | 75 | 1 | 710 | 22 | 905 | 31 |  |  |
| TVAG_037570 | 1619 | 34 | 1022 | 29 | 1784 | 52 |  |  |
| TVAG_361590 | 649 | 16 |  |  |  |  |  |  |
| TVAG_182620 | 118 | 3 | 200 | 4 | 714 | 20 |  |  |
| TVAG_310050 |  |  | 91 | 2 | 304 | 7 |  |  |
| TVAG_267870 |  |  | 500 | 10 | 1994 | 113 |  |  |
| TVAG_238830 |  |  | 820 | 17 | 2867 | 155 |  |  |
| TVAG_412220 |  |  |  |  | 2153 | 118 |  |  |
| TVAG_340290 |  |  | 601 | 13 | 2435 | 138 |  |  |
| TVAG_068130 |  |  |  |  | 1044 | 30 |  |  |
| TVAG_183790 |  |  |  |  | 1850 | 107 |  |  |
| TVAG_198110 |  |  | 1596 | 31 | 1038 | 21 |  |  |
| TVAG_230580 |  |  | 1552 | 29 | 1045 | 21 |  |  |
| TVAG_242960 |  |  | 1604 | 28 | 1019 | 21 |  |  |
| TVAG_254890 |  |  | 585 | 13 |  |  |  |  |
| TVAG_165340 |  |  |  |  | 644 | 16 |  |  |
| TVAG_318670 |  |  |  |  | 552 | 16 |  |  |
| TVAG_144730 |  |  | 839 | 24 | 2315 | 107 |  |  |
| TVAG_259190 |  |  | 357 | 10 | 2147 | 69 |  |  |
| TVAG_183500 | 76 | 3 | 778 | 21 | 2571 | 109 |  |  |
| TVAG_036010 | 47 | 1 |  |  | 655 | 14 |  |  |
| TVAG_055200 | 628 | 11 | 407 | 8 | 831 | 19 | 350 | 5 |
| TVAG_064490 |  |  | 145 | 5 | 320 | 10 |  |  |
| TVAG_275660 |  |  | 102 | 2 |  |  |  |  |
| TVAG_206500 |  |  |  |  | 519 | 8 |  |  |
| TVAG_121620 |  |  |  |  | 85 | 2 |  |  |
| TVAG_088050 | 76 | 3 |  |  | 101 | 2 |  |  |
| TVAG_237140 | 347 | 9 |  |  | 837 | 19 |  |  |
| TVAG_340390 | 299 | 8 | 113 | 2 | 765 | 14 |  |  |
| TVAG_433130 |  |  | 101 | 1 | 277 | 4 |  |  |
| TVAG_197980 | 188 | 5 | 42 | 4 | 148 | 3 |  |  |
| TVAG_287530 | 370 | 6 | 164 | 3 | 350 | 8 | 116 | 3 |
| TVAG_381290 | 416 | 5 | 252 | 4 | 418 | 13 | 82 | 1 |
| TVAG_119710 |  |  |  |  | 146 | 2 |  |  |
| TVAG_233350 |  |  |  |  | 149 | 3 |  |  |
| TVAG_063000 |  |  |  |  | 42 | 1 |  |  |
| TVAG_043720 |  |  | 30 | 1 | 80 | 2 |  |  |
| TVAG_074600 |  |  |  |  | 618 | 11 |  |  |
| TVAG_088220 |  |  | 61 | 1 | 792 | 19 |  |  |
| TVAG_132440 |  |  | 36 | 1 | 359 | 7 |  |  |
| TVAG_183850 |  |  |  |  | 166 | 3 |  |  |
| TVAG_344520 |  |  |  |  | 45 | 2 |  |  |
| TVAG_379550 | 927 | 20 | 37 | 1 | 1059 | 22 |  |  |
| TVAG_177600 |  |  |  |  | 168 | 2 |  |  |
| TVAG_496160 |  |  |  |  | 985 | 29 |  |  |
| TVAG_293770 | 566 | 10 | 393 | 12 | 858 | 34 |  |  |
| TVAG_462920 | 187 | 5 | 232 | 7 | 933 | 44 |  |  |
| TVAG_321010 | 232 | 9 | 145 | 4 | 670 | 13 |  |  |
| TVAG_075420 | 87 | 5 |  |  |  |  |  |  |
| TVAG_095250 | 99 | 4 |  |  |  |  |  |  |
| TVAG_455310 |  |  | 115 | 6 |  |  |  |  |
| TVAG_350540 | 87 | 2 | 142 | 5 | 168 | 6 | 70 | 2 |
| TVAG_528900 | 99 | 4 |  |  |  |  | 88 | 3 |
| TVAG_152690 |  |  |  |  | 544 | 15 |  |  |
| TVAG_272880 |  |  |  |  | 178 | 3 |  |  |
| TVAG_351540 |  |  |  |  | 389 | 8 |  |  |
| TVAG_044510 |  |  |  |  | 114 | 2 |  |  |
| TVAG_381470 |  |  |  |  | 233 | 5 |  |  |
| TVAG_479220 | 40 | 2 |  |  |  |  |  |  |
| TVAG_491770 |  |  |  |  | 85 | 2 |  |  |
| TVAG_125390 |  |  |  |  |  |  | 38 | 3 |
| TVAG_132350 | 1185 | 4 | 37 | 1 | 561 | 9 |  |  |
| TVAG_165530 | 37 | 3 |  |  |  |  |  |  |
| TVAG_228770 | 39 | 4 |  |  |  |  |  |  |
| TVAG_433790 | 35 | 2 |  |  |  |  |  |  |
| TVAG_074970 | 80 | 3 | 46 | 1 |  |  | 139 | 4 |
| TVAG_082020 |  |  |  |  | 99 | 2 |  |  |
| TVAG_054030 |  |  | 42 | 1 | 914 | 19 |  |  |
| TVAG_215920 |  |  |  |  | 103 | 2 |  |  |
| TVAG_026390 |  |  |  |  | 141 | 4 |  |  |
| TVAG_248450 |  |  | 35 | 1 | 203 | 4 |  |  |
| TVAG_473170 |  |  | 31 | 1 | 122 | 4 |  |  |
| TVAG_273260 |  |  |  |  | 296 | 5 |  |  |
| TVAG_043500 |  |  |  |  | 117 | 3 |  |  |
| TVAG_397250 |  |  |  |  | 409 | 6 | 114 | 3 |
| TVAG_146910 |  |  |  |  | 369 | 11 |  |  |
| TVAG_043060 |  |  | 34 | 1 | 679 | 25 |  |  |
| TVAG_271850 |  |  | 73 | 2 | 56 | 2 |  |  |
| TVAG_172700 |  |  | 31 | 2 |  |  | 36 | 1 |
| TVAG_283360 |  |  |  |  |  |  | 25 | 2 |
| TVAG_102720 | 37 | 2 |  |  |  |  | 42 | 2 |
| TVAG_315350 |  |  |  |  | 33 | 1 |  |  |
| TVAG_317590 | 37 | 2 |  |  |  |  |  |  |
| TVAG_019240 |  |  |  |  | 110 | 16 |  |  |
| TVAG_064150 |  |  | 48 | 2 | 72 | 2 |  |  |
| TVAG_137880 |  |  |  |  | 253 | 4 |  |  |
| TVAG_045010 |  |  |  |  | 219 | 3 |  |  |
| TVAG_219820 |  |  |  |  | 70 | 2 |  |  |
| TVAG_234440 |  |  |  |  | 507 | 10 |  |  |
| TVAG_075320 |  |  | 55 | 1 | 223 | 9 |  |  |
| TVAG_030480 |  |  | 96 | 1 | 140 | 4 |  |  |
| TVAG_311860 | 36 | 1 |  |  | 215 | 4 |  |  |
| TVAG_118780 | 55 | 1 | 298 | 10 | 663 | 15 |  |  |
| TVAG_026290 |  |  |  |  | 163 | 2 |  |  |
| TVAG_229870 |  |  |  |  | 96 | 2 |  |  |
| TVAG_277050 |  |  |  |  | 178 | 7 |  |  |
| TVAG_321030 |  |  | 81 | 2 | 413 | 7 |  |  |
| TVAG_342900 | 293 | 6 |  |  | 293 | 5 |  |  |
| TVAG_367660 |  |  | 31 | 1 |  |  |  |  |
| TVAG_239840 |  |  |  |  | 54 | 1 |  |  |
| TVAG_022120 |  |  |  |  | 198 | 4 |  |  |
| TVAG_026100 | 270 | 8 |  |  | 483 | 9 | 173 | 4 |
| TVAG_028050 | 71 | 2 | 37 | 3 | 331 | 11 |  |  |
| TVAG_038870 |  |  |  |  | 443 | 15 |  |  |
| TVAG_044000 |  |  |  |  | 142 | 2 |  |  |
| TVAG_067030 |  |  |  |  | 355 | 9 |  |  |
| TVAG_074260 | 41 | 2 |  |  |  |  |  |  |
| TVAG_089110 |  |  |  |  |  |  | 38 | 2 |
| TVAG_090740 | 37 | 2 |  |  |  |  |  |  |
| TVAG_102740 | 43 | 1 |  |  | 595 | 10 |  |  |
| TVAG_103110 | 74 | 2 | 69 | 3 |  |  | 44 | 2 |
| TVAG_104680 |  |  |  |  | 170 | 3 |  |  |
| TVAG_113880 |  |  |  |  | 626 | 12 |  |  |
| TVAG_140620 |  |  |  |  | 439 | 10 |  |  |
| TVAG_165290 |  |  |  |  | 71 | 2 |  |  |
| TVAG_165320 |  |  |  |  | 77 | 2 |  |  |
| TVAG_178820 | 51 | 3 |  |  |  |  |  |  |
| TVAG_197920 |  |  |  |  | 37 | 2 |  |  |
| TVAG_209310 | 58 | 2 |  |  | 208 | 6 |  |  |
| TVAG_225570 |  |  | 50 | 3 |  |  |  |  |
| TVAG_225930 |  |  |  |  | 420 | 7 |  |  |
| TVAG_237550 |  |  |  |  | 138 | 4 |  |  |
| TVAG_241570 | 39 | 2 | 32 | 2 |  |  | 39 | 2 |
| TVAG_249920 | 71 | 1 | 48 | 2 | 144 | 2 |  |  |
| TVAG_301190 | 38 | 2 |  |  |  |  | 36 | 1 |
| TVAG_321550 | 42 | 2 |  |  |  |  |  |  |
| TVAG_334260 |  |  | 37 | 2 |  |  |  |  |
| TVAG_343040 | 125 | 4 | 282 | 6 | 185 | 4 | 99 | 3 |
| TVAG_392650 |  |  |  |  | 241 | 4 |  |  |
| TVAG_415360 | 35 | 3 |  |  |  |  |  |  |
| TVAG_416630 |  |  |  |  | 319 | 6 |  |  |
| TVAG_430700 | 38 | 2 |  |  |  |  |  |  |
| TVAG_431100 | 69 | 3 |  |  |  |  |  |  |
| TVAG_433120 |  |  |  |  |  |  | 27 | 1 |
| TVAG_437350 | 73 | 2 |  |  |  |  |  |  |
| TVAG_442170 |  |  |  |  | 523 | 11 |  |  |
| TVAG_445430 | 40 | 4 |  |  |  |  |  |  |
| TVAG_450060 | 150 | 5 |  |  | 554 | 11 |  |  |
| TVAG_454330 | 37 | 1 | 135 | 6 | 214 | 8 |  |  |
| TVAG_483050 | 38 | 2 |  |  |  |  | 36 | 2 |
| TVAG_483980 | 56 | 3 |  |  | 126 | 2 |  |  |
| TVAG_493810 | 58 | 2 | 96 | 2 | 491 | 10 |  |  |
| TVAG_521380 |  |  |  |  | 66 | 2 |  |  |
| TVAG_547420 |  |  | 35 | 1 | 154 | 3 |  |  |
| TVAG_607400 |  |  |  |  |  |  | 34 | 2 |
