# Supplementary material for: The Core Components of Organelle Biogenesis and Membrane Transport in the Hydrogenosomes of Trichomonas vaginalis
Source: PLoS One. 2011 Sep 15;6(9):e24428. doi: 10.1371/journal.pone.0024428 (PMC3174187; doi:10.1371/journal.pone.0024428)
Supplement: Table S2 — Identification of hydrogenosomal proteins using TrichDB (http://trichdb.org/trichdb/), Uniprot (http://www.uniprot.org/), and PFAM A+B (http://pfam.sanger.ac.uk/) searches. (DOC) [file pone.0024428.s012.doc]

**Table S2.** Identification of hydrogenosomal proteins using TrichDB (<http://trichdb.org/trichdb/>), Uniprot (<http://www.uniprot.org/>), and PFAM A+B (<http://pfam.sanger.ac.uk/>) searches.

| **TrichDB 1.2** | **Identification** | | | | | |
| --- | --- | --- | --- | --- | --- | --- |
|  | **Name** | **TrichDB 1.2 annotation** | **UNIPROT** | | **PFAM A+B** | |
| Acc. No. |  |  | Acc. No. | Uniprot annotated proteins | Protein families | e-value |
| TVAG_399510 | Tom40-1 | conserved hypothetical protein | A2E5Y9 | Putative uncharacterized protein | Porin_3 | 7.50E-002 |
| TVAG_332970 | Tom40-2 | conserved hypothetical protein | A2EH93 | Putative uncharacterized protein | Porin_3 | 2.70E-018 |
| TVAG_450220 | Tom40-3 | conserved hypothetical protein | A2G8G1 | Putative uncharacterized protein | Porin_3 | 7.20E-008 |
| TVAG_123100 | Tom40-4 | conserved hypothetical protein | A2FH81 | Putative uncharacterized protein | Porin_3 | 3.50E-011 |
| TVAG_341190 | Tom40-5 | hypothetical protein | A2DTS8 | Putative uncharacterized protein | Porin_3 | 1.60E-012 |
| TVAG_195900 | Tom40-6 | conserved hypothetical protein | A2ETM9 | Putative uncharacterized protein | Porin_3 | 3.10E-002 |
| TVAG_178100 | Sam50 | hypothetical protein | A2DIG2 | Putative uncharacterized protein | Bac_surface_Ag | 2.00E-004 |
| TVAG_287510 | small Tim9-10A | conserved hypothetical protein | A2FKF0 | Putative uncharacterized protein | zf-Tim10_DDP | 6.00E-001 |
| TVAG_026080 | small Tim9-10B | conserved hypothetical protein | A2DZ11 | Putative uncharacterized protein | zf-Tim10_DDP | 5.50E-001 |
| TVAG_198350 | Tim17/22/23A | hypothetical protein | A2DDM1 | Mitochondrial import inner membrane translocase subunit Tim17 family protein | Tim17 | 7.40E-008 |
| TVAG_370860 | Tim17/22/23B | conserved hypothetical protein | A2FHN8 | Putative uncharacterized protein | Tim17 | 1.40E-009 |
| TVAG_061900 | Tim17/22/23C | conserved hypothetical protein | A2E7V5 | Mitochondrial import inner membrane translocase subunit Tim17 family protein | Tim17 | 1.30E-001 |
| TVAG_379950 | Tim17/22/23D | conserved hypothetical protein | A2DXD3 | Putative uncharacterized protein | BenE | 4.50E-001 |
| TVAG_447580 | Tim17-like | conserved hypothetical protein | A2DS29 | Putative uncharacterized protein | Tim17 | 1.30E+000 |
| TVAG_008790 | Tim44 | conserved hypothetical protein | A2F7V1 | Putative uncharacterized protein | Tim44 | 2.80E-014 |
| TVAG_470110 | Pam16 | conserved hypothetical protein | A2FE18 | Putative uncharacterized protein | Pam16 | 1.10E-020 |
| TVAG_436580 | Pam18 | S.cerevisiae chromosome XII reading frame orf ylr008c, putative | A2DFA6 | DnaJ domain containing protein | Pam16 | 1.00E-010 |
| TVAG_146920 | Porin-1 | hypothetical |  |  | DUF3130 | 2.90E+000 |
| TVAG_340380 | Porin-2 | conserved hypothetical protein | A2EKF2 | Putative uncharacterized protein | Hemolysin_N | 6.90E+000 |
| TVAG_590550 | Hmp-35-1 | conserved hypothetical protein | A2HE83 | Putative uncharacterized protein | Exon_PolB | 7.80E+000 |
| TVAG_104250 | Hmp-35-2 | conserved hypothetical protein | A2FTN9 | Hydrogenosomal membrane protein Hmp35 | Exon_PolB | 8.20E+000 |
| TVAG_031860 | Hmp-36-1 | conserved hypothetical protein | A2EUI5 | Putative uncharacterized protein | DUF3406 | 1.80E+000 |
| TVAG_216170 | Hmp-36-2 | conserved hypothetical protein | A2ENV1 | Putative uncharacterized protein | NARG2_C | 8.90E+000 |
| TVAG_237680 | ADP/ATP carrier-1, Hmp-31 | ADP,ATP carrier protein, putative | A2DCW8 | Hydrogenosomal membrane protein 31 | Mito_carr | 1.70E-014 |
| TVAG_051820 | ADP/ATP carrier-2 | tricarboxylate transport protein, putative | A2FII9 | Mitochondrial carrier protein | Mito_carr | 5.70E-014 |
| TVAG_164560 | ADP/ATP carrier-3 | mitochondrial carrier protein, putative | A2E1Z7 | Mitochondrial carrier protein | Mito_carr | 2.80E-008 |
| TVAG_196220 | ADP/ATP carrier-4 | protein brittle-1, chloroplast precursor, putative | A2F4V4 | Mitochondrial carrier protein | Mito_carr | 8.80E-007 |
| TVAG_262210 | ADP/ATP carrier-5 | tricarboxylate transport protein, putative | A2DUD3 | Mitochondrial carrier protein | Mito_carr | 1.80E-012 |
| TVAG_039960 | MFS-28 | sugar transporter, putative | A2EQX0 | Putative uncharacterized protein | Sugar_tr | 1.70E-018 |
| TVAG_455090 | Unknown (Rhomboid-family) | conserved hypothetical protein | A2FMX1 | Putative uncharacterized protein | Glucokinase | 7.20E+000 |
| TVAG_489980 | Unknown | hypothetical | A2FB35 | Putative uncharacterized protein | UvrD-helicase | 1.50E-001 |
| TVAG_127990 | Unknown | hypothetical | A2EBG0 | Putative uncharacterized protein | Baculo_VP1054 | 7.30E-001 |
| TVAG_440200 | Unknown | conserved hypothetical protein | A2FDM3 | Putative uncharacterized protein | EIAV_Rev | 2.10E-001 |
| TVAG_136450 | Unknown | conserved hypothetical protein | A2DJC7 | Putative uncharacterized protein | U-box | 5.00E-007 |
| TVAG_090120 | C-tail-1 | hypothetical | A2EZN9 | Putative uncharacterized protein | NPDC1 | 5.50E+000 |
| TVAG_192370 | C-tail-2 | conserved hypothetical protein | A2DGV1 | Putative uncharacterized protein | DUF2648 | 1.30E+000 |
| TVAG_190830 | C-tail-3 | conserved hypothetical protein | A2EFG6 | Putative uncharacterized protein | Nfu_N | 6.80E-002 |
| TVAG_458060 | C-tail-4 | conserved hypothetical protein | A2FKT7 | Putative uncharacterized protein | DUF769 | 1.10E-002 |
| TVAG_272350 | C-tail-5 | conserved hypothetical protein | A2FDV1 | Putative uncharacterized protein | Pneumo_phosprot | 2.60E+000 |
| TVAG_240680 | C-tail-6 | conserved hypothetical protein | A2EJB5 | Surface antigen BspA-like | ETRAMP | 7.30E-001 |
| TVAG_137270 | C-tail-7 | conserved hypothetical protein | A2FNS9 | Putative uncharacterized protein | Trypan_PARP | 1.40E-003 |
| TVAG_277930 | C-tail-8 | conserved hypothetical protein | A2DU34 | Putative uncharacterized protein | ArsR | 1.70E+000 |
| TVAG_283120 | C-tail-9 | hypothetical protein | A2DEL5 | Putative uncharacterized protein | CD99L2 | 6.00E-001 |
| TVAG_174010 | C-tail-10 | conserved hypothetical protein | A2EWU4 | Putative uncharacterized protein | DUF812 | 1.30E+000 |
| TVAG_369980 | C-tail-11 | conserved hypothetical protein | A2EX50 | Putative uncharacterized protein | TMEMspv1-c74-12 | 3.90E+000 |
| TVAG_393390 | C-tail-12 | conserved hypothetical protein | A2DYD5 | Putative uncharacterized protein | A2M_N | 4.70E-001 |
| TVAG_211970 | C-tail-13 | conserved hypothetical protein | A2EIF3 | Putative uncharacterized protein | GRA6 | 1.90E-001 |
| TVAG_032990 | Unknown | conserved hypothetical protein | A2FAZ8 | Putative uncharacterized protein | U-box | 3.90E-006 |
| TVAG_080160 | Unknown | hypothetical | A2FBG9 | Putative uncharacterized protein | Cortex-I_coil | 8.80E-002 |
| TVAG_094480 | Unknown | conserved hypothetical protein | A2DBS0 | Putative uncharacterized protein | Phage_tail_T | 8.70E+000 |
| TVAG_152710 | Unknown | conserved hypothetical protein | A2FWL9 | Putative uncharacterized protein | MAD | 2.70E-002 |
| TVAG_178320 | Unknown | hypothetical protein | A2DII4 | Putative uncharacterized protein | Lectin_N | 5.40E+000 |
| TVAG_182990 | Unknown | hypothetical protein | A2D937 | Putative uncharacterized protein | AAA_5 | 8.20E-003 |
| TVAG_210010 | Unknown | hypothetical | A2DVQ8 | Putative uncharacterized protein |  | 0.00E+000 |
| TVAG_218130 | Unknown | conserved hypothetical protein | A2FD06 | Putative uncharacterized protein | MGC-24 | 8.90E-001 |
| TVAG_225560 | Unknown | hypothetical protein | A2DNT5 | Putative uncharacterized protein | PhnJ | 8.40E-001 |
| TVAG_251750 | Unknown | hypothetical | A2EF33 | Putative uncharacterized protein | HOOK | 5.20E-003 |
| TVAG_252220 | Unknown | hypothetical | A2DVW4 | Putative uncharacterized protein | FERM_M | 1.80E-002 |
| TVAG_295140 | Unknown | hypothetical | A2DL71 | Putative uncharacterized protein | DUF3766 | 4.60E+000 |
| TVAG_331680 | Unknown | hypothetical | A2G319 | Putative uncharacterized protein | NAD_binding_1 | 2.50E+000 |
| TVAG_333160 | Unknown | conserved hypothetical protein | A2EHB2 | Putative uncharacterized protein | DNA_binding_1 | 3.50E-001 |
| TVAG_337270 | Unknown | conserved hypothetical protein | A2FS15 | Putative uncharacterized protein | Rap_GAP | 1.10E-002 |
| TVAG_341690 | Unknown | conserved hypothetical protein | A2G694 | Putative uncharacterized protein | Creb_binding | 5.40E+000 |
| TVAG_370950 | Unknown | hypothetical | A2FHP7 | Putative uncharacterized protein | DUF829 | 4.60E-036 |
| TVAG_403380 | Unknown | hypothetical | A2F8X4 | Putative uncharacterized protein | Tenui_PVC2 | 1.60E+000 |
| TVAG_413430 | Unknown | hypothetical | A2F9H5 | Putative uncharacterized protein | CotJA | 2.20E+000 |
| TVAG_425430 | Unknown | hypothetical | A2G2K9 | Putative uncharacterized protein | ATG_C | 3.80E-007 |
| TVAG_423530 | Unknown | conserved hypothetical protein | A2DTJ1 | Putative uncharacterized protein | PAS_2 | 6.30E-001 |
| TVAG_239660 | IscS-2, cystein desulfurase | cysteine desulfurylase, putative | A2EFA9 | IscS/NifS-like protein | Aminotran_5 | 1.20E-039 |
| TVAG_432650 | IscU | nitrogen fixation protein nifu, putative | A2DIQ6 | NifU-like protein, putative | NifU_N | 8.90E-030 |
| TVAG_008840 | Nfu-2 | conserved hypothetical protein | A2FGS9 | NifU-like domain containing protein | NifU | 2.40E-014 |
| TVAG_456770 | IscA2-1 | iron-sulfur cluster assembly protein, putative | A2DC01 | HesB-like domain containing protein | Fe-S_biosyn | 7.70E-018 |
| TVAG_027170 | Ind-1 (P-Loop ATPase) | nucleotide binding protein, putative | A2F1G2 | Mrp, putative | ParA | 4.90E-022 |
| TVAG_076230 | Ind-2 (P-loop ATPase) | nucleotide binding protein, putative | A2D9M0 | Mrp, putative | ParA | 4.00E-029 |
| TVAG_217870 | Ind-3 (P-Loop ATPase) | nucleotide-binding protein, putative | A2F0N4 | Mrp protein, putative | ParA | 4.40E-014 |
| TVAG_257780 | HydG, Fe-hydrogenase assembly protein | Fe-hydrogenase assembly protein, putative | A2F4F9 | Fe-hydrogenase assembly protein, putative | BATS | 1.70E-015 |
| TVAG_296220 | Complex1, Tvh21 | NADH dehydrogenase 24 kDa subunit, putative | A2F771 | Respiratory-chain NADH dehydrogenase 24 Kd subunit family protein | Complex1_24kDa | 2.00E-034 |
| TVAG_133030 | Complex1, Tvh47 | NADH-ubiquinone oxidoreductase flavoprotein, putative | A2EDI1 | Hydrogenase chain, putative | Complex1_51K | 3.10E-038 |
| TVAG_113870 | Acetate:succinate CoA transferase-1 | hypothetical | A2DNP0 | Putative uncharacterized protein | AcetylCoA_hydro | 2.60E-030 |
| TVAG_164890 | Acetate:succinate CoA transferase-2 | Acetyl-CoA hydrolase, putative | A2FS74 | Putative uncharacterized protein | AcetylCoA_hydro | 2.90E-031 |
| TVAG_060450 | Acetyltransferase-1 | conserved hypothetical protein | A2ECH4 | Acetyltransferase, GNAT family protein | Acetyltransf_1 | 1.30E-006 |
| TVAG_270750 | Acetyltransferase-2 | protease synthase and sporulation negative regulatory protein PAI, putative | A2FIG7 | Acetyltransferase, GNAT family protein | Acetyltransf_1 | 7.20E-008 |
| TVAG_489800 | Adenylate kinase | NADH dehydrogenase 51 kDa subunit, putative | A2FQ69 | Adenylate kinase | ADK | 5.30E-028 |
| TVAG_003900 | Ferredoxin 1 | Ferredoxin 1 | A2E5A4 | Ferredoxin 1 | Fer2 | 2.50E-005 |
| TVAG_037570 | Iron hydrogenase 64kDa | NADH-ubiquinone oxidoreductase, putative | A2FCW4 | 64kDa iron hydrogenase, putative | Fe_hyd_lg_C | 0.00E+000 |
| TVAG_361590 | Iron hydrogenase 64kDa | 64kDa iron hydrogenase, putative | A2FQN6 | 64kDa iron hydrogenase, putative | Fe_hyd_lg_C | 0.00E+000 |
| TVAG_182620 | Iron hydrogenase (TvhydB) | nitrate, fromate, iron dehydrogenase, putative | A2D900 | TvhydB protein, putative | Fe_hyd_lg_C | 0.00E+000 |
| TVAG_310050 | Iron hydrogenase (TvhydB) | nitrate, fromate, iron dehydrogenase, putative | A2EKS1 | TvhydB protein, putative | Fe_hyd_lg_C | 0.00E+000 |
| TVAG_267870 | Malic enzyme A | malic enzyme, putative | A2DLD6 | Malic enzyme | Malic_M | 0.00E+000 |
| TVAG_238830 | Malic enzyme B | malic enzyme, putative | A2DGB5 | Malic enzyme | Malic_M | 0.00E+000 |
| TVAG_412220 | Malic enzyme D | malic enzyme, putative | A2F1M8 | Malic enzyme | Malic_M | 0.00E+000 |
| TVAG_340290 | Malic enzyme H | malic enzyme, putative | A2EKE3 | Malic enzyme | Malic_M | 0.00E+000 |
| TVAG_068130 | Malic enzyme I | malic enzyme, putative | A2EMJ7 | Malic enzyme | Malic_M | 0.00E+000 |
| TVAG_183790 | Malic enzyme (AP65-3) | malic enzyme, putative | A2D9B6 | Malic enzyme | Malic_M | 0.00E+000 |
| TVAG_198110 | Pyruvate:ferredoxin oxidoreductase A | pyruvate-flavodoxin oxidoreductase, putative | A2DDJ8 | Pyruvate:ferredoxin oxidoreductase A | POR_N | 2.30E-033 |
| TVAG_230580 | Pyruvate:ferredoxin oxidoreductase BI | pyruvate-flavodoxin oxidoreductase, putative | A2EDY6 | Pyruvate:ferredoxin oxidoreductase BI | POR_N | 1.10E-033 |
| TVAG_242960 | Pyruvate:ferredoxin oxidoreductase BII | pyruvate-flavodoxin oxidoreductase, putative | A2F855 | Pyruvate:ferredoxin oxidoreductase BII | POR_N | 5.00E-033 |
| TVAG_254890 | Pyruvate:ferredoxin oxidoreductase E | pyruvate-flavodoxin oxidoreductase, putative | A2EXN1 | Pyruvate:ferredoxin oxidoreductase E | POR_N | 1.80E-032 |
| TVAG_165340 | Succinyl-CoA synthase α-subunit | succinate thiokinase a subunit | A2DUL5 | Putative uncharacterized protein | Ligase_CoA | 2.00E-026 |
| TVAG_318670 | Succinyl-CoA syntahse α-subunit | succinate thiokinase a subunit | A2G5L7 | Adhesin protein AP33-1 | Ligase_CoA | 4.20E-026 |
| TVAG_144730 | Succinate thiokinase β subunit | succinate thiokinase ? subunit | A2FVK7 | Adhesin protein AP51-2, putative | ATP-grasp_2 | 1.40E-044 |
| TVAG_259190 | Succinate thiokinase β subunit | succinate thiokinase ? subunit | A2EBX0 | Succinyl-CoA ligase beta-chain, hydrogenosomal | ATP-grasp_2 | 8.40E-045 |
| TVAG_183500 | Succinate thiokinase β subunit | succinate thiokinase ? Subunit | A2D987 | Adhesin protein AP51-3, putative | ATP-grasp_2 | 2.80E-045 |
| TVAG_036010 | Flavodiiron protein | A-type flavoprotein | A2DAS3 | Hydrogenosomal oxygen reductase | Flavodoxin_1 | 1.80E-005 |
| TVAG_055200 | Thiol peroxidase (peroxiredoxin family) | peroxiredoxin, putative | A2ETI8 | Thiol peroxidase, putative | Redoxin | 1.30E-019 |
| TVAG_064490 | Rubrerythrin-1 | rubrerythrin, putative | A2EHC4 | Rubrerythrin, putative | Rubrerythrin | 7.80E-011 |
| TVAG_275660 | Rubrerythrin-2 | rubrerythrin, putative | A2EYD9 | Rubrerythrin, putative | Rubrerythrin | 4.10E-009 |
| TVAG_206500 | Hybrid-cluster protein-1 | Hydroxylamine reductase, putative | A2E1N5 | Hybrid-cluster protein, putative | Prismane | 0.00E+000 |
| TVAG_121620 | Oxidoreductase, FAD/FMN-binding family protein | N-ethylmaleimide reductase, putative | A2E973 | Oxidoreductase, FAD/FMN-binding family protein | Oxidored_FMN | 0.00E+000 |
| TVAG_088050 | Chaperonin 60, putative | chaperonin, putative | A2F5J7 | Chaperonin 60, putative | Cpn60_TCP1 | 0.00E+000 |
| TVAG_237140 | Mitochondrial-type HSP70, putative | heat shock protein, putative | A2DCR4 | Mitochondrial-type HSP70, putative | HSP70 | 0.00E+000 |
| TVAG_340390 | Mitochondrial-type HSP70, putative | heat shock protein 70 (HSP70)-4, putative | A2EKF3 | Mitochondrial-type HSP70, putative | HSP70 | 0.00E+000 |
| TVAG_433130 | Mitochondrial-type HSP70, putative | heat shock protein, putative | A2DIV4 | Heat shock 70 kDa protein, mitochondrial, putative | HSP70 | 0.00E+000 |
| TVAG_197980 | HSP20 | heat shock protein, putative | A2EJL4 | Hsp20/alpha crystallin family protein | HSP20 | 3.40E-013 |
| TVAG_287530 | HSP20 | heat shock protein, putative | A2FKF2 | Hsp20/alpha crystallin family protein | HSP20 | 8.40E-013 |
| TVAG_381290 | HSP20 | heat shock protein, putative | A2FKC5 | Hsp20/alpha crystallin family protein | HSP20 | 2.50E-011 |
| TVAG_119710 | Hydrogenosomal processing peptidase alpha-subunit | Clan ME, family M16, insulinase-like metallopeptidase | A2D7B7 | Clan ME, family M16, insulinase-like metallopeptidase | Peptidase_M16_C | 1.40E-001 |
| TVAG_233350 | Hydrogenosomal processing peptidase beta-subunit | Clan ME, family M16, insulinase-like metallopeptidase | A2ES04 | Clan ME, family M16, insulinase-like metallopeptidase | Peptidase_M16 | 6.60E-019 |
| TVAG_063000 | M24 aminopeptidase | hypothetical protein | A2DLR2 | Putative uncharacterized protein | DUF2404 | 2.90E-002 |
| TVAG_043720 | Serine peptidase | Clan SC, family S9, unassigned serine peptidase | A2EVB2 | Clan SC, family S9, unassigned serine peptidase | Peptidase_S15 | 8.10E-014 |
| TVAG_074600 | Aspartate aminotransferase | tyrosine aminotransferase, putative | A2E3X7 | Aminotransferase, classes I and II family protein | Aminotran_1_2 | 4.50E-031 |
| TVAG_088220 | Alanine aminotransferase-1 | aspartate aminotransferase, putative | A2FY82 | Aminotransferase, classes I and II family protein | Aminotran_1_2 | 9.30E-037 |
| TVAG_132440 | Alanine aminotransferase-2 | alanine aminotransferase, putative | A2GAH8 | Aminotransferase, classes I and II family protein | Aminotran_1_2 | 2.40E-033 |
| TVAG_183850 | Arginine deiminase-1 | conserved hypothetical protein | A2D9C2 | Amidinotransferase family protein | Amidinotransf | 0.00E+000 |
| TVAG_344520 | Arginine deiminase-2 | conserved hypothetical protein | A2FRG6 | Amidinotransferase family protein | Amidinotransf | 0.00E+000 |
| TVAG_379550 | Alanine aminotransferase | tyrosine aminotransferase, putative | A2E7I0 | Aminotransferase, classes I and II family protein | Aminotran_1_2 | 1.70E-033 |
| TVAG_177600 | Glycine cleavage H-protein | glycine cleavage system H protein, putative | A2G8D9 | Glycine cleavage H-protein | GCV_H | 9.30E-030 |
| TVAG_496160 | Phosphofructokinase family protein (Hexokinase)-1 | phosphofructokinase, putative | A2FR66 | Phosphofructokinase family protein | PFK | 0.00E+000 |
| TVAG_293770 | Phosphofructokinase family protein (Hexokinase)-2 | phosphofructokinase, putative | A2FKA7 | Phosphofructokinase family protein | PFK | 0.00E+000 |
| TVAG_462920 | Phosphofructokinase family protein (Hexokinase)-3 | phosphofructokinase, putative | A2DM03 | Phosphofructokinase family protein | PFK | 0.00E+000 |
| TVAG_321010 | AMP-binding enzyme family protein, long-chain acyl-CoA synthetase family protein | AMP dependent ligase/synthetase, putative | A2F809 | AMP-binding enzyme family protein | AMP-binding | 5.30E-033 |
| TVAG_075420 | Peroxiredoxin-1 | alkyl hydroperoxide reductase, subunit C,putative | A2FEE9 | Tryparedoxin peroxidase, putative | AhpC-TSA | 4.90E-017 |
| TVAG_095250 | Peroxiredoxin-2 | alkyl hydroperoxide reductase, subunit C, putative | A2G9K3 | Tryparedoxin peroxidase, putative | AhpC-TSA | 2.00E-016 |
| TVAG_455310 | Peroxiredoxin-3 | peroxiredoxins, prx-1, prx-2, prx-3, putative | A2GAU8 | Tryparedoxin peroxidase, putative | AhpC-TSA | 3.60E-017 |
| TVAG_350540 | Peroxiredoxin-4 | alkyl hydroperoxide reductase, subunit C, putative | A2ESJ9 | Thioredoxin peroxidase, putative | AhpC-TSA | 5.60E-015 |
| TVAG_528900 | Peroxiredoxin-5 | hypothetical | A2HQN8 | Putative uncharacterized protein (Fragment) | 1-cysPrx_C | 5.20E-004 |
| TVAG_152690 | NADH oxidase, FAD/FMN-binding family protein-1 | N-ethylmaleimide reductase, putative | A2FWL7 | Oxidoreductase, FAD/FMN-binding family protein | Oxidored_FMN | 0.00E+000 |
| TVAG_272880 | NADH oxidase, FAD/FMN-binding family protein-2 | NADH oxidase, putative | A2F0G8 | Oxidoreductase, FAD/FMN-binding family protein | Oxidored_FMN | 0.00E+000 |
| TVAG_351540 | NADH oxidase, FAD/FMN-binding family protein-3 | 2,4-dienoyl-CoA reductase [NADPH], putative | A2DZN8 | Oxidoreductase, FAD/FMN-binding family protein | Oxidored_FMN | 0.00E+000 |
| TVAG_044510 | Cytoplasmic HSP70, putative | heat shock protein 70 (HSP70)-4, putative | A2EY23 | Cytoplasmic heat shock protein 70, putative | HSP70 | 0.00E+000 |
| TVAG_381470 | Hsp70 dnaK-1 | heat shock protein 70 (HSP70)-4, putative | A2FKE2 | DnaK protein (Fragment) | HSP70 | 0.00E+000 |
| TVAG_479220 | Hsp70 dnaK-2 | heat shock protein, putative | A2FVR2 | DnaK protein | HSP70 | 0.00E+000 |
| TVAG_491770 | Hsp70 dnaK-3 | heat shock protein 70kD, putative | A2EAK8 | DnaK protein | HSP70 | 0.00E+000 |
| TVAG_125390 | CAMK family protein kinase | CAMK family protein kinase | A2F9L4 | CAMK family protein kinase | Pkinase | 1.70E-041 |
| TVAG_132350 | Protein kinase, putative | conserved hypothetical protein | A2FNL7 | Putative uncharacterized protein | Pkinase_Tyr | 4.10E-009 |
| TVAG_165530 | STE family protein kinase | STE family protein kinase | A2DUN4 | STE family protein kinase | Pkinase | 2.30E-041 |
| TVAG_228770 | TKL family protein kinase | TKL family protein kinase | A2DJ39 | TKL family protein kinase | Pkinase_Tyr | 1.70E-040 |
| TVAG_433790 | Metallopeptidase, putative | Clan MH, family M20, peptidase T-like metallopeptidase | A2F7S4 | Clan MH, family M20, peptidase T-like metallopeptidase | M20_dimer | 1.30E-008 |
| TVAG_074970 | Serine peptidase, putative | Clan SC, family S33, methylesterase-like serine peptidase | A2E414 | Clan SC, family S33, methylesterase-like serine peptidase | Peptidase_S9 | 1.00E-010 |
| TVAG_082020 | Peptidase, putative | conserved hypothetical protein | A2GHT5 | Putative uncharacterized protein (Fragment) | Peptidase_M28 | 1.80E-006 |
| TVAG_054030 | Actin | actin, putative | A2G1K5 | Actin | Actin | 0.00E+000 |
| TVAG_215920 | Laminin | conserved hypothetical protein | A2FRW1 | Laminin A family protein | Phospholip_B | 0.00E+000 |
| TVAG_026390 | Histone H2B, putative | histone H2b, putative | A2DJ26 | Histone H2B, putative | Histone | 1.40E-006 |
| TVAG_248450 | peptidyl-tRNA hydrolase, putative | peptidyl-tRNA hydrolase, putative | A2E781 | Putative uncharacterized protein | PTH2 | 6.20E-026 |
| TVAG_473170 | EF hand family protein | calcium binding protein, putative | A2ERW3 | EF hand family protein | SPARC_Ca_bdg | 1.20E-003 |
| TVAG_273260 | Alpha-amylase, putative | alpha-amylase, putative | A2EZV2 | Alpha amylase, catalytic domain containing protein | Alpha-amylase | 6.10E-029 |
| TVAG_043500 | Enolase | enolase, putative | A2EV90 | Enolase (EC 4.2.1.11) | Enolase_C | 0.00E+000 |
| TVAG_397250 | Glucokinase | glucokinase, putative | A2DXB2 | Putative uncharacterized protein | Glucokinase | 3.20E-031 |
| TVAG_146910 | Glyceraldehyde 3-phosphate dehydrogenase | glyceraldehyde 3-phosphate dehydrogenase, putative | A2DHT2 | Glyceraldehyde 3-phosphate dehydrogenase (EC 1.2.1.12) | Gp_dh_C | 0.00E+000 |
| TVAG_043060 | Fructose-bisphosphate aldolase-1 | fructose-bisphosphate aldolase, putative | A2F6S0 | Fructose-bisphosphate aldolase (EC 4.1.2.13) | F_bP_aldolase | 0.00E+000 |
| TVAG_271850 | Unknown (Sel1 domain) | conserved hypothetical protein | A2E5T2 | Cec-1, putative | DUF965 | 1.00E+000 |
| TVAG_172700 | TPR Domain containing protein | anaphase promoting complex subunit, putative | A2DF21 | TPR Domain containing protein | Coatomer_E | 1.10E-001 |
| TVAG_283360 | TPR Domain containing protein | hypothetical | A2DEN9 | Putative uncharacterized protein | ChAPs | 8.10E-003 |
| TVAG_102720 | TPR Domain containing protein | hypothetical | A2ECX8 | Putative uncharacterized protein | DUF687 | 3.70E+000 |
| TVAG_315350 | TPR Domain containing protein | chaperone binding protein, putative | A2FDK4 | TPR Domain containing protein | ChAPs | 3.70E-004 |
| TVAG_317590 | Unknown (TPR Domain) | hypothetical | A2FTK8 | Putative uncharacterized protein | CPSF_A | 2.30E-015 |
| TVAG_019240 | Ubiquitin | ubiquitin, putative | A2DC67 | Polyubiquitin, putative | Ribosomal_L40e | 7.30E-017 |
| TVAG_064150 | ADP-ribosylation factor, putative | ADP-ribosylation factor, arf, putative | A2EIX2 | ADP-ribosylation factor, putative | Arf | 0.00E+000 |
| TVAG_137880 | Cyclophilin superfamily (Peptidyl-prolyl cis-trans isomerase) | peptidyl-prolyl cis-trans isomerase A, ppia, putative | A2EC21 | Peptidyl-prolyl cis-trans isomerase (EC 5.2.1.8) | Pro_isomerase | 4.70E-035 |
| TVAG_045010 | Glucokinase, putative | glucokinase, putative | A2E8E9 | Putative uncharacterized protein | Glucokinase | 2.40E-027 |
| TVAG_219820 | Conserve Unknown protein | conserved hypothetical protein | A2DXT9 | Putative uncharacterized protein | HOOK | 1.40E-001 |
| TVAG_234440 | Ubiquitin ligase, putative | ubiquitin-protein ligase E3a, putative | A2FPQ0 | Ubiquitin ligase, putative | HECT | 0.00E+000 |
| TVAG_075320 | vacuolar proton ATPase, putative | vacuolar proton ATPase, putative | A2FED9 | V-type ATPase 116kDa subunit family protein | V_ATPase_I | 0.00E+000 |
| TVAG_030480 | Rhodanese-like domain containing protein | Phage shock protein E precursor, putative | A2EY87 | Rhodanese-like domain containing protein | Rhodanese | 1.70E-008 |
| TVAG_311860 | Rhodanese-related sulfurtransferase-like protein | conserved hypothetical protein | A2EJZ2 | Putative uncharacterized protein | Rhodanese | 3.40E-006 |
| TVAG_118780 | Calmoduline-putative | calmodulin, putative | A2EAY1 | EF hand family protein | SPARC_Ca_bdg | 7.50E-004 |
| TVAG_026290 | Oxysterol-binding protein, putative | oxysterol-binding protein, putative | A2DZ32 | Putative uncharacterized protein | Oxysterol_BP | 0.00E+000 |
| TVAG_229870 | dihydrofolate synthase/folylpolyglutamate synthase, putative | dihydrofolate synthase/folylpolyglutamate synthase, putative | A2G107 | FolC bifunctional protein | Mur_ligase_M | 1.30E-012 |
| TVAG_277050 | Citrate lyase beta chain, putative | Citrate lyase beta chain, putative | A2FP65 | Citrate lyase beta like, putative | HpcH_HpaI | 1.80E-033 |
| TVAG_321030 | CoA binding domain containing protein, long chain Acyl-CoA synthetase family protein | conserved hypothetical protein | A2F811 | CoA binding domain containing protein | CoA_binding | 2.00E-005 |
| TVAG_342900 | NAD dependent epimerase/dehydratase, putative/Isoflavone reductase | NAD dependent epimerase/dehydratase, putative | A2EJQ2 | Isoflavone reductase, putative | NmrA | 8.70E-020 |
| TVAG_367660 | ABC transporter, putative | ABC transporter, putative | A2F5S0 | Putative uncharacterized protein | DUF3584 | 4.80E-003 |
| TVAG_239840 | MFS transporter | sucrose transport protein, putative | A2EFC7 | Major Facilitator Superfamily protein | MFS_1 | 8.60E-009 |
| TVAG_022120 | Unknown | conserved hypothetical protein | A2FFT0 | Putative uncharacterized protein | HSP70 | 2.50E-001 |
| TVAG_026100 | Unknown | conserved hypothetical protein | A2DZ13 | Putative uncharacterized protein | Phage_glycop_gL | 3.70E+000 |
| TVAG_028050 | Unknown | conserved hypothetical protein | A2E544 | Putative uncharacterized protein | DUF412 | 3.50E+000 |
| TVAG_038870 | Unknown | conserved hypothetical protein | A2E5J9 | Putative uncharacterized protein | TraS | 2.00E+000 |
| TVAG_044000 | Unknown | conserved hypothetical protein | A2E0G0 | Putative uncharacterized protein | Porin_3 | 2.90E+000 |
| TVAG_067030 | Unknown | conserved hypothetical protein | A2DSB9 | Putative uncharacterized protein | Porin_3 | 1.10E+000 |
| TVAG_074260 | Unknown | hypothetical | A2FRC3 | Putative uncharacterized protein | HOOK | 2.40E-001 |
| TVAG_089110 | Unknown | hypothetical | A2G4R1 | Putative uncharacterized protein | Adeno_terminal | 2.60E+000 |
| TVAG_090740 | Unknown | hypothetical | A2F911 | Putative uncharacterized protein | DUF2829 | 2.00E+000 |
| TVAG_102740 | Unknown | conserved hypothetical protein | A2ECY0 | Putative uncharacterized protein | AATase | 2.20E+000 |
| TVAG_103110 | Unknown | conserved hypothetical protein | A2EKM2 | Putative uncharacterized protein | VRP3 | 3.40E+000 |
| TVAG_104680 | Unknown | conserved hypothetical protein | A2FNU2 | Putative uncharacterized protein | DAG1 | 3.10E-003 |
| TVAG_113880 | Unknown | conserved hypothetical protein | A2DNP1 | Putative uncharacterized protein | ABC1 | 7.30E-001 |
| TVAG_140620 | Unknown | conserved hypothetical protein | A2EJV0 | Putative uncharacterized protein | zf-C4_C | 1.30E+000 |
| TVAG_165290 | Unknown | conserved hypothetical protein | A2DUL0 | Putative uncharacterized protein | DUF3303 | 3.20E-001 |
| TVAG_165320 | Unknown | conserved hypothetical protein | A2DUL3 | Putative uncharacterized protein | RXT2_N | 1.20E+000 |
| TVAG_178820 | Unknown | hypothetical | A2FR53 | Putative uncharacterized protein | Pox_A32 | 1.90E+001 |
| TVAG_197920 | Unknown | conserved hypothetical protein | A2EJK8 | Putative uncharacterized protein | UDP-g_GGTase | 7.00E-003 |
| TVAG_209310 | Unknown | conserved hypothetical protein | A2DVH3 | Putative uncharacterized protein | AATase | 4.60E-001 |
| TVAG_225570 | Unknown | conserved hypothetical protein | A2DNT6 | Putative uncharacterized protein | DUF1135 | 5.90E-001 |
| TVAG_225930 | Unknown | conserved hypothetical protein | A2DNX2 | Putative uncharacterized protein | Fucose_iso_C | 1.10E-003 |
| TVAG_237550 | Unknown | conserved hypothetical protein | A2DCV5 | Putative uncharacterized protein | U-box | 8.40E-011 |
| TVAG_241570 | Unknown | conserved hypothetical protein | A2FL05 | Putative uncharacterized protein | CPT | 1.40E-003 |
| TVAG_249920 | Unknown | hypothetical protein | A2DCJ5 | Putative uncharacterized protein | DUF791 | 5.80E-001 |
| TVAG_301190 | Unknown | hypothetical | A2E5E1 | Putative uncharacterized protein | DUF2153 | 1.20E+001 |
| TVAG_321550 | Unknown | hypothetical | A2FXX9 | Putative uncharacterized protein | SipA | 2.50E+000 |
| TVAG_334260 | Unknown | conserved hypothetical protein | A2EID2 | Putative uncharacterized protein | dsRNA_bind | 8.50E+000 |
| TVAG_343040 | Unknown | conserved hypothetical protein | A2EJR6 | Putative uncharacterized protein | DUF3081 | 6.10E+000 |
| TVAG_392650 | Unknown | conserved hypothetical protein | A2DWW6 | Putative uncharacterized protein | DUF2419 | 9.00E-001 |
| TVAG_415360 | Unknown | hypothetical | A2EW66 | Putative uncharacterized protein | PAT1 | 2.50E-004 |
| TVAG_416630 | Unknown | conserved hypothetical protein | A2EQQ5 | Putative uncharacterized protein | DUF2205 | 8.60E-001 |
| TVAG_430700 | Unknown | hypothetical | A2E395 | Putative uncharacterized protein | Alpha-2-MRAP_N | 2.30E+000 |
| TVAG_431100 | Unknown | hypothetical | A2EZE8 | Putative uncharacterized protein | Myosin_tail_1 | 1.70E-004 |
| TVAG_433120 | Unknown | hypothetical | A2DIV3 | Putative uncharacterized protein | eIF2A | 6.30E-003 |
| TVAG_437350 | Unknown | hypothetical | A2DFI1 | Putative uncharacterized protein | DUF1365 | 1.80E+000 |
| TVAG_442170 | Unknown | conserved hypothetical protein | A2F1W1 | Putative uncharacterized protein | Nup84_Nup100 | 4.90E+000 |
| TVAG_445430 | Unknown | hypothetical | A2E4K9 | Putative uncharacterized protein | FERM_M | 2.20E-009 |
| TVAG_450060 | Unknown | conserved hypothetical protein | A2G998 | Putative uncharacterized protein | ABC1 | 6.60E-001 |
| TVAG_454330 | Unknown | conserved hypothetical protein | A2EU46 | Putative uncharacterized protein | DRIM | 2.40E+000 |
| TVAG_483050 | Unknown | hypothetical | A2FAK5 | Putative uncharacterized protein | MAD | 7.10E-003 |
| TVAG_483980 | Unknown | conserved hypothetical protein | A2EA41 | Putative uncharacterized protein | GcpE | 6.20E-001 |
| TVAG_493810 | Unknown | conserved hypothetical protein | A2DQ12 | Putative uncharacterized protein | Pox_Rap94 | 3.00E+000 |
| TVAG_521380 | Unknown | conserved hypothetical protein | A2HW55 | Putative uncharacterized protein | Phytochelatin_C | 3.10E+000 |
| TVAG_547420 | Unknown | conserved hypothetical protein | A2H7Y7 | Putative uncharacterized protein (Fragment) | rpo30_N | 1.20E+000 |
| TVAG_607400 | Unknown | hypothetical | A2HD51 | Putative uncharacterized protein (Fragment) | TMP | 2.50E-001 |
