# Supplementary material for: The Core Components of Organelle Biogenesis and Membrane Transport in the Hydrogenosomes of Trichomonas vaginalis
Source: PLoS One. 2011 Sep 15;6(9):e24428. doi: 10.1371/journal.pone.0024428 (PMC3174187; doi:10.1371/journal.pone.0024428)
Supplement: Table S3 — Putative matrix proteins identified in T. vaginalis hydrogenosome. (DOC) [file pone.0024428.s013.doc]

**Table S3A. Putative matrix proteins identified in *T. vaginalis*** hydrogenosome.

| **Identification** | | **Structure** | | **Cell localization** | | | **Signal** |
| --- | --- | --- | --- | --- | --- | --- | --- |
| Accession number | Name | TMHMM | MEMSAT3 | TargetP | PsortII | Exp. Local. |  |
|  |  | TM No. | TM No. |  | mit% |  |  |
| **Iron-sulfur assembly** | | | | | | | |
| TVAG_239660 | IscS-2, cystein desulfurase | 0 | 0 | O | 13.0 % | H^[1]^ |  |
| TVAG_432650 | IscU | 0 | 0 | M | 30.4 % |  | Δ |
| TVAG_008840 | Nfu-2 | 0 | 0 | M | 39.1 % | [H](../../../../C:%5CDocuments%20and%20Settings%5Cvsichni%5CDokumenty%5CMy%20Dropbox%5CRada%5CTachezy%5CPaper%5CTachezy%20Trichomonas%20paper%5CTabulka%20+obrázky%5CObrázky%5CTvNfu.tif) | Δ |
| TVAG_456770 | IscA2-1 | 0 | 0 | O | 56.5 % | [H](../../../../C:%5CDocuments%20and%20Settings%5Cvsichni%5CDokumenty%5CMy%20Dropbox%5CRada%5CTachezy%5CPaper%5CTachezy%20Trichomonas%20paper%5CTabulka%20+obrázky%5CObrázky%5CTvHesB.tif) | Δ |
| TVAG_027170 | Ind-1 (P-Loop ATPase) | 0 | 0 | M | 43.5 % | [H](../../../../C:%5CDocuments%20and%20Settings%5Cvsichni%5CDokumenty%5CMy%20Dropbox%5CRada%5CTachezy%5CPaper%5CTachezy%20Trichomonas%20paper%5CTabulka%20+obrázky%5CObrázky%5CTvInd1.tif) | Δ |
| TVAG_076230 | Ind-2 (P-loop ATPase) | 0 | 0 | M | 21.7 % |  | Δ |
| TVAG_217870 | Ind-3 (P-Loop ATPase) | 0 | 0 | O | 34.8 % |  | Δ |
| TVAG_257780 | HydG, Fe-hydrogenase assembly protein | 0 | 0 | M | 21.7 % | H^[2]^ | Δ |
| **Pyruvate/malate metabolism** | | | | | | | |
| TVAG_296220 | Complex1, Tvh21 | 0 | 0 | M | 17.4 % | H^[3]^ | Δ |
| TVAG_133030 | Complex1, Tvh47 | 0 | 0 | O | * | H^[3]^ | Δ |
| TVAG_113870 | Acetate:succinate CoA transferase-1 | 0 | 0 | M | 47.8 % |  | Δ |
| TVAG_164890 | Acetate:succinate CoA transferase-2 | 0 | 0 | O | 56.5 % |  |  |
| TVAG_060450 | Acetyltransferase-1 | 0 | 0 | O | 26.1 % |  | Δ |
| TVAG_270750 | Acetyltransferase-2 | 0 | 0 | O | 30.4 % |  | Δ |
| TVAG_489800 | Adenylate kinase | 0 | 0 | M | 8.7 % |  | Δ |
| TVAG_003900 | Ferredoxin 1 | 0 | 0 | O | * | H^[4]^ | Δ |
| TVAG_037570 | Iron hydrogenase 64kDa | 0 | 0 | O | 26.1 % |  | Δ |
| TVAG_361590 | Iron hydrogenase 64kDa | 0 | 0 | O | 30.4 % |  | Δ |
| TVAG_182620 | Iron hydrogenase (TvhydB) | 0 | 0 | O | 26.1 % |  | Δ |
| TVAG_310050 | Iron hydrogenase (TvhydB) | 0 | 0 | O | 17.4 % |  |  |
| TVAG_267870 | Malic enzyme A | 0 | 0 | O | 43.5 % | H^[5]^ | Δ |
| TVAG_238830 | Malic enzyme B | 0 | 0 | M | 8.7 % |  | Δ |
| TVAG_412220 | Malic enzyme D | 0 | 0 | O | 39.1 % |  | Δ |
| TVAG_340290 | Malic enzyme H | 0 | 0 | O | 13.0 % |  | Δ |
| TVAG_068130 | Malic enzyme I | 0 | 0 | O | 13.0 % |  |  |
| TVAG_183790 | Malic enzyme (AP65-3) | 0 | 0 | O | 26.1 % | H^[6]^ | Δ |
| TVAG_198110 | Pyruvate:ferredoxin oxidoreductase A | 0 | 2 | O | 26.1 % | H | Δ |
| TVAG_230580 | Pyruvate:ferredoxin oxidoreductase BI | 0 | 2 | M | 34.8 % |  | Δ |
| TVAG_242960 | Pyruvate:ferredoxin oxidoreductase BII | 0 | 2 | M | 21.7 % |  | Δ |
| TVAG_254890 | Pyruvate:ferredoxin oxidoreductase E | 0 | 2 | M | 17.4 % |  | Δ |

**Table S3B. Putative matrix proteins identified in *T. vaginalis*** hydrogenosome.

| **Identification** | | **Structure** | | **Cell localization** | | | **Signal** |
| --- | --- | --- | --- | --- | --- | --- | --- |
| Accession number | Name | TMHMM | MEMSAT3 | TargetP | PsortII | Exp. Local. |  |
|  |  | TM No. | TM No. |  | mit% |  |  |
| **Pyruvate/malate metabolism** | | | | | | | |
| TVAG_165340 | Succinyl-CoA synthase α-subunit | 0 | 0 | O | 13.0 % | H^[7]^ | Δ |
| TVAG_318670 | Succinyl-CoA syntahse α-subunit | 0 | 0 | O | 8.7 % |  | Δ |
| TVAG_144730 | Succinyl-CoA syntahse β -subunit | 0 | 0 | O | * | H^[8]^ | Δ |
| TVAG_259190 | Succinyl-CoA syntahse β -subunit | 0 | 0 | O | 8.7 % |  | Δ |
| TVAG_183500 | Succinyl-CoA syntahse β -subunit | 0 | 0 | O | 4.3 % |  | Δ |
| **Oxygen metabolism** | | | | | | | |
| TVAG_036010 | Flavodiiron protein | 0 | 0 | O | 4.3 % | H^[9]^ | Δ |
| TVAG_055200 | Thiol peroxidase (peroxiredoxin family) | 0 | 0 | O | 13.0 % | H^[10]^ |  |
| TVAG_064490 | Rubrerythrin-1 | 0 | 0 | O | 8.7 % | H^[10]^ | Δ |
| TVAG_275660 | Rubrerythrin-2 | 0 | 0 | O | * |  |  |
| TVAG_206500 | Hybrid-cluster protein-1 | 0 | 0 | O | 13.0 % | [H](../../../../C:%5CDocuments%20and%20Settings%5Cvsichni%5CDokumenty%5CMy%20Dropbox%5CRada%5CTachezy%5CPaper%5CTachezy%20Trichomonas%20paper%5CTabulka%20+obrázky%5CObrázky%5CTvHCP.tif) |  |
| TVAG_121620 | Oxidoreductase, FAD/FMN-binding family protein | 0 | 0 | M | 34.8 % |  |  |
| **Heat shock proteins** | | | | | | | |
| TVAG_088050 | Chaperonin 60, putative | 0 | 0 | M | 39.1 % |  | Δ |
| TVAG_237140 | Mitochondrial-type HSP70 | 0 | 0 | M | 39.1 % |  | Δ |
| TVAG_340390 | Mitochondrial-type HSP70 | 0 | 0 | M | 26.1 % |  | Δ |
| TVAG_433130 | Mitochondrial-type HSP70 | 0 | 0 | M | 21.7 % |  | Δ |
| TVAG_197980 | HSP20 | 0 | 0 | O | 17.4 % |  |  |
| TVAG_287530 | HSP20 | 0 | 0 | O | 17.4 % |  |  |
| TVAG_381290 | HSP20 | 0 | 0 | O | 11.1 % |  | Δ |
| **Peptidases** | | | | | | | |
| TVAG_119710 | Hydrogenosomal processing peptidase alpha-subunit | 0 | 0 | O | 21.7 % | H^[11]^ |  |
| TVAG_233350 | Hydrogenosomal processing peptidase beta-subunit | 0 | 0 | O | 47.8 % | H^[11]^ |  |
| TVAG_063000 | M24 aminopeptidase | 0 | 0 | O | * | [H](../../../../C:%5CDocuments%20and%20Settings%5Cvsichni%5CDokumenty%5CMy%20Dropbox%5CRada%5CTachezy%5CPaper%5CTachezy%20Trichomonas%20paper%5CTabulka%20+obrázky%5CObrázky%5CMdm-Mmm.tif) | Δ |
| TVAG_043720 | Serine peptidase | 0 | 0 | M | 34.8 % |  |  |

**Table S3C. Putative matrix proteins identified in *T. vaginalis*** hydrogenosome.

| **Identification** | | **Structure** | | **Cell localization** | | | **Signal** |
| --- | --- | --- | --- | --- | --- | --- | --- |
| Accession number | Name | TMHMM | MEMSAT3 | TargetP | PsortII | Exp. Local. |  |
|  |  | TM No. | TM No. |  | mit% |  |  |
| **Aminotransferases** | | | | | | | |
| TVAG_074600 | Aspartate aminotransferase | 0 | 0 | M | 30.4 % |  |  |
| TVAG_088220 | Alanine aminotransferase-1 | 0 | 0 | M | 52.2 % | [H](../../../../C:%5CDocuments%20and%20Settings%5Cvsichni%5CDokumenty%5CMy%20Dropbox%5CRada%5CTachezy%5CPaper%5CTachezy%20Trichomonas%20paper%5CTabulka%20+obrázky%5CObrázky%5CAlanine.tif) |  |
| TVAG_132440 | Alanine aminotransferase-2 | 0 | 0 | M | 47.8 % |  | Δ |
| TVAG_183850 | Arginine deiminase-1 | 0 | 0 | M | 65.2 % |  | Δ |
| TVAG_344520 | Arginine deiminase-2 | 0 | 0 | M | 56.5 % |  |  |
| TVAG_379550 | Alanine aminotransferase | 0 | 0 | M | 87.0 % |  | Δ |
| TVAG_177600 | Glycine cleavage H-protein | 0 | 0 | O | 34.8 % | H^[12]^ |  |
| **Carbohydrate and lipid metabolism** | | | | | | | |
| TVAG_496160 | Phosphofructokinase-1 | 0 | 0 | M | 13.0 % |  |  |
| TVAG_293770 | Phosphofructokinase-2 | 0 | 0 | O | 13.0 % | [H](../../../../C:%5CDocuments%20and%20Settings%5Cvsichni%5CDokumenty%5CMy%20Dropbox%5CRada%5CTachezy%5CPaper%5CTachezy%20Trichomonas%20paper%5CTabulka%20+obrázky%5CObrázky%5CTvPFKhy.tif) | Δ |
| TVAG_462920 | Phosphofructokinase-3 | 0 | 0 | O | 11.1 % |  |  |
| TVAG_321010 | AMP-binding enzyme family protein, long-chain  acyl-CoA synthetase family protein | 0 | 0 | O | 21.7 % |  |  |

Proteins were manually annotated based on searches in TrichDB, Uniprot, and PFAM A+B (Table S2). Protein structure was predicted using TMHMM and MEMSAT3; subcellular location was predicted using TargetP and PsortII. TM No., number of predicted transmembrane α-helixes. M, predicted location in mitochondria; O, predicted location in other compartments; Mit%, probability percentage of mitochondrial location; *, mitochondrial location was not predicted; Exp. Local., experimental location; H, localization of HA-tagged proteins was confirmed in *T. vaginalis* hydrogenosomes by immunofluorescence microscopy; NV, transformed *T. vaginalis* strain was not viable. Signal: Δ indicates N-terminal targeting sequence identified by Hunter.

**References**

1. Tachezy J, Sánchez LB, Müller M (2001) Mitochondrial type iron-sulfur cluster assembly in the amitochondriate eukaryotes *Trichomonas vaginalis* and *Giardia intestinalis*, as indicated by the phylogeny of IscS. Mol Biol Evol. 18 :1919-28.
2. Pütz S, Dolezal P, Gelius-Dietrich G, Bohacova L, Tachezy J, Henze K (2006) Fe-hydrogenase maturases in the hydrogenosomes of *Trichomonas vaginalis*. Eukaryot Cell 5: 579-86.
3. Hrdy I, Hirt RP, Dolezal P, Bardonová L, Foster PG, Tachezy J, Embley TM (2004) Trichomonas hydrogenosomes contain the NADH dehydrogenase module of mitochondrial complex I. Nature 432: 618-22.
4. Hrdy I, Müller M (1995) Primary structure and eubacterial relationships of the pyruvate:ferredoxin oxidoreductase of the amitochondriate eukaryote *Trichomonas vaginalis*. J Mol Evol 41 :388-96.
5. Hrdy I, Müller M (1995) Primary structure of the hydrogenosomal malic enzyme of *Trichomonas vaginalis* and its relationship to homologous enzymes. J Eukaryot Microbiol 42 :593-603.
6. O'Brien JL, Lauriano CM, Alderete JF (1996) Molecular characterization of a third malic enzyme-like AP65 adhesin gene of *Trichomonas vaginalis*. Microb Pathog 20 :335-49.
7. Lahti CJ, Bradley PJ, Johnson PJ (1994) Molecular characterization of the alpha-subunit of *Trichomonas vaginalis* hydrogenosomal succinyl CoA synthetase. Mol Biochem Parasitol 66: 309-18.
8. Lahti CJ, d'Oliveira CE, Johnson PJ (1992) Beta-succinyl-coenzyme A synthetase from *Trichomonas vaginalis* is a soluble hydrogenosomal protein with an amino-terminal sequence that resembles mitochondrial presequences. J Bacteriol 174: 6822-30.
9. Smutná T, Gonçalves VL, Saraiva LM, Tachezy J, Teixeira M, Hrdy I (2009) Flavodiiron protein from *Trichomonas vaginalis* hydrogenosomes: the terminal oxygen reductase. Eukaryot Cell 8: 47-55.
10. Pütz S, Gelius-Dietrich G, Piotrowski M, Henze K (2005) Rubrerythrin and peroxiredoxin: two novel putative peroxidases in the hydrogenosomes of the microaerophilic protozoon *Trichomonas vaginalis*. Mol Biochem Parasitol (2005) 142 :212-23.
11. Brown MT, Goldstone HM, Bastida-Corcuera F, Delgadillo-Correa MG, McArthur AG, Johnson PJ (2007) A functionally divergent hydrogenosomal peptidase with protomitochondrial ancestry. Mol Microbiol 64 :1154-63.
12. Mukherjee M, Brown MT, McArthur AG, Johnson PJ (2006) Proteins of the glycine decarboxylase complex in the hydrogenosome of *Trichomonas vaginalis*. Eukaryot Cell 5 :2062-71.
