# Supplementary material for: The Core Components of Organelle Biogenesis and Membrane Transport in the Hydrogenosomes of Trichomonas vaginalis
Source: PLoS One. 2011 Sep 15;6(9):e24428. doi: 10.1371/journal.pone.0024428 (PMC3174187; doi:10.1371/journal.pone.0024428)
Supplement: Table S4 — List of putative non-hydrogenosomal proteins. (DOC) [file pone.0024428.s014.doc]

**Table S4A. List of putative** non-hydrogenosomal proteins.

| **Identification** | | **Structure** | | **Cell localization** | | | **Signal** |
| --- | --- | --- | --- | --- | --- | --- | --- |
| Accession number | Name | TMHMM | MEMSAT3 | TargetP | PsortII | Exp. Local. |  |
|  |  | TM No. | TM No. |  | mit% |  |  |
| **Oxygen metabolism** | | | | | | | |
| TVAG_075420 | Peroxiredoxin-1 | 0 | 0 | O | 4.3 % |  |  |
| TVAG_095250 | Peroxiredoxin-2 | 0 | 0 | O | 13.0 % |  |  |
| TVAG_455310 | Peroxiredoxin-3 | 0 | 0 | O | 13.0 % |  |  |
| TVAG_350540 | Peroxiredoxin-4 | 0 | 0 | O | 4.3 % |  |  |
| TVAG_528900 | Peroxiredoxin-5 | 0 | 0 | O | 8.7 % |  |  |
| TVAG_152690 | NADH oxidase, FAD/FMN-binding family protein-1 | 0 | 0 | O | 26.1 % |  |  |
| TVAG_272880 | NADH oxidase, FAD/FMN-binding family protein-2 | 0 | 0 | O | 39.1 % |  |  |
| TVAG_351540 | NADH oxidase, FAD/FMN-binding family protein-3 | 0 | 0 | O | 34.8 % |  |  |
| **Heat shock proteins** | | | | | | | |
| TVAG_044510 | Cytoplasmic HSP70, putative | 0 | 0 | O | 13.0 % |  |  |
| TVAG_381470 | Hsp70 dnaK-1 | 0 | 0 | O | 17.4 % |  |  |
| TVAG_479220 | Hsp70 dnaK-2 | 0 | 0 | O | * |  |  |
| TVAG_491770 | Hsp70 dnaK-3 | 0 | 0 | O | 13.0 % |  |  |
| **Kinases** | | | | | | | |
| [TVAG_125390](http://www.trichdb.org/trichdb/showRecord.do?name=GeneRecordClasses.GeneRecordClass&project_id=TrichDB&primary_key=TVAG_125390) | CAMK family protein kinase | 0 | 0 | M | 47.8 % |  |  |
| TVAG_132350 | Protein kinase, putative | 0 | 0 | O | 0 |  |  |
| TVAG_165530 | STE family protein kinase | 0 | 0 | O | 17.4 % |  |  |
| TVAG_228770 | TKL family protein kinase | 0 | 0 | O | * |  |  |
| **Peptidases** | | | | | | |  |
| TVAG_433790 | Metallopeptidase, putative | 0 | 0 | O | 17.4 % |  |  |
| TVAG_074970 | Serine peptidase, putative | 0 | 0 | O | 13.0 % |  |  |
| TVAG_082020 | Peptidase, putative | 3 | 2 | O | 33.3 % |  |  |
| **Cytoskeleton** | | | | | | | |
| TVAG_054030 | Actin | 0 | 0 | O | * |  |  |
| TVAG_215920 | Laminin | 1 | 1 | M | 13.0 % |  | Δ / Ct |
| **DNA/RNA metabolism** | | | | | | |  |
| TVAG_026390 | Histone H2B, putative | 0 | 0 | O | 30.4 % |  |  |
| TVAG_248450 | peptidyl-tRNA hydrolase, putative | 1 | 0 | S | 13.0 % |  |  |
| TVAG_473170 | EF hand family protein | 0 | 0 | O | 11.1 % |  |  |

**Table S4B. Identified putative** non-hydrogenosomal proteins.

| **Identification** | | **Structure** | | **Cell localization** | | | **Signal** |
| --- | --- | --- | --- | --- | --- | --- | --- |
| Accession number | Name | TMHMM | MEMSAT3 | TargetP | PsortII | Exp. Local. |  |
|  |  | TM No. | TM No. |  | mit% |  |  |
| **Carbohydrate metabolism** | | | | | | | |
| TVAG_273260 | Alpha-amylase, putative | 0 | 0 | O | 8.7 % |  |  |
| TVAG_043500 | Enolase | 0 | 0 | O | 8.7 % |  |  |
| TVAG_397250 | Glucokinase | 0 | 0 | O | 4.3 % |  |  |
| TVAG_146910 | Glyceraldehyde 3-phosphate dehydrogenase | 0 | 0 | M | 43.5 % | [O](../../../../C:%5CDocuments%20and%20Settings%5Cvsichni%5CDokumenty%5CMy%20Dropbox%5CRada%5CTachezy%5CPaper%5CTachezy%20Trichomonas%20paper%5CTabulka%20+obrázky%5CObrázky%5CTvG3Pdehydrogenase.tif) |  |
| TVAG_043060 | Fructose-bisphosphate aldolase-1 | 0 | 0 | O | 17.4 % | [O](../../../../C:%5CDocuments%20and%20Settings%5Cvsichni%5CDokumenty%5CMy%20Dropbox%5CRada%5CTachezy%5CPaper%5CTachezy%20Trichomonas%20paper%5CTabulka%20+obrázky%5CObrázky%5CTvAldolase.tif) |  |
| **Miscellaneous** |  |  |  |  |  |  |  |
| TVAG_271850 | Unknown (Sel1 domain) | 0 | 0 | O | * |  |  |
| TVAG_172700 | TPR Domain containing protein | 0 | 0 | O | 17.4 % |  |  |
| TVAG_283360 | TPR Domain containing protein | 0 | 0 | O | 4.3 % |  |  |
| TVAG_102720 | TPR Domain containing protein | 0 | 1 | O | 8.7 % |  |  |
| TVAG_315350 | TPR Domain containing protein | 0 | 0 | O | 8.7 % | O |  |
| TVAG_317590 | TPR Domain containing protein | 0 | 1 | O | * |  |  |
| TVAG_019240 | Ubiquitin | 0 | 0 | O | 8.7 % |  |  |
| TVAG_064150 | ADP-ribosylation factor, putative | 0 | 0 | O | 8.7 % |  |  |
| TVAG_137880 | Cyclophilin superfamily (Peptidyl-prolyl cis-trans isomerase) | 0 | 0 | O | 4.3 % |  |  |
| TVAG_045010 | Glucokinase, putative | 0 | 0 | O | 4.3 % |  |  |
| TVAG_219820 | Conserve Unknown protein | 0 | 0 | O | 4.3 % |  |  |
| TVAG_234440 | Ubiquitin ligase, putative | 0 | 0 | O | 17.4 % |  |  |
| TVAG_075320 | vacuolar proton ATPase, putative | 7 | 8 | O | * |  |  |
| TVAG_030480 | Rhodanese-like domain containing protein | 0 | 0 | O | * |  |  |
| TVAG_311860 | Rhodanese-related sulfurtransferase-like protein | 0 | 0 | O | 4.3 % |  |  |
| TVAG_118780 | Calmoduline-putative | 0 | 0 | O | * |  |  |
| TVAG_026290 | Oxysterol-binding protein, putative | 0 | 1 | O | 17.4 % |  |  |
| TVAG_229870 | dihydrofolate synthase/folylpolyglutamate synthase, putative | 0 | 0 | O | 30.4 % |  |  |
| TVAG_277050 | Citrate lyase beta chain, putative | 0 | 0 | O | 4.3 % |  |  |
| TVAG_321030 | CoA binding domain containing protein, long chain Acyl-CoA synthetase family protein | 0 | 0 | O | 26.1 % |  | Δ |
| TVAG_342900 | NAD dependent epimerase/dehydratase, putative/Isoflavone reductase | 0 | 0 | M | 30.4 % |  |  |
| TVAG_367660 | ABC transporter, putative | 7 | 6 | S | 4.3 % | [O](../../../../C:%5CDocuments%20and%20Settings%5Cvsichni%5CDokumenty%5CMy%20Dropbox%5CRada%5CTachezy%5CPaper%5CTachezy%20Trichomonas%20paper%5CTabulka%20+obrázky%5CObrázky%5CTvABC.tif) |  |
| TVAG_239840 | MFS transporter | 11 | 12 | O | 0 | [O](../../../../C:%5CDocuments%20and%20Settings%5Cvsichni%5CDokumenty%5CMy%20Dropbox%5CRada%5CTachezy%5CPaper%5CTachezy%20Trichomonas%20paper%5CTabulka%20+obrázky%5CObrázky%5CTvMSF-1.tif) |  |

**Table S4C. Identified unknown proteins.**

| **Identification** | | **Structure** | | **Cell localization** | | | **Signal** |
| --- | --- | --- | --- | --- | --- | --- | --- |
| Accession number | Name | TMHMM | MEMSAT3 | TargetP | PsortII | Exp. Local. |  |
|  |  | TM No. | TM No. |  | mit% |  |  |
| TVAG_022120 | Unknown | 0 | 0 | O | * |  |  |
| TVAG_026100 | Unknown | 0 | 0 | O | 8.7 % |  | Δ |
| TVAG_028050 | Unknown | 0 | 0 | O | 17.4 % |  |  |
| TVAG_038870 | Unknown | 0 | 0 | O | 8.7 % |  |  |
| TVAG_044000 | Unknown | 0 | 0 | O | 4.3 % |  |  |
| TVAG_067030 | Unknown | 0 | 0 | O | 39.1 % |  | Δ |
| TVAG_074260 | Unknown | 0 | 0 | O | * |  |  |
| TVAG_089110 | Unknown | 0 | 0 | O | 8.7 % |  |  |
| TVAG_090740 | Unknown | 0 | 0 | O | * |  |  |
| TVAG_102740 | Unknown | 0 | 0 | O | 30.4 % |  |  |
| TVAG_103110 | Unknown | 0 | 1 | O | 17.4 % |  |  |
| TVAG_104680 | Unknown | 1 | 1 | S | * |  | Ct |
| TVAG_113880 | Unknown | 0 | 0 | O | * |  |  |
| TVAG_140620 | Unknown | 0 | 0 | O | 21.7 % |  |  |
| TVAG_165290 | Unknown | 0 | 0 | O | * |  |  |
| TVAG_165320 | Unknown | 0 | 0 | O | 17.4 % |  |  |
| TVAG_178820 | Unknown | 0 | 0 | O | 17.4 % |  |  |
| TVAG_197920 | Unknown | 0 | 0 | S | * |  |  |
| TVAG_209310 | Unknown | 0 | 0 | O | 13.0 % |  |  |
| TVAG_225570 | Unknown | 0 | 0 | O | 8.7 % |  |  |
| TVAG_225930 | Unknown | 0 | 0 | O | 17.4 % |  |  |
| TVAG_237550 | Unknown | 0 | 0 | O | 21.7 % |  |  |
| TVAG_241570 | Unknown | 0 | 0 | O | * |  |  |
| TVAG_249920 | Unknown | 0 | 0 | O | 4.3 % |  |  |
| TVAG_301190 | Unknown | 0 | 0 | O | * |  |  |
| TVAG_321550 | Unknown | 0 | 0 | O | 8.7 % |  |  |
| TVAG_334260 | Unknown | 0 | 0 | S | * |  |  |
| TVAG_343040 | Unknown | 0 | 0 | O | * |  |  |

**Table S4D. Identified unknown proteins.**

| **Identification** | | **Structure** | | **Cell localization** | | | **Signal** |
| --- | --- | --- | --- | --- | --- | --- | --- |
| Accession number | Name | TMHMM | MEMSAT3 | TargetP | PsortII | Exp. Local. |  |
|  |  | TM No. | TM No. |  | mit% |  |  |
| TVAG_392650 | Unknown | 0 | 0 | O | 52.2 % |  |  |
| TVAG_415360 | Unknown | 0 | 0 | O | * |  |  |
| TVAG_416630 | Unknown | 0 | 0 | O | 4.3 % |  |  |
| TVAG_430700 | Unknown | 0 | 0 | O | 21.7 % |  |  |
| TVAG_431100 | Unknown | 0 | 0 | O | 4.3 % |  |  |
| TVAG_433120 | Unknown | 0 | 0 | O | 4.3 % | [O](../../../../C:%5CDocuments%20and%20Settings%5Cvsichni%5CDokumenty%5CMy%20Dropbox%5CRada%5CTachezy%5CPaper%5CTachezy%20Trichomonas%20paper%5CTabulka%20+obrázky%5CObrázky%5CTvMdv.tif) |  |
| TVAG_437350 | Unknown | 0 | 0 | O | 17.4 % |  |  |
| TVAG_442170 | Unknown | 0 | 0 | O | 21.7 % |  |  |
| TVAG_445430 | Unknown | 0 | 0 | O | * |  |  |
| TVAG_450060 | Unknown | 0 | 0 | O | 34.8 % |  |  |
| TVAG_454330 | Unknown | 0 | 0 | O | 13.0 % |  |  |
| TVAG_483050 | Unknown | 0 | 0 | O | * |  |  |
| TVAG_483980 | Unknown | 0 | 0 | O | 8.7 % |  |  |
| TVAG_493810 | Unknown | 0 | 0 | O | 4.3 % |  |  |
| TVAG_521380 | Unknown | 0 | 0 | O | * |  |  |
| TVAG_547420 | Unknown | 0 | 0 | M | 11.1 % |  |  |
| TVAG_607400 | Unknown | 0 | 0 | M | 17.4 % |  |  |

Proteins were manually annotated based on searches in TrichDB, Uniprot, and PFAM A+B (Table S2). Protein structure was predicted using TMHMM and MEMSAT3; subcellular location was predicted using TargetP and PsortII. TM No., number of predicted transmembrane α-helixes. M, predicted location in mitochondria. S - predicted proteins of secretory pathway; O, predicted location in other compartments; Mit%, probability percentage of mitochondrial location; *, mitochondrial location was not predicted; Exp. Local., experimental location; O, localization of HA-tagged proteins in cytosol and other cell compartments was observed by immunofluorescence microscopy. Signal: Δ indicates N-terminal targeting sequence identified by Hunter; Ct, C-tail anchor detected [Fig S2].
